# Recent advances in targeting the “undruggable” proteins: from drug discovery to clinical trials

**DOI:** 10.1038/s41392-023-01589-z

**Published:** 2023-09-06

**Authors:** Xin Xie, Tingting Yu, Xiang Li, Nan Zhang, Leonard J. Foster, Cheng Peng, Wei Huang, Gu He

**Affiliations:** 1 State Key Laboratory of Southwestern Chinese Medicine Resources, College of Medical Technology and School of Pharmacy, Chengdu University of Traditional Chinese Medicine, 611137 Chengdu, China; 2 Michael Smith Laboratories, University of British Columbia, Vancouver, BC V6T 1Z4 Canada; 3 Department of Dermatology and State Key Laboratory of Biotherapy, West China Hospital, Sichuan University, 610041 Chengdu, China

**Keywords:** Medicinal chemistry, Target validation

## Abstract

Undruggable proteins are a class of proteins that are often characterized by large, complex structures or functions that are difficult to interfere with using conventional drug design strategies. Targeting such undruggable targets has been considered also a great opportunity for treatment of human diseases and has attracted substantial efforts in the field of medicine. Therefore, in this review, we focus on the recent development of drug discovery targeting “undruggable” proteins and their application in clinic. To make this review well organized, we discuss the design strategies targeting the undruggable proteins, including covalent regulation, allosteric inhibition, protein–protein/DNA interaction inhibition, targeted proteins regulation, nucleic acid-based approach, immunotherapy and others.

## Introduction

Owing to the rapid development of molecular biology, tremendous progress has been made in the past decades to uncover key biomacromolecules essential for the occurrence and progression of diseases, providing an effective approach to drug discovery.^1,2^ These biomacromolecules, including kinases, receptors and channel proteins, are characterized by their tight relation to disease development, specific hydrophobic pockets for binding with ligands, and functional changes after binding.^3^ Such targets defined as “druggable,” which means could be targeted pharmacologically, are instrumental to the development of modern medicinal science, leading to evidence-based drug design.^4,5^

The elucidation of disease mechanisms has been the key to innovative treatments. Thanks to the rise of genomics and proteomics, numerous clinically meaningful targets have been found in human disorders. However, as traditional medicinal chemistry concentrates on druggable targets, increasing disease-related targets have been discovered with few characteristics of conventional druggable targets, namely “undruggable”.^6–8^ The term “undruggable” refers to target proteins whose functional interfaces are flat and lack defined pockets for ligand interaction, making rational drug design a huge challenge.^6^ Despite this, such proteins still belong to drug targets. A typical example of an “undruggable” target is KRAS, one of the most frequently mutated oncogene proteins, with varying mutation rates in different types of solid tumors. It has experienced a long clinical drug vacancy due to its shallow pocket on the surface, which has an undesired polarity.^9^ Nevertheless, targeting such undruggable targets has been considered also a great opportunity for treatment of human diseases, and has attracted substantial efforts in the field of medicine. Surprisingly, in 2021, after unremitting efforts, a milestone was achieved: the KRAS^G12C^ inhibitor sotorasib was approved by the FDA for a specific subgroup of patients with non-small cell lung cancer (NSCLC),^10^ verifying that targeting “undruggable” proteins is worthwhile.

With the deepening research on “undruggable” targets, various molecules sharing similar undruggable features are gradually being divided into the following categories.^3^ (1) ***Small GTPases***. The RAS family proteins, including KRAS, HRAS and NRAS, belong to small GTPases. For a long time, these RAS family oncoproteins were considered “undruggable” due to the lack of pharmacologically targetable pockets on surface. Although the stalemate is changing with the emergence of approved preclinical even clinical drugs for specific cancers, drug resistance poses another challenge to the application of KRAS inhibitors.^11,12^ (2) ***Phosphatases***. As kinases is a classic representative of “druggable” targets with great significance in modulating cell motility, phosphatases are their counterparts, playing a pivotal role in the regulating cellular dynamics by catalyzing the removal of phosphate from proteins, including serine, threonine and tyrosine residues.^13^ According to structural characteristics, phosphatases has been classified into two types: protein tyrosine phosphatases (PTPs) and protein serine/threonine phosphatases (PSTPs). Unfortunately, due to the structural similarity sharing within each category of phosphatases, low selectivity and inescapable side effects have greatly hindered the progress of drug discovery.^14^ (3) ***Transcription factors (TFs)***. A variety of human disorders are related to dysregulation of TFs involved in numerous biological processes, most of which cannot be targeted by conventional small molecules due to their structural heterogeneity and deficiency of tractable binding sites.^15,16^ Targeting defined TFs and overcoming drug resistance have been identified as challenging yet promising research hotpots in the medicinal field, particularly in the areas of cancers and neurodegenerative diseases. Notable TFs include p53, Myc, estrogen receptor (ER), androgen receptor (AR), which are involved in the pathological process of neoplasm, X-box-binding protein 1 (XBP1), nuclear factor erythroid 2-related factor (NRF2) in age-related diseases and neurodegenerative diseases, and NF-κB, BTB, CNC homology (BACH), EB, E3 in immunological diseases. Current research is primarily focused on targeting p53 and Myc.^17^ (4) ***Epigenetic targets****.* Epigenetics refers to heritable changes in gene expression or cellular phenotype that occur without altering the DNA sequence. Epigenetic targets play a crucial role in regulating gene expression patterns and have implications in various biological processes and diseases. The main types of epigenetic modifications include DNA Methylation, Histone Modifications, Non-coding RNAs, Chromatin Remodeling and other Epigenetic Enzymes. Understanding and targeting these epigenetic targets have the potential to unravel the mechanisms underlying various diseases, including cancer, neurological disorders, and cardiovascular diseases.^18^ (5) ***Other proteins***. Protein–protein interactions (PPIs) and their networks are of great significance in biological processes and in the regulation of the cell cycle, offering another potential avenue for treatments of complex diseases. RAS and TFs such as p53 and Myc are also subjected to PPI networks. A portion of PPIs, those with flat interaction surfaces, are found to be more difficult to target than other PPIs, making them “undruggable” to a certain extent. Classic PPI-related proteins include anti-apoptotic members of the B-cell lymphoma-2 (Bcl-2) family. Additionally, intrinsically disordered proteins with highly dynamic structures, which interact with various protein partners, are also considered to be undruggable PPI proteins due to a lack of binding cavities.^19^

Nowadays, in the face of so-called “undruggable” targets, academia has developed dozens of innovative approaches and pharmaceutical companies have invested billions of dollars, changing the term from “undruggable” to “difficult to drug” or “yet to be drugged,” resulting in several approved drugs and emerging potent chemical entities.^20–23^ According to the mechanism of undruggable proteins, some major strategies for drug design has been formed correspondingly, including covalent inhibition, allosteric inhibition, PPIs inhibition, targeted proteins regulation, nucleic acid-based approaches, immunotherapy and etc.^3,6^ By adopting cutting-edge technologies such as fragment-based drug discovery (FBDD), a method leveraging stochastic screening and structure-based design; computer-aided drug design (CADD), simulating and computationally predicting drug-target interactions to screen, design and optimize lead compounds; virtual screening (VS), an in silico screening technique premised on the lock-and-key model of drug–target compatibility; DNA-encoded libraries (DELs), a collection of small molecules conjugated to DNA tags for efficient bio-target screening; targeting allosteric sites, inactivating targets by binding variable loci, etc., strategies for drug design have been well developed systematically.^24–26^ The form of existing entities includes bifunctional molecules, covalent drugs, peptide-based drugs, protein-based drugs, and therapeutic RNAs.^3,6,27^ In this review, we will illustrate the recent development of drug discovery targeting “undruggable” proteins, according to the types of design strategies.

## Covalent regulation

Covalent inhibitors, also known as irreversible inhibitors, are a class of inhibitors that bind to amino acid residues of target proteins through covalent bonds formed by mildly reactive functional groups to confer additional affinity, compared to that of non-covalent inhibitors, which achieve binding and inhibition of target proteins through non-covalent interactions such as hydrogen bond and van der Waals force, resulting in low selectivity and inhibition ability.^28–32^ Hence, covalent inhibitors have the advantage of sustained inhibition and a longer residence time compared to non-covalent inhibitors because the covalently bound target is continuously inhibited until protein degradation and regeneration.^28^ At the same time, covalent inhibitors can also reduce dosage and improve compliance, avoiding some potential resistance mechanisms.^33,34^ Due to the recognition of potential benefits of covalency, rational design of covalent drugs has contributed to overcome drug resistance induced by mutated kinases and treat diseases related to hot spot targets. For instance, nirmatrelvir, a part of Paxlovid that has been approved for emergency use in COVID-19, is a covalent inhibitor of M^pro^ of SARS-CoV-2, highlighting the significance of cysteine-reactive covalent functional groups in targeting the protease active site of M^pro^.^35^

As non-covalent interactions are relatively weak, deep grooves on surface of target proteins that allow small molecules to bind effectively are required to guarantee the affinity of non-covalent inhibitors.^36–38^ Whereas, covalent inhibitors could target undruggable proteins which lack surface “pockets”, offering the potential to expand the therapeutic range. In this area, the approval of the KRAS inhibitor, sotorasib, is a remarkable milestone both in the development of covalent drugs and the progress of drugging the undruggable.

**Fig. 1:**
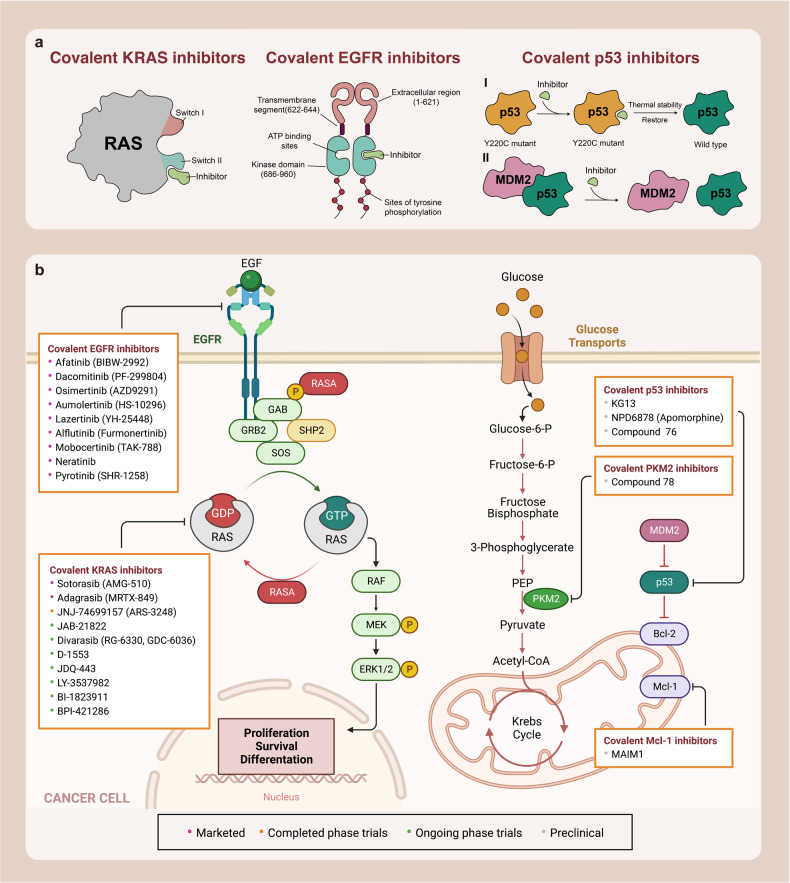
Covalent modulators targeting undruggable proteins. Covalent inhibitors bind to amino acid residues of target proteins through covalent bonds formed by mildly reactive functional groups to confer additional affinity. **a** Binding modes of selected covalent modulators: covalent KRAS inhibitors bind to the cystine of KRAS^G12C^ mutants to reduces the affinity between GTP and KRAS, thereby locking the KRAS^G12C^ mutant in an inactivated state; covalent EGFR inhibitors bind to Cys797 at ATP binding site of EGFR, showing high affinity for T790M mutants and solving resistance; covalent p53 stabilizers bind to p53-Y220C mutant to restore thermal stability to the wild-type level, or prevent the interaction between MDM2 and p53. **b** Map of marketed, clinical and preclinical covalent inhibitors in signaling pathways

Here, we introduce how covalent drugs act on acknowledged undruggable proteins and kinases no longer druggable due to mutations, elaborating on the marketed drugs, drugs in clinical trials and lead compounds developed by the covalent inhibition strategy (Fig. 1).

### Covalent KRAS inhibitors

KRAS plays a crucial role in intracellular signaling pathways that are involved in cell growth and survival.^39^ It is the most dominant mutated subtype in the RAS family and is responsible for 85% of RAS gene-driven cancers, particularly in pancreatic, colorectal, and lung cancers.^40–42^ KRAS alternates between inactive GDP-bound states and active GTP-bound states. KRAS alternates between inactive GDP-bound states and active GTP-bound states, regulated by two types of factors: ① Guanine nucleotide exchange factors (GEFs), such as SOS proteins, which catalyze the transition between KRAS and GTP-bound states; ② GTPase activating proteins (GAPs), which promote the hydrolysis of GTPs bound to KRAS, resulting in the conversion of the active state to one terminating in GDP, thereby inhibiting the activity of KRAS.^43^

Targeting KRAS directly presents many difficulties. Its wide range of actions and its normal activity being required for many normal cell functions make it difficult to inhibit. Furthermore, KRAS has high homology with NRAS and HRAS, and its currently known active functional domains of KRAS are mainly pocket-shaped, combining KRAS with either GDP or GTP.^44^ Unlike protein kinase, which has a weak affinity with ATP, KRAS has a binding affinity with GTP and GDP at the pM level, making it difficult to compete as effectively as protein kinase inhibitors. In summary, KRAS protein is a featureless, nearly spherical structure with no obvious binding sites, making it difficult to synthesize compounds that can effectively target and inhibit its activity.^45^ Long impenetrable, KRAS has become a byword for “undruggable” targets in oncology drug development. Common mutation sites in KRAS include codons 12, 13 and 61, with codon 12 being the most common mutation site.^46–48^ The most frequent mutant forms were KRAS^G12D^ (41%), KRAS^G12V^ (28%), and KRAS^G12C^ (14%).^49^

In recent years, breakthroughs in covalent inhibitors discovered through electrophile-first approaches made it possible to target KRAS^G12C^ mutants.^50^ Cysteine is located at codon 12 of KRAS^G12C^, making it possible to selectively target mutant KRAS covalently. Importantly, KRAS active sites lack cysteine, and KRAS^G12C^ can be specifically inhibited in a covalent manner. In KRAS^G12C^ mutants, small molecules covalently bound to the mutant cystine have been found to bind more readily to GDP-bound KRAS proteins. This binding reduces the affinity between GTP and KRAS, thus preventing GEF from catalyzing the replacement of GDP with GTP, thereby locking the KRAS^G12C^ mutant in an inactivated state.^51^ The discovery of this binding “pocket” on KRAS^G12C^ mutants has sparked the development of several small-molecule covalent inhibitors specifically targeting KRAS^G12C^ mutants. Of these, sotorasib and adagrasib are in clinical use, and more than ten are undergoing clinical trials (Table 1).

**Table 1:** Covalent modulators targeting undruggable proteins

Compound name and structure	Target	Cancer cell line (activity)	Indications	Status/clinical trial identifier	Ref.
Sotorasib (AMG-510) (**1**)	KRAS^G12C^	–	Colorectal cancer, NSCLC	*Marketed*	^54^
Adagrasib (MRTX-849) (**2**)	KRAS^G12C^	–	NSCLC	*Marketed*	^57^
JAB-21822 (**3**)^a^	KRAS^G12C^	–	Colorectal cancer, NSCLC	*Ongoing* NCT05288205(I/II), NCT05276726(I/II), NCT05194995(I/II), NCT05002270(I/II), NCT05009329(I/II)	^61^
JNJ-74699157 (ARS-3248) (**4**)^a^	KRAS^G12C^	–	Colorectal cancer, NSCLC	*Completed* NCT04006301(I)	^66^
Divarasib (RG-6330, GDC-6036) (**5**)	KRAS^G12C^	–	Colorectal tumor, NSCLC	*Ongoing* NCT04449874(I)	^67^
D-1553 (**6**)^a^	KRAS^G12C^	–	Colorectal cancer, NSCLC	*Ongoing* NCT05383898(I/II), NCT04585035(I/II), NCT05492045(I/II), NCT05379946(I/II)	^70^
JDQ-443 (**7**)	KRAS^G12C^	–	NSCLC	*Ongoing* NCT05132075(III), NCT05445843(II), NCT04699188(I/II), NCT05329623(I), NCT05358249(I/II))	^72^
LY-3537982 (**8**)^a^	KRAS^G12C^	–	Colorectal cancer, NSCLC, etc.	*Ongoing* NCT04956640(I)	^74^
BI-1823911 (**9**)^a^	KRAS^G12C^	–	Biliary cancer, colorectal cancer, NSCLC, etc.	*Ongoing* NCT04973163(I)	^75^
BPI-421286 (**10**)^a^	KRAS^G12C^	–	Advanced solid tumor	*Ongoing* NCT05315180(I)	^77^
RMC-6291 (**11**)^a^	KRAS^G12C^	–	Colorectal cancer, NSCLC, etc.	*Ongoing* NCT05462717(I)	^78^
IBI-351 (GFH-925, GF-105) (**12**)^a^	KRAS^G12C^	–	Colorectal cancer	*Ongoing* NCT05497336(I), NCT05699993(I), NCT05688124(I), NCT05626179(I), NCT05504278(I)	^79^
RM-018 (**13**)	KRAS^G12C^	H358 (IC_50_ = 1.4–3.5 nM)	–	*Preclinical*	^81^
RM-032 (**14**)^a^	KRAS^G12C^	–	–	*Preclinical*	^81^
RMC-9805 (**15**)^a^	KRAS^G12C^	HPAC (IC_50_ = 7 nM)	–	*Preclinical*	^82^
RMC-8839 (**16**)^a^	KRAS^G12C^	–	–	*Preclinical*	^83^
6H05 (**17**)	KRAS^G12C^	–	–	*Preclinical*	^86^
2E07 (**18**)	KRAS^G12C^	–	–	*Preclinical*	^86^
ARS-853 (**19**)	KRAS^G12C^	H358 (IC_50_ = 1.6 μM)	–	*Preclinical*	^89^
ARS-1620 (**20**)	KRAS^G12C^	H358 (IC_50_ = 0.15 μM)	–	*Preclinical*	^84^
Gray series compounds (**21**–**23**)	KRAS^G12C^	H358 (IC_50_ = 26.6 μM)	–	*Preclinical*	^90–92^
G12Si-5 (**24**)	KRAS^G12S^	A549 (IC_50_ = 2.4 μM)	–	*Preclinical*	^93^
G12R inhibitor-4 (**25**)	KRAS^G12R^	–	–	*Preclinical*	^94^
1_AM, 2_AM, 3_AM, 4_AM (**26**–**29**)	KRAS^G12C^	H358 (IC_50_ = 0.73–2.98 μM)	–	*Preclinical*	^95^
Fell series compounds (**30**–**33**)	KRAS^G12C^	H358 (IC_50_ = 0.07–7.6 μM)	–	*Preclinical*	^96^
Lanman series compounds (**34**–**36**)	KRAS^G12C^	LNCaP (IC_50_ = 0.012–0.211 μM)	–	*Preclinical*	^98^
Shin series compounds (**37**–**40**)	KRAS^G12C^	MIA PaCa-2 (IC_50_ = 0.219–11.4 μM)	–	*Preclinical*	^97^
APG-1842 (**41**)^a^	KRAS^G12C^	H358 (IC_50_ = 4 nM)	–	*Preclinical*	^653^
EB-160 (**42**)^a^	KRAS^G12C^	H358 (IC_50_ = 17.54 nM)	–	*Preclinical*	^654^
ERAS-3490 (**43**)^a^	KRAS^G12C^	H358 (IC_50_ = 1.4–82 nM)	–	*Preclinical*	^655^
VRTX-126 (**44**)^a^	KRAS^G12C^	–	–	*Preclinical*	^656^
Afatinib (Giotrit^TM^, BIBW-2992) (**45**)	EGFR	–	Metastatic NSCLC, NSCLC	*Marketed*	^122^
Dacomitinib (Vizimpro^TM^, PF-299804) (**46**)	EGFR	–	Metastatic NSCLC	*Marketed*	^128^
Osimertinib (AZD9291) (**47**)	EGFR	–	Metastatic NSCLC, NSCLC	*Marketed*	^139^
Aumolertinib (Almonertinib, HS-10296) (**48**)	EGFR	–	Metastatic NSCLC	*Marketed*	^141^
Lazertinib (YH-25448) (**49**)	EGFR	–	Metastatic NSCLC	*Marketed*	^142^
Alflutinib (Furmonertinib) (**50**)	EGFR	–	Metastatic NSCLC	*Marketed*	^146^
Mobocertinib (TAK-788) (**51**)	EGFR	–	Metastatic NSCL	*Marketed*	^150^
Olmutinib (HM61713, BI-1482694) (**52**)	EGFR	–	NSCLC	*Marketed*	^152^
Neratinib (**53**)	EGFR	–	Breast cancer	*Marketed*	^155^
Pyrotinib (SHR-1258) (**54**)	EGFR	–	Breast cancer	*Marketed*	^156^
Avitinib (Abivertinib, AC0010) (**55**)	EGFR	–	Metastatic NSCLC	*Being applied for approval*	^159^
Oritinib (SH-1028) (**56**)	EGFR	–	NSCLC	*Being applied for approval*	^163^
Sunvozertinib (DZ-0586, DZD-9008) (**57**)	EGFR	–	Metastatic NSCLC	*Being applied for approval*	^166^
Rezivertinib (BPI-7711) (**58**)	EGFR	–	Metastatic NSCLC	*Being applied for approval*	^169^
Olafertinib (CK-101, RX518) (**59**)	EGFR	–	NSCLC	*Completed* NCT02926768(I)	^172^
Nazartinib (EGF816, NVS-816) (**60**)	EGFR	–	Advanced solid tumor, metastatic NSCLC	*Ongoing* NCT03040973(II)	^173^
Allitinib (AST-1306) (**61**)	EGFR	–	Metastatic breast cancer, NSCLC	*Ongoing* NCT04671303(II)	^177^
ES-072 (**62**)^a^	EGFR	–	Metastatic NSCLC	*Ongoing* CTR20180074(I)	^182^
YK-029A (**63**)^a^	EGFR	–	NSCLC	*Ongoing* CTR20180350(I)	^185^
Canertinib (CI-1033, PD-183805) (**64**)	EGFR	–	Breast cancer, head and neck neoplasms, NSCLC, ovarian cancer	*Terminated* NCT00051051(II), NCT00174356(I), NCT00050830(II)	^187^
Rociletinib (Xegafri^TM^, CO-1686) (**65**)	EGFR	–	NSCLC	*Terminated* NCT02322281(III), NCT02186301(II/III), NCT02147990(II), NCT02705339(II), etc.	^191^
Naquotinib (ASP8273) (**66**)	EGFR	–	Metastatic NSCLC, NSCLC	*Terminated* NCT02674555(I), NCT02588261(III), NCT03082300(I), NCT02113813(II)	^195^
Mavelertinib (PF-06747775) (**67**)	EGFR	–	NSCLC	*Terminated* NCT02349633(I/II)	^198^
CL-387785 (EKI-785, WAY-EKI 785) (**68**)	EGFR	A432 (IC_50_ = 67 ± 7.6 nM)	–	*Preclinical*	^199^
WZ 4002 (**69**)	EGFR	NIH-3T3	–	*Preclinical*	^203^
PD series compounds (**70**–**73**)	EGFR	A431	–	*Preclinical*	^204^
KG13 (**74**)	p53^Y220C^	NUGC-4 (IC_50_ = 7.1 μM)	–	*Preclinical*	^214^
NPD6878 (Apomorphine) (**75**)	p53-MDM2 PPI	–	–	*Preclinical*	^218^
Hamachi’s research Compound (**76**)	p53-HDM2 PPI	SJSA1, MCF7	–	*Preclinical*	^223^
MAIM1 (**77**)	Mcl-1	–	–	*Preclinical*	^228^
PKM2 inhibitor Compound (**78**)	PKM2	PA-1 (IC_50_ = 0.16 μM)	–	*Preclinical*	^229^

Data collected from https://clinicaltrials.gov [last accessed March 2023]

^a^The chemical formula was not disclosed

#### Marketed covalent drugs for KRAS inhibition

Sotorasib (AMG-510). In May 2021, the U.S. Food and Drug Administration (FDA) granted accelerated approval to Lumakras (sotorasib, AMG-510), a targeted anticancer drug, for the treatment of NSCLC patients with a KRAS^G12C^ mutation.^52^ It also became the first targeted drug for the treatment of KRAS gene mutation in the world, breaking the “undruggable” dilemma and marking a milestone in medical history. In collaboration with Carmot Therapeutics, Amgen investigators have discovered sotorasib (AMG-510) (1), the first selective small molecule KRAS^G12C^ inhibitor to enter clinical trials, through a structure-based design.^53^ Sotorasib specifically and irreversibly inhibits KRAS^G12C^ by binding to GDP and locking KRAS in an inactive state. In addition, sotorasib has been shown to strongly inhibit phosphorylation of ERK protein in KRAS^G12C^ cells, thereby suppressing cell proliferation. Current studies on sotorasib have identified a variety of indications for its use in the treatment of adenocarcinoma, metastatic colorectal cancer and metastatic NSCLC.^53–55^ Subsequently, it has been successively approved for marketing in the European Union, Japan, and other countries. According to the most recent ACCR report, the overall remission rate of sotorasib in patients with KRAS^G12C^ mutated NSCLC was 37%, with a disease control rate of 81%, a median progression-free survival of 6.8 months, and a median duration of remission of 10.0 months.^56^

Adagrasib (MRTX-849). At the same time, another high-profile drug targeting the KRAS^G12C^ mutation with impressive clinical data has also stepped up its pace of marketing. Mirati Therapeutics and Array BioPharma have collaborated to identify an irreversible small molecule covalent inhibitor of KRAS^G12C^, adagrasib (MRTX-849) (**2**).^57^ Adagrasib binds covalently to Cys12 of KRAS^G12C^ and extends to allosteric pocket S-II P, thereby locking KRAS proteins into inactive conformations and inhibiting RAS/MAPK kinase signaling.^58^ At the maximum effective dose of 100 mg kg^−1^ d^−1^, adagrasib demonstrated dose-dependent antitumor effects against different tumor models. Adagrasib is more than 1000 times more selective to KRAS^G12C^ than wild-type KRAS and other proteins containing Cys. It has an oral bioavailability of up to 30%, with a half-life of 25 h after a single dose.^59^ At present, the study of adagrasib has revealed its potential for use in the treatment of advanced solid tumors, metastatic colorectal cancer, metastatic NSCLC, and metastatic pancreatic cancer. Adagrasib entered phase III clinical trials in January 2019, and according to the most recent ACCR report, it had an overall remission rate of 58%, with a median duration of treatment of 9.5 months and median duration of remission of 12.6 months. On December 12, 2022, the FDA granted accelerated marketing approval of adagrasib for use in an FDA-approved clinical trial to identify adult patients with locally advanced or metastatic NSCLC with KRAS^G12C^ mutations.^60^

#### Covalent KRAS inhibitors in clinical trials

In addition to the marketed drugs, sotorasib and adagrasib, there are currently 10 clinical drugs for covalent RAS inhibitors involving 24 clinical trials, of which 23 are ongoing and 1 has been completed. JAB-21822 (**3**) is a small molecule KRAS^G12C^ covalent inhibitor developed by Jacobio. JAB-21822 can lock KRAS^G12C^ in a non-activated state and block the signal transduction of KRAS to the downstream, thus playing an antitumor role. It can be utilized for a multitude of indications, such as colorectal cancer, NSCLC, advanced solid tumor, and metastatic NSCLC, in the clinical research and development stage.^61,62^ JAB-21822 was enrolled in clinical trials in August 2018. At the 2022 ASCO annual meeting, Jacobio presented phase I clinical data from JAB-21822. As of April 2022, a total of 72 patients with advanced solid tumors had been enrolled in the trial. Among them, 32 patients with KRAS^G12C^ mutation were evaluated for efficacy, with an ORR of 56.3% (18/32) and a disease control rate (DCR) of 90.6% (29/32). In September 2022, the Center for Drug Review (CDE) of the China National Drug Administration approved a pivotal phase II trial of JAB-21822 for second-line and beyond treatment of patients with advanced or metastatic NSCLC with the KRAS^G12C^ mutation. Currently, JAB-21822 is conducting a number of simultaneous Phase I/II clinical trials in China, the United States, and Europe (NCT05288205, NCT05276726, NCT05194995, NCT05002270, NCT05009329), targeting advanced solid tumor patients with KRAS^G12C^ mutation.

Araxes, a subsidiary of Wellspring, was one of the first companies to be involved in the development of new mutation sites for KRAS. JNJ-74699157 (**4**), also called ARS-3248, is a new generation, oral, selective, covalent inhibitor of the KRAS^G12C^ subtype developed by this company. It blocks downstream signaling of KRAS^G12C^ by covalently binding to the KRAS^G12C^ complex near S-II P of the KRAS mutant protein. JNJ-74699157 has demonstrated high selectivity for the tumor-associated KRAS^G12C^ protein.^63–65^ Currently, through clinical research and development, JNJ-74699157 has been found to be effective for advanced solid tumors, metastatic NSCLC, metastatic colorectal cancer, and other indications. In May 2019, Wellspring announced that the FDA had approved an Investigational New Drug (IND) application for JNJ-74699157. Subsequently, JNJ-74699157 conducted a clinical phase I trial (NCT04006301) enrolling patients with KRAS^G12C^ positive advanced solid tumor, which was completed in July 2020 with no results posted.^66^

Divarasib (**5**) is a small molecule covalent inhibitor of KRAS^G12C^ developed by Genentech that is orally available, highly selective, and potent. It also irreversibly immobilizes KRAS^G12C^ in the inactivation state. Currently, studies on divarasib have found that it can be used in the treatment of NSCLC, advanced solid tumor, colorectal cancer, and other indications. Divarasib was officially enrolled in clinical trials in June 2020, and is currently in phase I clinical trials (NCT04449874) to evaluate its safety, pharmacokinetics and activity in patients with advanced or metastatic solid tumors with KRAS^G12C^ mutations.^67,68^ Of the 59 patients with NSCLC previously treated with divarasib monotherapy included, 57 patients had evaluable outcomes, 26 of whom were confirmed to be in partial remission (PR), with confirmed objective remission rate (ORR) of 46%. 88.1% of patients experienced at least one adverse event (AE), with the most common AEs being nausea (76.3%), diarrhea (61%), vomiting (54.2%), malaise (23.7%), and loss of appetite (15.3%). Divarasib is more selective than the already marketed sotorasib and adagrasib. According to data presented during the 2022 World Lung Cancer Congress, divarasib treated patients with KRAS^G12C^ mutation NSCLC with ORR of up to 53% (46% of which had been confirmed by imaging). Of the patients tested, 90% had been treated with platinum-based chemotherapy and 86% had received treatment with immune checkpoint inhibitors.^69^

D-1553 (**6**), an independently developed small molecule KRAS^G12C^ covalent inhibitor by Inventis. Bio., is the first oral antitumor drug targeting KRAS^G12C^ mutation to be approved for clinical trials in China.^70^ D-1553 has demonstrated excellent tumor inhibition effect and good safety in preclinical studies, making it an ideal candidate for a variety of clinical indications, such as advanced solid tumors, metastatic colorectal cancer, and metastatic NSCLC. In October 2020, D-1553 was officially registered as ready for clinical trials. Currently, a number of clinical phase I/II trials (NCT05492045, NCT05383898, NCT05379946, NCT04585035) have been initiated to evaluate the application of D-1553 in the combined treatment of NSCLC and in the treatment of solid tumors with IN10018, a highly effective and selective inhibitor of FAK. In 2022, a report on the safety and efficacy of D-1553 was presented at the WCLC Congress. No dose-limiting toxicity of D-1553 was observed in 79 patients with KRASG12C mutant NSCLC. Of these, 3 patients decreased dose due to TRAE, and 2 patients discontinued treatment due to TRAE. Among the 74 patients that could be evaluated, 28 patients had PR, 40 patients had SD, ORR was 37.8% (28/74), and DCR was 91.9% (68/74).^71^

JDQ-443 (**7**), a selective covalent inhibitor of KRAS^G12C^ developed by Novartis, was officially registered for clinical trials in January 2021. In order to overcome the resistance of other KRAS^G12C^ inhibitors, JDQ-443 covalently binds to the “Switch II pocket” of KRAS^G12C^ and irreversibly locks it into an inactive GDP binding state. Studies on JDQ-443 have revealed that it can be utilized in the treatment of advanced solid tumors, metastatic colorectal cancer, metastatic NSCLC, and other indications.^72,73^ Furthermore, JDQ-443 in conjunction with the SHP 2 inhibitor TNO-155 has demonstrated a synergistic effect in preclinical animal models, resulting in improved outcomes at lower doses.^73^ Preliminary results from a phase I/II trial of JDQ-443 in patients with advanced NSCLC (NCT04699188, NCT05132075, NCT05358249, NCT05329623) indicate an overall response rate of 57% (4/7) in those receiving the recommended dose in the phase II trial. In November 2022, Novartis launched a phase III trial (LBCTR2022055019) to compare the efficacy and safety of JDQ-443 versus TNO-155 in patients with locally advanced or metastatic KRAS^G12C^ mutated NSCLC. Currently, in addition to the suspension of enrollment in the phase I study of JDQ-443 pharmacokinetics in participants with impaired liver function (NCT05329623), other clinical trials are ongoing to evaluate the efficacy of JDQ-443 in patients with locally advanced solid tumors or metastatic KRAS^G12C^ mutations in NSCLC.

Unveiled at the 2021 American Association for Cancer Research (AACR) by Lilly, LY-3537982 (**8**) is a highly selective and effective covalent KRAS^G12C^ inhibitor. LY-3537982 (IC_50_ = 3.35 nM) demonstrated exceptionally high target inhibitory activity in KRAS^G12C^ mutated human H358 lung cancer cell lines, surpassing that of sotorasib (IC_50_ = 47.9 nM) and adagrasib (IC_50_ = 88.9 nM) by more than 10 and 25 times, respectively. Data from preclinical studies presented at the AACR in 2022 showed that the drug LY-3537982 had good activity, with inhibiting KRAS-GTP binding in lung cancer cell lines carrying the KARS^G12C^ variant. In a variety of mouse tumor models containing KRAS^G12C^ gene variants, LY-3537982 significantly inhibited tumor proliferation or even led to complete tumor regression. Now, LY-3537982 is being developed for the highest stage of research globally for indications including colorectal cancer, NSCLC, ovarian tumors, advanced solid tumors, pancreatic tumors, endometrial cancer, etc. In July 2021, LY-3537982 was registered for clinical trials and is currently in phase I clinical trial (NCT04956640) for KRAS^G12C^ mutant solid tumors.^74^

BI-1823911 (**9**), developed by Boehringer Ingelheim, is a new molecular entity compound with complete independent intellectual property rights. It is a novel, powerful and highly selective covalent irreversible KRAS^G12C^ oral small molecule inhibitor, intended for the treatment of patients with unresectable, locally advanced, or metastatic solid tumors carrying KRAS^G12C^ specific oncogene mutation. In addition, BI-1823911 is in clinical development for a variety of indications, including adenocarcinoma, metastatic lung cancer, metastatic colorectal cancer, cancer, biliary tract cancer, bile duct cancer, advanced solid tumors, metastatic NSCLC, and metastatic pancreatic cancer.^75^ In July 2021, a clinical trial application for BI-1823911 (NCT04973163) began to approve to test different doses of BI-1823911 alone and in combination with other agents in patients with various types of advanced cancer harboring KRAS mutations.^75^ The 2022 ACCR Conference focused on the preclinical combination data of BI-1823911 and the SOS1 inhibitor, BI-1701963. When BI-823911 was combined with BI-1701963, an SOS1 inhibitor, a deeper level of PD regulation was observed. By analyzing the dose-and time-dependent combination data of BI-1823911 and KRAS, it was found that BI-1823911 can induce concomitant MAPK pathway regulation, G1 cell cycle arrest, and apoptosis. Furthermore, BI-1823911 demonstrated excellent synergistic anti-proliferation activity when combined with PI3K/mTOR, EGFR inhibitors and SOS1 inhibitors.^76^

BPI-421286 (**10**) is a newly developed molecular entity by Betta Pharmaceutical with complete independent intellectual property rights. This powerful, highly selective covalent irreversible KRAS^G12C^ oral small molecule inhibitor is intended for the treatment of patients with unresectable, locally advanced, or metastatic solid tumors carrying a KRAS^G12C^ specific oncogene mutation. Preclinical data has demonstrated that BPI-421286 has consistent in vitro and in vivo biological activity, effectively inhibiting the proliferation of tumor cells carrying the KRAS^G12C^ mutation, and exhibiting a good antitumor effect in a variety of transplanted tumor models carrying the KRAS^G12C^ mutation. In April 2022, a phase I clinical trial (NCT05315180) of BPI-421286 was initiated to evaluate its efficacy in an open-marker study in patients with advanced solid tumors.^77^

The current inhibitors targeting KRAS^G12C^ are all based on a small molecule-protein binding mechanism that lock KRAS^G12C^ in an inactive state and promotes the depletion of already active KRAS^G12C^, referred to as the KRAS (OFF) mechanism. However, in the process of GTP conversion to GDP, there are still a small number of active conformations that bind to GTP, giving tumor cells a chance to exploit it. One of the primary reasons why KRAS mutants are difficult to target is that they lack pockets on their surface which would be suitable for binding small molecules, making it difficult to develop effective therapeutic strategies. Research has demonstrated that the activated KRAS protein binds to cyclophilin A, a companion protein, to form pockets that can be targeted by small molecules, providing a potential avenue for the development of a novel type of KRAS inhibitor, aptly named KRAS (ON) inhibitors. The mechanism of KRAS (ON) inhibitors is to prevent cyclophilin A from binding to KRAS in an activated state, thus inhibiting the already activated KRAS from exerting its biological effects and effectively cutting off downstream signaling. This approach may be more effective than KRAS (OFF) inhibitors.^41^ Currently, there is a small molecule drug based on RAS (ON) mechanism, RMC-6291 (**11**) developed by Warp Drive Bio, which has entered the phase I clinical trial (NCT05462717) in July 2022 for the treatment of solid tumors. RMC-6291 is an orally administered, selective covalent inhibitor designed to treat KRAS^G12C^-driven mutants in cancer patients. In April 2022, the company reported on the AACR that RMC-6291 demonstrated superior preclinical efficacy compared to adagrasib.^78^

IBI-351 (GFH-925, GF-105) (**12**), developed by Innovent Biologics, is a novel, irreversible covalent inhibitor of KRAS^G12C^ mutation. It is being developed for indications such as gastrointestinal tumors, NSCLC, solid tumors, solid tumors with KRAS^G12C^ mutations, colorectal cancer, non-squamous NSCLC, etc. In August 2022, IBI-351 was officially registered for clinical trials. Phase I trials of IBI351 in combination with other drugs (NCT05626179, NCT05504278, NCT05497336, NCT05699993, and NCT05688124) are also underway. For example, the efficacy and safety of IBI-351 in combination with sintilimab ± chemotherapy to treat patients with advanced non-squamous NSCLC of KRAS^G12C^ mutation are being evaluated, as well as the combination of IBI-351and cetuximab in the treatment of KRAS^G12C^ mutated metastatic colorectal cancer.^79^ Of the 55 evaluable NSCLC patients, 28 achieved a PR, resulting in an investigator-assessed ORR of 50.9% and DCR of 92.7%. In patients with NSCLC, the ORR assessed by the investigator was 61.9% (13/21) and the DCR was 100% at the recommended dose.^80^

#### Covalent KRAS inhibitors in preclinical research and lead compounds

There are also several compounds in preclinical development as covalent inhibitors of various subtypes of RAS (ON). RM-018 (**13**), developed by Revolution Medicines, covalently binds to the activated state of KRAS^G12C^ mutants, forms a ternary complex with cyclophilin A and KRAS^G12C^, thereby inhibiting their activity. Meanwhile, RM-018 retained the ability to bind and inhibit KRAS^G12C/Y96D^, thus overcoming drug resistance.^81^ RM-032 (**14**) is another inhibitor of KRAS^G12C^ (ON) mutation, discovered by Jesse Boumelha and his colleagues, with double selectivity for both KRAS^G12C^ (ON) and NRAS^G12C^ (ON). In vitro, RM-032 was shown to improve the persistence of RAS pathway signaling and cell proliferation inhibition in KRAS^G12C^ tumor cells compared to KRAS^G12C^ (OFF) inhibition. RMC-9805 (**15**) is a selective, orally-administrated covalent inhibitor of KRAS^G12D^ (ON) inhibitor that has been developed by Revolution Medicines for the treatment of patients with colorectal cancer (CRC), pancreatic cancer, or NSCLC. Studies have demonstrated that RMC-9805 effectively inhibits the growth of KRAS^G12D^ mutant cancer cells, inducing cell apoptosis, with low off-target reactivity. Tumor regression can be achieved by repeated oral administration in a KRAS^G12D^-driven pancreatic tumor xenograft model. However, it has no inhibitory effect on BRAFV600E dependent cells.^82^ RMC-8839 (**16**) is the first orally-administered, mutant-selective, covalent KRAS^G13C^ inhibitor developed by Revolution Medicines. This compound directly targets KRAS^G13C^, an important therapeutic target for patients with lung cancer and some colorectal cancers who are not currently being served by any RAS-targeted drugs.^83^

Due to the significance of drug design for RAS, in addition to being inspired by the breakthrough in drugging KRAS, dozens of compounds are in preclinical research, with thousands of compounds being considered as candidates. Of these, ARS-1620 is the first publicly disclosed, drug-like KRAS^G12C^ inhibitor, with profound implications for its development history and significance.^84^ In 2012, Kevan M. Shokat, a professor from the University of California, and Troy Wilson, the President and CEO of Kura Oncology, co-founded Araxes Pharma to develop covalent inhibitors targeting the KRAS^G12C^. In 2013, Shokat and co-workers discovered a new strategy where they used covalent inhibitors to bind to the cysteine of KRAS^G12C^ mutation, and screened out two lead compounds, 6H05 (**17**) and 2E07 (**18**), by utilizing “tethering” technique.^47,85,86^ In the research of structure-activity relationships of 6H05 derivatives, a new allosteric pocket, S-IIP, was identified in KRAS and exploited in further structural optimization.^59,86^ Structural analysis showed that 6H05 derivatives formed conformational changes that hindered PPI between RAS and SOS mediated by SW-I and SW-II and further impaired SOS catalyzed nucleotide exchange. PPI between RAS and RAF is also destroyed due to the interruption of residue interaction at the interface and the interruption of the transition between active and inactive forms of RAS.^87,88^ Moreover, the discovery of allosteric binding site S-II P has become a key point in drug design. The landmark findings are published on Nature. With ongoing development, Wellspring Biosciences—a subsidiary of Araxes Pharma—reported early results with the KRAS^G12C^ inhibitor ARS-853 (**19**) in Science and Cancer Discovery in 2016.^89^ Due to the undesirable pharmacokinetic properties and poor druggability of ARS-853, they further reported ARS-1620 (**20**) on Cell with disclosed structure, which has been embraced as a starting point by numerous drugmakers for further development. The structural optimizations of marketed sotorasib (AMG-510) and adagrasib (MRTX-849), as well as JNJ-74699157 (ARS-3248) in the clinical trial, which are based on a covalent binding strategy, have been inspired by the structure of ARS-1620. To date, dozens of compounds structurally derived from sotorasib (AMG-510), adagrasib (MRTX-849), and ARS-1620 have been developed and patented by various drugmakers, many of which are me-too and fast-follow compounds.

Gray et al. developed covalent kinase inhibitors based on GDP/GTP binding sites, providing a new idea for the study of KRAS^G12C^ inhibitors, and obtained a series of nucleotide covalent KRAS^G12C^ inhibitors.^90^ They first designed a series of substrate competition-related covalent inhibitors targeting catalytic sites based on their GDP-based structure, and SML-8-73-1 (**21**) was identified as the main candidate. In simulated cell conditions, the binding efficiency of SML-8-73-1 was measured in the presence of GDP/GTP of 1 mmol L^−1^. The results showed that after incubation for 2 h, the substrate competitive binding of SML-8-73-1 was more than 95% KRAS^G12C^. However, SML-8-73-1 contains two negatively charged phosphate groups, making it difficult to cross the cell membrane.^86,90^ Therefore, SM-10-70-1 (**22**) was synthesized by modifying phosphoric acid groups of SML-8-73-1 with “caging” technology.^91^ SM-10-70-1 showed increased cellular permeability and competitively inhibited KRAS^G12C^ through covalent binding. In addition, KRAS-dependent signaling pathways, such as the Akt and Erk pathways, are also inhibited. Moreover, the ability of SM-10-70-1 to exhibit anti-proliferative activity was demonstrated in several cancer cell lines expressing the KRAS^G12C^ mutation. However, its effective rate and selectivity remain to be further improved. As a result, new SARs research was continued and promising XY-02-075 (**23**) was obtained. The chemical and enzymatic stability of XY-02-075 is greatly improved by methylene substitution of the central oxygen in the phosphonic anhydride bonds of SML-8-73-1 and SM-10-70-1. XY-02-075 is expected to be a promising compound despite 40 folds reduction in affinity compared to SML-8-73-1.^92^

As KRAS^G12C^ is the most researched mutation subtype, targeting some other mutations of KRAS that do not produce cysteine residues remains a challenge. Fortunately, it was found that nucleophilic residues other than cysteine could be selectively targeted by appropriately introducing covalent warheads. In 2022, Shokat and colleagues reported the development of covalent inhibitors of KRAS^G12S^ mutants and KRAS^G12R^ mutants.^93,94^ Using adagrasib as the parent core, they introduced a, β-lactone structure that can covalently target serine and successfully developed the first selective covalent inhibitor G12Si-5 (**24**) targeting KRAS^G12S^ mutants. G12Si-5 binds to the S-II P domain and inhibits oncogenic signaling, reducing ERK phosphorylation in KRAS^G12S^ mutant cells. The IC_50_ value of G12Si-5 in A549 cell line was 2.4 μM.^93^ Similarly, they successfully developed KRAS^G12R^ covalent inhibitors, G12R inhibitor-4 (**25**), by introducing α, β-diketoamide structures that covalently target arginine. The irreversible reaction of G12R inhibitor-4 combined with mutant arginine residues in S-II P was revealed by X-ray crystal structure, which showed imidazole condensation products formed between the α, β-diketoamide ligand and ε-, η- nitrogen of Arg12. Although arginine residues are less nucleophilic, they can be selectively targeted by small, electronphilic molecular reagents, providing the basis for the development of mutant-specific therapies against KRAS^G12R^-driven cancers.^94^

In addition, various KRAS^G12C^ covalent inhibitors with good clinical application prospects can be further obtained through structural optimization. For example, 1_AM (**26**), 2_AM (**27**), 3_AM (**28**), 4_AM (**29**), Fell series compound (**30–33**), Lanman series compound (**34–36**), Shin series compound (**37–40**).^95–98^ In conclusion, there is still great potential to obtain new covalent KRAS inhibitors through structural optimization. Some representative cases of KRAS^G12C^ covalent inhibitors in preclinical research are listed in (Table 1).

### Covalent EGFR inhibitors

Epidermal growth factor receptor (EGFR), a member of the receptor tyrosine kinase family, is a typical transmembrane receptor that initiates signaling cascades upon ligand-stimulated dimerization, thereby activating its tyrosine kinase and multiple downstream effectors.^99–101^ Moreover, it is involved in embryogenesis and stem cell division,^102^ and is implicated in cell proliferation, mitosis, and cancer development.^99,103,104^ Overexpression or increased activity of wild-type EGFR protein can lead to cell proliferation, migration, survival, and anti-apoptosis through signaling cascades, which are strongly associated with the occurrence and development of many cancers, such as NSCLC, breast cancer, glioma, head and neck cancer, cervical cancer, and bladder cancer.^105–108^ Therefore, EGFR has become a promising target for the design and development of anticancer drugs.

Targeted drugs for EGFR are tyrosine kinase inhibitors (TKIs), which inhibit the kinases in the cytoplasm, thus preventing them from activating the EGFR signaling pathway. First-generation EGFR TKIs, such as gefitinib and erlotinib, selectively bind to ATP-binding sites of EGFR tyrosine kinase with non-covalent bond, thereby inhibiting EGFR phosphorylation and significantly delaying disease progression in targeted therapy for NSCLC in the clinic. However, resistance gradually emerged: only 10-19% of patients with advanced non-small cell carcinoma experienced a tumor response to gefitinib;^109,110^ after using first-generation EGFR TKIs for approximately 9–14 months, almost all tumors progressed again.^111^ Afterwards, studies revealed that the reduced sensitivity to gefitinib or erlotinib in NSCLC was linked to EGFR-specific activating mutations.^112–116^

Mutated EGFR has developed resistance mechanisms to reversible inhibitors, thus limiting drug efficacy and rendering it undruggable. To overcome this problem, irreversible EGFR TKIs, namely second-generation EGFR TKIs, have been designed to covalently bind to the binding site, thus enhancing lasting inhibition of tumor cells. Compared to the first-generation EGFR TKIs, the second-generation EGFR TKIs, such as afatinib, daconmitinib and neratinib, possess an acrylamide Michael receptor side chain that can irreversibly bind to Cys797 at the ATP binding site, showing stronger inhibition effect in clinical practice. However, second-generation EGFR inhibitors still cannot be used to treat patients who develop resistance mutations after first-generation EGFR inhibitors, and can also lead to resistance.^117^ These resistances are often associated with T790M mutations, resulting in the development of the third-generation EGFR TKIs,^118^ such as WZ 4002, osimertinib, and rociletinib, which were specifically designed for T790M mutants rather than WT-EGFR. including. The third-generation drug retains the acrylamide group and covalently binds to Cys797, but replaces the quinazoline portion of the first- and second-generation compounds with pyrimidine to promote selectivity for T790M, showing a higher affinity for T790M than WT-EGFR.^119^ Therefore, developing covalent EGFR inhibitors is highly attractive. Currently, there are several EGFR TKIs on the market, of which second-generation TIKs and third-generation TKIs are covalent inhibitors. Here, we present the development of covalent drugs for EGFR inhibition (Table 1).

#### Marketed covalent drugs for EGFR inhibition

Afatinib (Giotrit^TM^, BIBW-2992) (**45**) is the first covalent EGFR inhibitor approved by the FDA for lung cancer, and is a second-generation EGFR TKI, which was marketed in July 2013. Similar to the first-generation EGFR TKI, afatinib forms hydrogen bonds to the main chain of Met793 in the hinge region and interacts with hydrophobic regions. The furanyl group is exposed to the solvent, and the 3-chloro-4-fluorophenyl group is located near the “gatekeeper” residue.^50^ Clinical trials have demonstrated that afatinib performs better in terms of overall survival than chemotherapy for those with EGFR exon 19 deletion, and provides an overall longer period of effective treatment and good disease control compared to gefitinib, a first-generation EGFR inhibitor.^120–123^ In addition, afatinib is covalently bound to Cys805 of HER2 and is known as a pan-HER2 inhibitor. However, afatinib showed dose-dependent cytotoxicity by inhibiting WT-EGFR and led to resistance to gefitinib and erlotinib. Furthermore, EGFR exon 20 insertion (ex20ins), which was present in 9.1% of patients with EGFR-mutated NSCLC, was found to be insensitive to afatinib.^124^

Dacomitinib (Vizimpro^TM^, PF-299804) (**46**), also an irreversible second-generation EGFR TKI originally developed by Pfizer and co-developed by SFJ Pharmaceuticals in 2012, was approved by the FDA in 2018 for the treatment of metastatic NSCLC with exon 19 deletion and exon 21 replacement.^125–128^ Dacomitinib has similar binding properties to afatinib, forming hydrogen bonds with hinge residues and hydrophobic interactions with those in the binding pocket.^129^ In addition, dacomitinib was found to be more promising for progression-free survival compared to gefitinib in a randomized, phase III clinical trial (ARCHER 1050); however, more severe adverse reactions were observed.^130^ As a first-line agent in EGFR mutation-sensitive NSCLC, dacomitinib has been demonstrated by many clinical trials to extend overall survival and show significant advantages over first-generation EGFR TKIs.^127,130^ As a result, the FDA approved dacomitinib in September 2018 for first-line treatment of advanced NSCLC.

Osimertinib (AZD9291) (**47**), a third-generation irreversible EGFR TKI currently approved for clinical use, received accelerated FDA approval in November 2015 for second-line treatment of NSCLC, and, subsequently, in 2018, FDA approval for first-line treatment. Based on the pyrimidine ring, osimertinib targets the Cys797 residue at the ATP binding site by forming covalent bonds through unsaturated allyl chains, thus irreversibly binding to the catalytic active center of EGFR kinase and inhibiting the phosphorylation of EGFR and its downstream signaling substrates Akt and Erk.^131^ Preliminary clinical studies have shown that osimertinib is capable of inhibiting the L858R mutant of EGFR up to 12 nM, and the IC_50_ of L858R/T790M mutant was 1 nM. The inhibition rate of osimertinib against EGFR^L858R/T790M^ mutant was approximately 200-fold higher than that of the wild type.^132^ Compared to the standard treatments of erlotinib or gefitin, osimertinib has demonstrated significant benefits in terms of both median progression-free survival and median duration of response in NSCLC patients with EGFR exon deletion 19 or L858R mutations.^133^ In addition, multiple studies have demonstrated that osimertinib is able to effectively penetrate the blood-brain barrier,^134,135^ providing a good therapeutic effect on BMS in advanced NSCLC, and significantly extending progression-free survival in cases of central nervous system (CNS) metastases.^136,137^ In addition to being used alone, osimertinib is also being studied in combination with other targeted therapies for NSCLC, such as inhibitors of the Met, Bcl-2, and MAPK pathways.^138–140^ Due to the significant efficacy of osimertinib, contemporaneous to the clinical development of osimertinib, several third-generation EGFR TKIs based on osimertinib structures were also being developed, some of which are also approved.

Aumolertinib (Almonertinib, HS-10296) (**48**) is an oral, irreversible third-generation EGFR TKI developed by Hansoh Pharmaceuticals. Structurally optimized from osimertinib, aumolertinib introduces a cyclopropyl, which can form hydrophobic interactions with Met790 side chains, to replace the methyl group on the indole ring. This optimization improves inhibitory activity and WT-EGFR selectivity, while simultaneously increasing lipophilicity and blood-brain barrier permeability.^141^ It was approved in China in March 2020 and was demonstrated to be a well-tolerated third-generation EGFR TKI, which can be used as a first-line treatment option for EGFR mutated NSCLC, in a phase III trial (NCT03849768) in 2022. However, aumolertinib is still more selective towards mutant EGFR, with semi-inhibitory concentration values for resistant or sensitized EGFR being approximately 2-16 times lower than those for wild-type enzymes.

Lazertinib (YH-25448) (**49**), an irreversible third-generation EGFR TKI developed by Genosco with strong blood-brain barrier penetration, has been shown to induced dose-dependent regression of subcutaneous and intracranial lesions in mice mutated with EGFR^L858R+T790M^. It has been shown to have superior efficacy in suppressing tumor growth and improving overall survival compared to the same dose of osimertinib, although adverse reactions were observed. The most common adverse reactions observed were pruritus (12%), decreased appetite (11%), rash (11%), and constipation (10%). The proportion of grade III or higher adverse reactions was 5%. A positive correlation between drug exposure and dose was observed, and no dose-limiting toxicity.^142–145^ In January 2021, it received marketing approval in South Korea for the treatment of NSCLC.

Alflutinib (Furmonertinib) (**50**) is a third-generation drug that specifically targets EGFR mutations and was independently developed in China. It is an optimized version of osimertinib, with a few key structural changes. Alflutinib incorporates 2,2,2-trifluoroethyl to replace methyl and introduces an N atom to replace the benzene ring with a pyridine ring. The retained Michael addition acceptor-acrylamide structure allows alflutinib to covalently bind to Cys797 residues, resulting in potent anti-tumor effects. Furthermore, alflutinib’s aminopyrimidine master loop can overcome steric hindrance caused by T790M mutation, while the introduction of the trifluoroethoxy-pyridine structure blocks the production of non-selective metabolites. This enhances alflutinib’s activity and kinase selectivity while reducing off-target effects, leading to fewer side effects.^146–148^ Alflutinib has high selectivity and strong tumor-shrinking properties, with minimal inhibitory effects on wild-type EGFR. It has been approved in China for treating locally advanced or metastatic NSCLC with EGFR-sensitive mutations since March 2021. In June 2022, it gained first-line indications for EGFR exon 19 deletion (Del19) or exon 21 (L858R) advanced NSCLC, with comparable efficacy to osimertinib. Overall, alflutinib’s unique design and effectiveness make it a promising therapeutic option for EGFR-mutated NSCLC patients.^149^

Mobocertinib (TAK-788) (**51**) is a novel oral targeted EGFR/HER2 drug, belonging to the fourth-generation of EGFR inhibitors. Structurally similar to osimertinib, it possesses an enhanced inhibitory effect against EGFR exon 20 insertion and other non-sensitive mutations, as well as some inhibitory effect against lung cancer with HER2 exon 20 insertion mutations.^150,151^ In September 2021, the FDA approved mobocertinib for metastatic NSCLC, advanced NSCLC with EGFR mutation, locally advanced NSCLC, and advanced NSCLC. The drug also received marketing approval in China in January 2023.

Olmutinib (HM61713. BI-1482694) (**52**) is an orally effective small molecule with potential antitumor activity as a mutation-selective third-generation EGFR inhibitor developed by Hanmi Pharmaceutical Co Ltd for the treatment of NSCLC and lung adenocarcinoma. It binds to cysteine residues near the kinase domain, thereby inducing cell death in tumor cells expressing EGFR.^152,153^ In May 2016, it was approved for marketing in Korea for the treatment of patients with locally advanced or metastatic NSCLC that is positive for the EGFR^T790M^ mutation. However, as reported by the Korea Ministry of Food and Drug Safety (MFDS) on September 30, 2016, olmutinib resulted in the death of two patients due to severe skin and mucous membrane necrosis during clinical trials, and it has issued a prescribing caution warning against the use of olmutinib in new patients. Following the safety incident, the Korean MFDS issued a statement saying that the adverse event had not been reported in previous clinical trials and that while the clinical use of olmutinib has not been suspended, the Korean approach has recommended that patients who need to use the drug should use it cautiously at the discretion of their doctors.

Neratinib (**53**) is an oral, potent and irreversible third-generation EGFR TKI that inhibits tumor growth and metastasis by blocking the pan-HER family (HER1, HER2, and HER4) and downstream signaling pathway transduction. This drug is originally developed by Wyeth (now Pfizer) and then Puma Biotechnology. Not only does it competitively occupy the ATP-binding site on EGFR, but it also binds to the unique amino acid residue Cys805 near the opening of the pocket-a homologous cysteine residue to EGFR Cys797-to undergo alkylation or covalent bonding, thus achieving irreversible inhibition of HER2.^154,155^ Neratinib was approved by the FDA in July 2017 for the treatment of breast cancer, making it the only product in the world approved for intensive adjuvant therapy with trastuzumab (herceptin) in HER2-positive breast cancer to reduce the risk of recurrence.

Pyrotinib (SR-1258) (**54**), developed by Jiangsu Hengrui Medicine Co Ltd, is an effective, selective and irreversible HER2/EGFR dual-target tyrosine kinase inhibitor with IC_50_ values of 38 and 13 nM, respectively. Similarly, as the third-generation EGFR TKI, pyrotinib covalently binds to ATP binding sites in intracellular kinase regions of EGFR, HER2 and HER4, preventing homodimer formation, thereby irreversibly inhibiting autophosphorylation, blocking activation of downstream signaling pathways, and inhibiting tumor cell growth.^156–158^ It received conditional marketing approval from the National Medical Products Administration (NMPA) in August 2018.

#### Covalent EGFR inhibitors being applied for approval

At present, several drugs are in the marketing application stage, such as avitinib, oritinib, sunvozertinib, and rezivertinib. All of these drugs are structurally derived from the third-generation EGFR TKI Osimertinib, which is already available in the market. Avitinib (Abivertinib, AC0010) (**55**) is a third-generation, irreversible, mutant-selective EGFR inhibitor. Avitinib forms a covalent bond to C797 in the ATP-binding pocket and has potential antitumor activity. Avitinib inhibits the phosphorylation of EGFR^Y1068^ and its downstream molecule Akt and extracellular signal-regulated kinase (ERK1/2) in H1975 and HCC827 cells. Moreover, the IC_50_ for EGFR^L858R/T790M^ double mutant was 0.18 nM.^159,160^ Avitinib inhibits cell proliferation, reduces colony formation, and induces apoptosis and cell cycle arrest in ACUTE MYELOGENOUS LEUKEMIA cells, especially those carrying FLT3-ITD mutations. Avitinib is also a novel BTK inhibitor.^161,162^

Oritinib (SH-1028) (**56**) is an irreversible, selective third-generation EGFR TKI. Oritinib overcomes T790M-mediated drug resistance in NSCLC and inhibits WT-EGFR, EGFR^L858R^, EGFR^L861Q^, EGFR^L858R/T790M^, EGFR^d746-750^, and EGFR^d746-750/T790M^ kinases. IC_50_ were 18, 0.7, 4, 0.1, 1.4 and 0.89 nM, respectively. Oritinib binds irreversibly to EGFR kinase by covalently bonding to form Cys797 residues targeting ATP binding sites. Oritinib effectively and selectively targets mutated EGFR cell lines in vitro.^142,163–165^

Sunvozertinib (DZ-0586, DZD-9008) (**57**) is an oral, highly effective and irreversible selective EGFR TKI independently developed by Dizal Pharm Co Ltd. It is the world’s first small molecule compound designed for EGFR/HER2 exon 20 insertion mutation. It has strong activity against a variety of EGFR mutations including EGFR exon 20 insertion mutations and HER2 exon 20 insertion mutations. Sunvozertinib shows strong antitumor activity in cell lines and xenograft models. Additionally, as an oral agent, sunvozertinib demonstrates desirable drug metabolism and pharmacokinetic (DMPK) characteristics in both preclinical and clinical settings.^166^ In January 2022, sunvozertinib was granted breakthrough therapy designation by the FDA for the treatment of adult patients with locally advanced or metastatic NSCLC whose disease has progressed during or after prior platinum-containing chemotherapy and who have tested positive for EGFR exon 20 insertion mutations. In September of the same year, Dizal Pharm Co Ltd announced the results of the Chinese registered clinical trial of sunvozertinib in the treatment of EGFR exon 20 insertion (Exon20ins) mutant advanced NSCLC at the European Society of Internal Oncology (ESMO) Congress. The confirmed tumor response rate (ORR) assessed by the Blind Independent Center Evaluation Committee (BICR) was 59.8%, and the registered clinical trial met its primary endpoint. In the follow-up, the company still needs to complete the communication with CDE, submit the new drug marketing application, complete the technical review, on-site verification and other procedures.^167,168^

Rezivertinib (BPI-7711) (**58**) is an orally effective, highly selective and irreversible third-generation EGFR TKI. Rezivertinib shows highly selective inhibitory effects on EGFR^Del E746-A750^, EGFR^T790M^, EGFR^L858R/T790M^ double mutations, including EGFR single mutations, but shows a weak inhibitory effect on WT-EGFR. Rezivertinib has excellent central nervous system (CNS) penetration and antitumor activity. Rezivertinib selectively inhibits the proliferation of EGFR mutated cells in cell lines.^142,169–171^

#### Covalent EGFR inhibitors in clinical trials

Currently, several covalent EGFR inhibitors are undergoing clinical trials, all of which are third-generation or more advanced EGFR TKIs. These inhibitors have demonstrated promising efficacy in inhibiting EGFR mutations. Olafertinib (CK-101/RX518) (**59**) is an oral selective EGFR covalent inhibitor approved for second-line treatment in patients with EGFR^T790M^ mutation NSCLC and first-line treatment in patients with EGFR sensitive mutation (Del19, L858R) NSCLC.^172^ The drug also shows promise in combination therapy with immune checkpoint inhibitors (PD-1 or PD-L1), c-Met inhibitors, and Mek inhibitors, as demonstrated by preclinical studies. In October 2016, a clinical trial (NCT02926768) began to evaluate the phase I/II study of olafertinib in patients with NSCLC and other advanced solid tumors. In September 2017, the FDA granted Checkpoint Therapeutics orphan drug status for olafertinib in patients with EGFR mutation-positive NSCLC. In 2021, a phase I clinical study (CTR20182402) assessing the safety, tolerability, pharmacokinetics, and initial efficacy of olafertinib in patients with advanced NSCLC was completed, but the results have not been published. In June 2022, the phase I/II study (NCT02926768) was concluded, but the results have not been published yet. A phase III clinical study (CTR20200563) investigating the efficacy and safety of olafertinib in first-line treatment of locally advanced or metastatic NSCLC patients with EGFR mutations is still ongoing.

Nazartinib (EGF816, NVS-816) (**60**) is a third-generation, covalent, irreversible, and highly selective inhibitor of mutant EGFR, developed by Novartis.^173^ This drug specifically targets and inhibits the activity of mutant forms of EGFR, thus preventing EGFR-mediated signal transduction. Nazartinib has been shown to exhibit nanomolar level inhibition of mutant EGFR (L858R, Ex19del) and T790M, demonstrating superior specificity towards mutant EGFR as compared to WT-EGFR. Additionally, it exhibits excellent ADME (Absorption, Distribution, Metabolism, Excretion) and PK (Pharmacokinetics) properties. The drug demonstrates potent inhibitory effects on pEGFR levels in H3255, HCC827, and H1975 cell lines, leading to effective inhibition of cell proliferation.^173–176^ Although the sponsor withdrew the study of nazartinib and erlotinib/gefitinib in the first-line treatment of locally advanced/metastatic NSCLC with EGFR mutations (NCT03529084) in 2019. The latest study shows that nazartinib continues to be studied as a combination drug in a clinical trial (NCT03040973), which called “Study to allow patients previously participating in a Novartis sponsored trial to continue receiving capmatinib treatment as single agent or in combination with other treatments or the combination therapy alone”.

Allitinib (AST-1306) (**61**) is an orally available anilino-quinazoline compound with demonstrated anticancer activity. It irreversibly inhibits EGFR with an IC_50_ value of 0.5 nM, and also inhibits ErbB2 and ErbB4 with IC_50_ values of 3 and 0.8 nM, respectively. In HIH3T3-EGFR T790M/L858R cells, allitinib significantly and dose-dependently inhibited cell growth (0.19–6.25 μM; 72 h). It also inhibited the activation of tyrosine kinase and downstream signaling pathways in A549 cells, Calu-3 cells, and SK-OV-3 cells. In A549 cells, allitinib (0.001–1.0 μM; 4 h) showed 3000-fold selectivity to ErbB family kinases over other kinase families, and dose-dependently inhibited EGF-induced EGFR phosphorylation. Allitinib effectively inhibits the EGFR^T790M/L858R^ double mutant with an IC_50_ value of 12 nM.^177–181^ Although enrolled in a phase II clinical trial (NCT04671303) in December 2020 to evaluate the efficacy and safety of combined treatment with anlotinib in lung cancer, allitinib has not yet been administered.

ES-072 (**62**) is a promising new generation of EGFR inhibitor, independently developed by Zhejiang Bossan Pharmaceutical Co Ltd, that is superior to the third-generation EGFR inhibitors. It is specifically designed to inhibit EGFR^L858R/Del19^ and EGFR^T790M^, while also addressing resistance acquired from first-generation EGFR inhibitors without T790M variants, as well as those from third-generation EGFR inhibitors. Notably, preclinical data indicates that ES-072 has the ability to penetrate the blood-brain barrier, making it a potentially effective treatment for brain metastases. In January 2018, ES-072 was registered for a phase I clinical trial (CTR20180074) to assess its efficacy in NSCLC patients with EGFR mutations.^182^ This single-center, open, dose-escalation trial aims to evaluate the safety and tolerability of ES-072 in patients with locally advanced or metastatic NSCLC. Additionally, Bossan Pharmaceutical Co Ltd has collaborated with CBT Pharmaceuticals to develop combination therapies involving ES-072 and c-Met inhibitors, as well as PD-1 antibodies. Several clinical trial applications related to ES-072 have been accepted in recent years (CXHL1700078, CXHL1700080, CXHL1700079), and clinical trial approval documents have been obtained, highlighting the growing interest and potential of this promising drug.^183^

YK-029A (**63**), an oral, irreversible third-generation EGFR TKI, is another osimertinib analog developed by Hainan Yuekang Biopharmaceutical Co Ltd. The drug is intended to treat advanced NSCLC with drug resistance and disease progression acquired by T790M gene mutation after previous treatment of EGFR TKIs. YK-029A has shown promise in preclinical studies, leading to its registration for a clinical phase I trial (CTR20180350) in May 2018. Furthermore, several clinical trial applications related to YK-029A have been accepted (CXHL2200062, CXHL2101515, CXHL1700173, CXHL1700174), and clinical trial approval documents have been obtained in recent years. These developments suggest growing interest in and potential for YK-029A as a treatment option for patients with advanced NSCLC.^184,185^

#### Covalent EGFR inhibitors in terminated clinical trials

With the emergence of new covalent EGFR inhibitors entering clinical trials, some clinical trials involving EGFR inhibitors have been terminated for various reasons. Canertinib (CI-1033; PD-183805) (**64**) is an irreversible inhibitor of the EGFR that effectively inhibits cellular EGFR and ErbB2 autophosphorylation with IC_50_s of 7.4 and 9 nM, respectively. In cultured melanoma cells (RaH3 and RaH5), canertinib significantly inhibits their growth in a dose-dependent manner, leading to G1-phase cell cycle arrest without inducing apoptosis. Notably, 1 μM canertinib also inhibits ErbB1-3 receptor phosphorylation and decreases Akt-, ERK1/2-, and Stat3 activity in both cell lines.^186–189^ Canertinib was enrolled in clinical trials in December 2002, and completed studies investigating its efficacy in combination with paclitaxel/carboplatin for the first-line treatment of NSCLC (NCT00174356), as well as in patients with metastatic (stage IV) breast cancer (NCT00051051) and as a single agent for the treatment of advanced NSCLC (NCT00050830) between 2002 and 2007. However, no further follow-up on canertinib has been reported since. Additionally, canertinib has shown potential as a treatment against vaccinia virus respiratory infection in mice.

Rociletinib (Xegafri^TM^, CO-1686) (**65**), developed by Clovis Oncology, is a specific mutant agent for the treatment of NSCLC, belonging to the third-generation EGFR TKIs.^190,191^ In the EGFR^T790M^, the anilinopyrimidine group in rociletinib forms two hydrogen bonds with Met793 amide and carbonyl backbone, which became a hydrophobic interaction in the T790M structure. Rociletinib was also able to form two hydrogen bonds in EGFR^L858R^. These include one between nitrogens in the pyrimidine group, and another between the fluoromethyl and Thr790. In both active (DFG-in/αC-in) conformations, the acrylamide group in rociletinib covalently binds to Cys797.^192^ However, it induced various adverse reactions in clinical trials, including nausea (35%), fatigue (24%), diarrhea (22%), prolonged QT interval (22%) and hyperglycemia (22%). Hyperglycemia was mainly a tertiary adverse event, but it could be controlled by tapering or oral metformin. Despite this, at the April 2016 ODAC meeting, experts voted to delay approval of rociletinib, and Clovis announced the termination of rociletinib.^193^

Naquotinib (ASP8273) (**66**) is an orally available, irreversible and mutant-selective EGFR^L858R/T790M^ inhibitor that has shown potential as an antitumor agent. It covalently bound to an EGFR mutant (L858R/T790M) through cysteine residues to chronically inhibit phosphorylation of EGFR. Naquotinib also inhibits signaling pathways through ERK and Akt, and is active against EGFR mutant cell lines resistant to other EGFR TKIs such as AZD9291 and CO-1686.^194,195^ In May 2017, Astellas announced the termination of a phase III clinical study (NCT02588261) of naquotinib in NSCLC due to a recommendation from the Independent Data Monitoring Committee (IDMC). Subsequently, as a result, clinical trials of naquotinib have stopped recruiting patients altogether.

Mavelertinib (PF-06747775) (**67**) is an orally available and irreversible EGFR TKI that selectively targets various EGFR mutants, such as Del, L858R, T790M/L858R and T790M/Del, with less than 50% effect or inhibition against all nonkinase targets.^196–198^ In May 2015, a clinical study (NCT02349633) was initiated to investigate mavelertinib in patients with NSCLC EGFR mutation (Del 19 or L858R +/− T790M). However, due to strategic reasons and changes in the external environment, the study was eventually discontinued in June 2021 when results were updated.

#### Covalent EGFR inhibitors in preclinical research

In addition, several covalent EGFR inhibitors are still in preclinical development. CL-387785 (EKI-785, WAY-EKI 785) (**68**) is a highly selective and irreversible EGFR inhibitor that specifically inhibits kinase activity of the protein (IC_50_ = 370 pM). It effectively blocks autophosphorylation of receptors in EGF-stimulated cells (IC_50_ approximately 5 nM) and inhibits cell proliferation in a cytostatic manner mainly in cell lines overexpressing EGFR or c-ErbB-2 (IC_50_ = 31–125 nM). While most EGFR mutants transform cells and make them sensitive to erlotinib and gefitinib, the exon 20 insertion transformation confers resistance to these inhibitors but makes the cells more sensitive to the irreversible inhibitor, CL-387785. CL-387785 has also shown potential to overcome T790M mutation-related resistance at the functional level, possibly by effectively inhibiting downstream signaling pathways.^199–202^ Despite its promising profile, no recent reports are available on CL-387785, as it remains under clinical development.

Currently, there are also various lead compounds being developed as covalent EGFR inhibitors and antitumor agents. In 2009, Pasi et al. identified a series of covalent pyrimidine EGFR inhibitors, including WZ 3146, WZ 4002, and WZ 8040, through screening a library of irreversible kinase inhibitors specific to EGFR^T790M^.^203^ These compounds showed a desirable 300-fold lower IC_50_ against the PC9GR cells compared to clinical-stage inhibitors such as HKI-272. In vitro, they were 30-100 folds more effective against EGFR^T790M^ and up to 100-fold less effective against WT-EGFR than quinazoline based EGFR inhibitors. Additionally, they have demonstrated efficacy in murine models of lung cancer driven by EGFR^T790M^. Among them, WZ 4002 (**69**) exhibited the highest efficacy and effectively inhibited the phosphorylation of EGFR, Akt, and ERK1/2. Some presentive promising molecules are listed in (Table 1).

Four synthesized derivatives of 6- or 7-acrylamide-4-anilino-quinazolines, PD 160678 (**70**), PD 168393 (**71**), PD 160879 (**72**), PD 174265 (**73**), irreversibly inhibit EGFR TK activity with IC_50_ values 0.45–0.70 nM.^204^

### Covalent p53 modulators

P53 is a crucial protein that regulates the cell cycle and acts as a tumor suppressor.^205^ Studies have shown that approximately half of all human cancers, including serous ovarian cancer, lung squamous cell cancer, lung small cell cancer, triple-negative breast cancer, and squamous esophageal cancer, have alterations in the p53 gene, resulting in a loss of p53 function or decreased p53 expression.^206–208^ As a tumor suppressor TFs closely linked to PPIs, p53 plays a critical role in regulating gene expression, promoting tumor cell cycle arrest, apoptosis, and DNA repair. It can activate nearby or distant genes in response to an enhancer, while also indirectly inhibiting the transcription of numerous genes.^209–213^ P53 can be categorized as mutant type or wild type, with mutant p53 promoting tumorigenesis and wild-type p53 having broad-spectrum tumor inhibition.^206–208^ TP53 mutations typically reduce the expression of p53 protein or produce inactive variants, thus compromising its cancer-inhibiting properties. As a result, therapeutic strategies are needed to restore p53 function. However, most small molecules target overexpressed proteins by inhibiting their activity, making p53 an “undruggable” target.

#### Covalent modulators directly targeting p53

In 2022, Kevan M. Shokat’s team continued their research and development work on the KRAS^G12S^ mutant and developed a small molecule covalent inhibitor of p53-Y220C mutant, known as KG13 (**74**) (Table 1).^214^ This inhibitor is specifically designed to bind to the p53 Y220C mutant, which restores the thermal stability of p53 protein to the level of wild-type p53 protein and activates the expression of downstream genes. The researchers designed 13 small molecule drugs to target the pocket structure formed by p53 Y220C in space. After a series of structural modifications and screening, KG13 was selected as the best small molecule compound with the highest covalent labeling rate and thermal stability recovery rate. Additionally, cells treated with KG13 demonstrated p53 Y220C-dependent p53 target gene activation, inhibition of cell growth, and increased caspase activity.

#### Covalent p53-MDM2 PPI inhibitors

Both Murine double minute 2 (MDM2, HMD2 in human) and MDMX act as negative regulators of p53, maintaining p53 at a low level by directly binding to its N-terminal and mediating its degradation in normal cells.^215^ The primary mechanism of p53 degradation involves ubiquitylation by the E3 ubiquitin ligase MDM2, which leads to proteasomal degradation of p53. MDM2 amplification is frequently observed in several cancer types, especially in tumors that still retain wild-type p53.^216,217^ Since MDM2-mediated ubiquitylation and degradation depend on its direct interaction with p53, researchers have been searching for small molecules that can inhibit this interaction to stabilize p53 and restore its activity. Although most p53-MDM2 inhibitors are non-covalent (which will be explained in the PPI inhibition part), some small molecule inhibitors that target p53-MDM2 have been found to be covalent, leading to the development of covalent p53-MDM2 inhibitors (Table 1).

In 2017, Ishiba et al. performed mirror-image screening through _D_-proteins, which is an approach for identifying potential pharmaceutical candidates from homochiral resources, and revealed that NPD6878 (apomorphine) (**75**) was an MDM2–p53 inhibitor candidate with high potency.^218^ At equipotent doses, R-(-)-apomorphine inhibited both the native _L_-MDM2-_L_-p53 interaction (IC_50_ = 0.215 μM) and the mirror-image _D_-MDM2-_D_-p53 interaction (IC_50_ = 0.195 μM). In addition, the enantiomer, S-(-)-apomorphine also showed equipotent inhibitory activity against the _L_-MDM2-_L_-p53 interaction (IC_50_ = 0.175 μM). Among these, the achiral oxoapomorphine, which was converted from chiral apomorphine under aerobic conditions, served as the reactive species to form a covalent bond at Cys77 of MDM2 with Michael acceptors, leading to the inhibitory effect against the binding to p53.^219^

In 2021, Hamachi et al. developed a small molecule covalent inhibitor, compound (**76**), based on the *N*-acyl-*N*-alkyl sulfonamide (NASA) reaction group, which can prevent the interaction between HDM2 and p53.^220,221^ The researchers used a reactive NASA group as the warhead and conducted quality-based analysis to reveal the kinetics of covalent inhibition. They identified that the modification sites on HDM2 were the N-terminal alpha-amine and Tyr67. Using Nutlin-3 as a scaffold for a covalent inhibitor, the researchers found that Lys51, which an *N*‑acyl‑*N*‑alkyl sulfonamide (NASA) warhead could target, was about 11 Å away from the 2-oxypiazine portion of Nutlin-3a.^222,223^ They then structurally modified to generate a series of covalent compounds, which were tested for their ability to modify HDM2 in vitro. Through in vitro studies, this compound was found to exhibit severe p53-independent cytotoxicity, leading to its selection among the compounds. Additionally, it demonstrated a longer residence time on HDM2 compared to the non-covalent inhibitor Nutlin-3, resulting in higher HDM2/p53 inhibitory potency under diluted conditions. This compound was determined to selectively inhibit HDM2-induced p53 pathologically dependent apoptosis, instead of causing non-specific cytotoxicity caused by NASA warheads. This study marks a significant advancement in the rational design of effective covalent PPI inhibitors.

### Other covalent inhibitors

#### Covalent Mcl-1 inhibitor

Myeloid cell leukemia-1 (Mcl-1) is a crucial anti-apoptotic member of the Bcl-2 protein family that contributes significantly to the development of various human cancers. Targeting the BH3 binding groove of Mcl-1 has emerged as a promising approach for inhibiting its function and has become a focal point in the development of antitumor drugs.^224–227^ In this regard, Lee et al. devised a drug design strategy based on a table of variable texture sites near the BH3 region opposite to the binding site. They utilized the covalent inhibitor, MAIM1 (77) (Table 1), which combines with Cys286 of the thalproquinone type, to effectively inhibit Mcl-1 activity (IC_50_ = 450 nm).^228^ This compound tightly binds to Mcl-1 and provides potential new pathways and drug precursor compounds for anti-apoptotic tumor therapy. Structural and functional analyses showed that the BH3 binding force and its inhibition of Bax were impaired by molecular bonding, as observed in the C286W mutagenesis simulation in vitro and in cells. This study offers valuable insights into the development of novel Mcl-1 inhibitors for cancer therapy.

#### Covalent PKM2 inhibitor

In a recent study on targeted covalent inhibitors, the Cross Center of Shanghai Institute of Organic Sciences and collaborators reported a novel PKM2 inhibitor, compound (**78**) (Table 1), based on trivalent arsine covalent warheads.^229^ Although arsenic compounds are known for their toxicity and have been abandoned in modern medicine, a variety of organic arsine drugs targeting tumor delivery have been introduced into the clinic with success.^230–233^ The trivalent arsine functional group has potential as a covalent warhead due to its ability to react with cysteine residues in proteins, affecting their activity and exerting a drug effect. Using organic arsenic covalent probes, chemical proteomics, and pharmacochemical methods, a highly active and specific covalent PKM2 inhibitor was developed.^229^ The compound effectively inhibited ovarian cancer growth in vivo with IC_50_s value of 0.16 and 0.23 μM in PA-1 and A2780 cells, respectively. Treatment with this compound reduced tumor load in mice via gavage at 50 mg kg^−1^ day^−1^. Its derivative compound formed a covalent bond with Cys474 near the allosteric activation pocket of PKM2, specifically inhibiting its activity without affecting PKM1. The study suggests that organic arsine compounds have potential as targeted covalent inhibitors, offering a broader application prospect in precision cancer therapy.

## Allosteric modulators

The initial focus of rational drug design was on the orthosteric sites of therapeutic protein targets.^234,235^ However, many of these targets have been found to be undruggable or difficult to target in their orthosteric sites due to their high affinity with substrates, lack of structural information, or high conservation of active sites. To overcome these challenges, allosteric regulation has been proposed as a strategy commonly used in nature to control cellular processes by modulating the affinity of biomolecules “at a distance”. Allosteric modulators can change the protein/substrate affinity in a highly predictable manner by stabilizing target proteins in an inactive or active state, leading to desirable controllability.^236–241^

Allosteric modulators offer several advantages over orthosteric inhibitors. Firstly, allosteric ligands do not have to compete with high-affinity substrates, making it simpler to develop allosteric modulators.^238,242,243^ Secondly, allosteric sites are diverse and confer better selectivity among homologous proteins, resulting in fewer side effects and greater value in clinical applications.^244,245^ Thirdly, allosteric modulators have a desirable “ceiling of effect”. Once allosteric sites are occupied, no additional effects can be observed, indicating drug safety under overdose conditions.^246^ Additionally, undruggable proteins can be targeted simultaneously by orthosteric inhibitors and allosteric modulators to achieve a synergistic effect and overcome resistance.

Allosteric modulators can not only inhibit targets like orthosteric inhibitors but can also stabilize them or competitively occupy them if needed to improve pathological states.^6,47,247^ According to their effects on the receptor, allosteric modulators can be classified into three categories: positive allosteric modulators (PAMs), which improve the action of orthosteric effectors but have no intrinsic activity; negative allosteric modulators (NAMs), which inhibit the function of orthosteric effectors; and silent allosteric modulators (SAMs), also known as neutral allosteric modulators, which inhibit allosteric activities by blocking the allosteric site of both PAMs and NAMs.^248,249^

Therefore, the identification of allosteric sites and corresponding drug design has opened up new therapeutic opportunities for proteins that were previously considered “undruggable” or “difficult to target” at their orthosteric site. Since the concept of allosteric modulation was first proposed in the 1960s, a number of allosteric drugs have been applied in clinical practice, evaluated at clinical trial phases or preclinical stages. Initially, allosteric drug design focused on inhibiting kinases and GPCRs with highly conserved active sites, as an alternative option to overcome the undesired selectivity profiles and resistance that occurs in the clinical application of orthosteric modulators.^250–252^ After a decade of development, the range of target categories has expanded to others, including several undruggable proteins that lack marketed drugs, such as KRAS and SHP2. It is noteworthy that some targets provide the opportunity for combined application of covalency and allostery in drug design. For instance, AMG510 (sotorasib), the first marketed KRAS inhibitor that was granted accelerated approval (Lumakras™, Amgen, Inc.) by the FDA, is a covalent allosteric inhibitor of KRAS^G12C^ mutant, highlighting the significance of rational design of covalent allosteric drugs.^55^

**Fig. 2:**
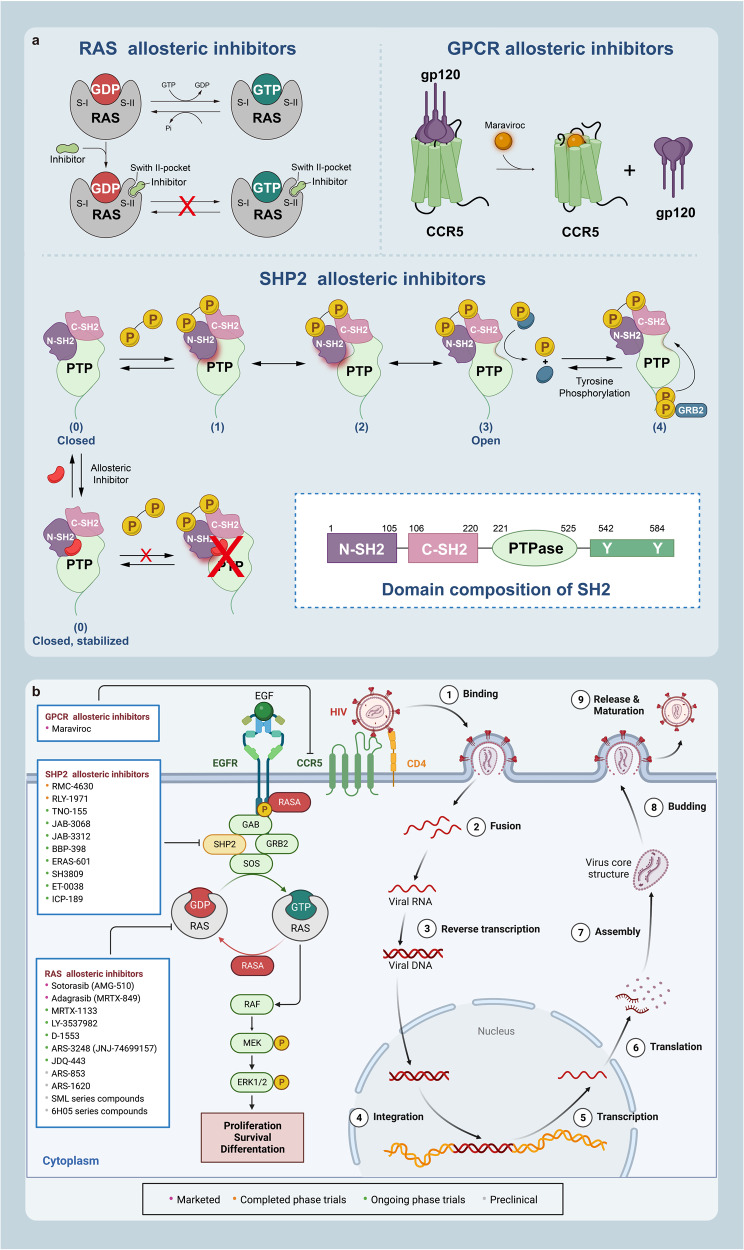
Allosteric inhibitors targeting undruggable proteins. Allosteric modulators change the protein/substrate affinity by stabilizing target proteins in an inactive or active state “at a distance”. **a** Binding mode of selected allosteric modulators: RAS allosteric inhibitors interact with mutant amino acids in switch II region to induce conformational changes, thereby locking KRAS in an inactive conformation; SHP2 allosteric inhibitors directly stabilize the autoinhibited conformation of SHP2, thereby preventing interactions between the catalytic PTP domain and SHP2 substrates; GPCR allosteric inhibitors can be classified into PAMs, allosteric antagonists and NAMs based on their mode of action. **b** Map of marketed, clinical and preclinical allosteric inhibitors in signaling pathways

In this part, we summarize the development in drug design and clinic trials targeting allosteric sites of undruggable proteins and those proteins which are hard to selectively target with orthosteric inhibitors (Table 2 and Fig. 2).

**Table 2:** Allosteric modulators targeting undruggable proteins

Compound name and structure	Target	Cancer cell line (activity)	Indications	Status/clinical trial identifier	Ref.
MRTX-1133 (**79**)	KRAS^G12D^	–	Colorectal cancer, NSCLC, pancreas cancer	*Ongoing* NCT05737706(I/II)	^254^
TNO-155 (**80**)	SHP2	–	Colorectal cancer, esophageal cancer, NSCLC, etc.	*Ongoing* NCT05541159(I), NCT05490030(I), NCT04000529(I), NCT03114319(I), NCT04330664(I/II)	^272^
JAB-3068 (**81**)^a^	SHP2	–	Esophagus cancer, NSCLC, etc.	*Ongoing* NCT04721223(I/II), NCT03565003(I/II), NCT03518554(NA)	^274^
RMC-4630 (**82**)	SHP2	–	Colorectal cancer, NSCLC	*Completed* NCT03989115(I/II) *Ongoing* NCT04916236(I), NCT03634982(I), NCT05054725(II)	^275^
JAB-3312 (**83**)^a^	SHP2	–	Colorectal cancer, esophagus tumor, NSCLC, etc.	*Ongoing* NCT05288205(I/II), NCT04720976(I/II), NCT04121286(I), NCT04045496(I)	^277^
RLY-1971 (**84**)^a^	SHP2	–	Advanced solid tumor	*Completed* NCT04252339(I)	^278^
BBP-398 (**85**)	SHP2	–	Metastatic NSCLC	*Ongoing* NCT05621525(I), NCT05480865(I), NCT05375084(I), NCT04528836(I)	^279^
ERAS-601 (**86**)^a^	SHP2	–	Acute myelogenous leukemia, NSCLC	*Ongoing* NCT04959981(I/II), NCT04866134(I/II), NCT04670679(I)	^280^
SH3809 (**87**)^a^	SHP2	–	Advanced solid tumor	*Ongoing* NCT04843033(I)	^281^
ET-0038 (**88**)^a^	SHP2	–	Advanced solid tumor	*Ongoing* NCT05354843(I), NCT05525559(I)	^282^
ICP-189 (**89**)^a^	SHP2	–	Advanced solid tumor	*Ongoing* NCT05370755(I)	^283^
SHP099 (**90**)	SHP2	Caco-2 (IC_50_ = 0.07 μM)	–	*Preclinical*	^286^
RMC-4550 (**91**)	SHP2	MIA PaCa-2, NCI-H35 (IC_50_ = 0.583 nM)	–	*Preclinical*	^289^
PCC0208023 (**92**)	SHP2	LS180, HCT116 (IC_50_ = 2.1 nM)	–	*Preclinical*	^287^
TK-453 (**93**)	SHP2	–	–	*Preclinical*	^293^
Cinacalcet (AMG-073) (**94**)	CaS	–	Hypercalcemia, hyperparathyroidism, SHPT	*Marketed*	^657^
Maraviroc (**95**)	CCR5	–	HIV infection	*Marketed*	^658^
Ticagrelor (AZD-6140) (**96**)	P2Y12	–	Arterial thrombosis, Ischemic stroke, etc.	*Marketed*	^659^
Avacopan (CCX168) (**97**)	C5a1	–	Vasculitis	*Marketed*	^660^
Vercirnon (**98**)	CCR9	–	Celiac disease, inflammatory bowel disease	*Completed* NCT01277666(III), NCT00102921(II), NCT01114607(I), etc. *Terminated* NCT01536418(III), NCT01318993(III), etc.	^661^
Mavoglurant (**99**)	mGlu5	–	Cocaine addiction, obsessive–compulsive disorder	*Completed* NCT02920892(II), etc. *Ongoing* NCT03327792(I); NCT05203965(0) *Withdrawn* NCT04771143(I) *Terminated* NCT01019473(II), etc.	^662^
T-62 (**100**)	A1	–	Neuropathic pain, postherpetic neuralgia	*Withdrawn* NCT00506610(II) *Terminated* NCT00809679(II)	^663^
AZD-8529 (**101**)^a^	mGlu2	–	Schizophrenia	*Completed* NCT02401022(II), NCT00921804(II), etc.	^664^
Raseglurant (ADX10059) (**102**)	mGlu5	–	Gastroesophageal reflux, migraine, Parkinson’s disease	*Completed* NCT00820079(II), NCT00810485(II) *Terminated* NCT00820105(II)	^665^
Basimglurant (RG-7090) (**103**)	mGlu5	–	Trigeminal neuralgia	*Completed* NCT02433093(I) *Ongoing* NCT05059327(II), NCT05217628(II)	^666^
JNJ-40411813 (**104**)	mGlu2	–	Epilepsy	*Completed* NCT01582815(II), NCT01323205(II), NCT04677530(I), etc. *Ongoing* NCT04836559(II)	^667^
(11 C) JNJ-42491293 (**105**)^a^	mGlu2	–	Psychiatric disorder	*Completed* NCT01359852(I)	^668^
MK-7622 (**106**)	M1	–	Alzheimer’s disease	*Terminated* NCT01852110(II)	^669^
RG-7342 (**107**)^a^	mGlu5	–	Schizophrenia	*Terminated* NCT02196636(I)	^670^
ODM-106 (**108**)^a^	GABAB	–	Essential tremor	*Completed* NCT02393950(I)	^671^
JNJ-55375515 (**109**)	mGlu2	–	Neurological disease, psychiatric disorder	*Completed* NCT03405441(I), NCT02623491(I)	^672^
MK-6884 (**110**)	M4	–	Alzheimer’s disease	*Completed* NCT02621606(I)	^673^
ASP-4345 (**111**)^a^	D1	–	Cognitive disorder	*Completed* NCT03557931(II), NCT02720263(I)	^674^
TAK-071 (**112**)	M1	–	Cognitive disorder, Parkinson’s disease	*Ongoing* NCT04334317(II) *Terminated* NCT02918266(I), NCT02769065(I)	^675^
Foliglurax (DT-1687) (**113**)	mGlu4	–	Parkinson’s disease	*Completed* NCT03162874(II), etc. *Withdrawn* NCT03331848(II) *Terminated* NCT04322227(I), etc.	^676^
ASP-8302 (**114**)^a^	M3	–	Urinary dysfunction	*Completed* NCT03702777(II), NCT03361540(I)	^677^
HTL0014242 (TMP-301) (**115**)	mGlu5	–	Neurological disease, psychiatric disorder	*Completed* NCT04462263(I), NCT03785054(I)	^678^
JNJ-2463 (nimacimab) (**116**)^a^	CB1	–	Diabetic gastroparesis	*Unknown status* NCT03900325(II)	^679^
RGH-618 (**117**)	mGlu5	–	Generalized anxiety disorder	*No progress*	^680^

Data collected from https://clinicaltrials.gov [last accessed March 2023]

^a^The chemical formula was not disclosed

### RAS allosteric inhibitors

The interactions of KRAS-GTP or KRAS-GDP are closely linked to the activated state of KRAS and subsequently affect its signal transmissions. In general, the inactive KRAS conformation that is involved in KRAS-GDP binding is preferred.^43^ Structural biology analysis has identified two switches, switch I and switch II, on the surface of the KRAS protein that change their status based on the binding status of KRAS. The switch II region is particularly significant because of its high conformational variability, which provides an entry point for allosteric regulation. Allosteric inhibitors that interact with mutant amino acids in switch II region can induce conformational changes, resulting in a more inactive KRAS conformation. Thus, the development of allosteric inhibitors that specifically target KRAS and inhibit its abnormal function presents a promising approach to target KRAS mutants.^253^

The advantages of irreversibility provided by covalent bonding have made covalent inhibitors an effective approach to inhibiting RAS mutations. In fact, the success of RAS inhibitors provides a classic example of covalent inhibitors as well as allosteric modulators. Most of the covalent KRAS inhibitors mentioned earlier, such as the approved Sotorasib (AMG510) and Adagrasib (MRTX849), achieve inhibitory effects by stabilizing the conformation of KRAS^G12C^ mutants in an inactive state through covalent binding to residues in the allosteric site. This highlights the significance of covalent inhibitors and allosteric regulation in drug discovery, particularly in targeting KRAS-related diseases.

#### KRAS^G12C^ allosteric inhibitors

The KRAS^G12C^ mutant protein contains a mutant cysteine, Cys12, which provides a potential covalent site for inhibitors. When an inhibitor with a covalent warhead binds covalently to the mutant cys12, it induces a new allosteric pocket, S-II P, in the switch II region. The small molecule inhibitor then interacts with the corresponding amino acid, resulting in a conformational change of the KRAS protein. These effects reduce the affinity between GTP and KRAS, prevent GDP from being replaced by GTP through GEF catalysis, and ultimately lock KRAS mutants in an inactive state.^51^ Based on this action site, several small-molecule inhibitors targeting KRAS have been developed, almost all of which are covalent and were discussed in the previous chapter. These include Sotorasib (AMG-510), Adagrasib (MRTX-849), ARS-853, ARS-1620, LY-3537982, GDC-6036 (RG-6300), D-1553, ARS-3248 (JNJ-74699157), JDQ-443, and SML series compounds, among others, such as LY-3537982, ARS-853, ARS-1620, and 6H05 series compounds (Table 2).

#### KRAS^G12D^ allosteric inhibitors

KRAS^G12D^ is a more prevalent KRAS mutation type that is found in various cancers, including pancreatic cancer, colorectal cancer, and lung adenocarcinoma. As such, it is a potential target for the development of selective KRAS mutation inhibitors. However, effectively targeting other KRAS mutants presents several challenges that must be overcome. Unlike KRAS^G12C^, KRAS^G12D^ lacks an active residue near the switch II binding pocket, which prevents the protein from undergoing covalent modification. Therefore, new approaches are required to design selective inhibitors with high affinity and drug potency for KRAS^G12D^ and other non-C mutant KRAS mutations.

Mirati Therapeutics has identified and characterized a selective, non-covalent, high-affinity KRAS^G12D^ inhibitor, known as MRTX-1133 (**79**) (Table 2).^254^ The inhibitor binds to the inactive form of KRAS^G12D^ with an IC_50_ < 2 nM, demonstrating an approximately 700-fold selectivity compared to KRAS^WT^. MRTX-1133 also inhibits the binding of RAF-RAS binding domain peptides to the active form of KRAS^G12D^ with an IC_50_ of 9 nM, and induces conformational changes in switch I and switch II regions of KRAS protein.^254^ By interacting with aspartic acid Asp12 and glutamic acid Glu62 in the switch II region of KRAS protein, MRTX-1133 plays an allosteric role, resulting in conformational changes of KRAS protein and inhibition of the KRAS signaling pathway in cells and tumor environments containing KRAS^G12D^ mutations, thereby achieving an antitumor effect. In cell studies, MRTX-1133 exhibited a concentration-dependent inhibition of key KRAS pathway signaling molecules in KRAS^G12D^ mutated HPAC (pancreatic cancer) and GP2D (colorectal cancer) cell lines, including the phosphorylation of extracellular signal-regulated kinase 1/2 (pERK), the phosphorylation of S6 (pS6), the phosphorylation of 4EBP1 (p4EBP1), and the expression of dual specificity phosphatase 4 or 6 (DUSP4/6). Besides, MRTX-1133 inhibited KRAS-dependent signaling and promoted tumor regression in xenograft models.^255–258^

### SHP2 allosteric inhibitors

Src homology 2-containing protein tyrosine phosphatase 2 (SHP2) is a non-receptor protein tyrosine phosphatase (PTP) encoded by *PTPN11* gene.^259^ As protein tyrosine phosphorylation plays an essential role in multiple intracellular processes, SHP2 is involved in the regulation of multiple signaling pathways, including those involved in cancer cells, such as RAS-MAPK, PI3K-AKT and JAK-STAT pathways. Besides, SHP2 is related to some functions of PD-1/PD-L1, thus playing a role in the regulation of immune system.^260–264^ In addition, SHP2 overexpression or activation can mediate drug resistance in various cancers, including leukemia, non-small cell carcinoma, and breast cancer. Therefore, SHP2 has been considered a potential therapeutic target for cancer therapy.^265–268^

Structurally, SHP2 contains two SH2 domains (N-SH2 and C-SH2) at the N-terminal, a catalytic PTP domain, and two phosphorylable tyrosine residues (Tyr542 and Tyr580) at the C-terminal. Typically, the interaction between the N-SH2 domain and the PTP domain leads to an auto-inhibited closed conformation of the SHP2 protein.^260,269^ Upon stimulation stimulated by growth factors or cytokines, the SHP2 protein is activated, exposing the catalytic PTP domain, due to the occupation of SH2 domain thus the blocking of N-SH2-PTP interaction. As a consequence, the catalytic site of SHP2 is available to its substrates, and subsequent signal transductions could be activated. Initially, attempts to regulate SHP2 focus on identifying conventional competitive inhibitors specific to the PTP domain, also known as orthosteric inhibitors. Some designed compounds and natural products such as PHPS1, GS-493 and NSC-87877 were proposed.^270^ However, these orthosteric inhibitors are suffered from low selectivity in highly homologous proteins (SHP1, SHP2 and PTP1B) leading to toxicity and low cell permeability, thereby resulting in poor bioavailability. As a result, SHP2 has been considered an “undruggable” target protein for a long time.^271^

The concept of protein allostery has given rise to renewed hope in the development of drugs that target SHP2. Allosteric modulators work by directly stabilizing the autoinhibited conformation of SHP2, which prevents interactions between the catalytic PTP domain and SHP2 substrates. Over the past few decades, numerous SHP2 inhibitors of this type have been identified through high-throughput screening. Many of these inhibitors have progressed to clinical trials, although none have yet been approved for clinical use (Table 2).

#### SHP2 allosteric inhibitors in clinical trials

Novartis utilized a high-throughput screening method to select TNO-155 (**80**), based on the important preclinical small molecule inhibitor of SHP2, SHP099 (see “Preclinical allosteric inhibitors” for details). This process involved structural modifications and a comprehensive evaluation of inhibitory activity and physicochemical properties, including hERG inhibition, lipophilicity, and cell permeability.^272^ TNO-155 is an allosteric inhibitor of SHP2 that possesses excellent preclinical antitumor activity and pharmacokinetic data: good water solubility (0.736 mM), moderate lipophilic activity (log *P* = 1.6), high lipophilic efficiency (>6), no hERG inhibition (IC_50_ > 30 μM) and no phototoxicity.^272,273^ This compound is mainly used to treat various types of cancer, including colorectal cancer, lung cancer, NSCLC, solid tumors, tumor metastases and malignancies, etc. The phase I clinical trial of TNO-155 (NCT03114319) commenced in April 2017, and it has since undergone several clinical trials (NCT04000529, NCT04330664, NCT05541159, NCT05490030) to assess its safety, tolerability, and preliminary efficacy in combination with MRTX849, spartanizumab, or ribociclib for selected malignancies.

JAB-3068 (**81**), an allosteric inhibitor, was independently designed and developed by Jacobio. It is noteworthy that JAB-3068 has received FDA approval for clinical development after TNO-115, making it the second SHP2 inhibitor in the world to have achieved this milestone.^274^ Additionally, JAB-3068 has received orphan drug recognition from the FDA for its potential use in treating esophageal cancer. Preclinical studies have confirmed that JAB-3068 acts downstream of the PD-1 signaling pathway and can relieve immunosuppression in the tumor microenvironment. This compound is primarily used to treat advanced solid tumors, including esophageal tumors, metastatic head and neck cancer, and metastatic NSCLC, among others. The first clinical trial of JAB-3068 (NCT03518554) was initiated in April 2018 to evaluate the drug’s dose-escalation study in adult patients with advanced solid tumors. However, the current research status of this trial is unknown. Currently, clinical phase I/II trials (NCT04721223, NCT03565003) are underway to evaluate the safety, tolerability, pharmacokinetics, and antitumor activity of JAB-3068 as a single agent and in combination with JS001, an anti-PD-1 monoclonal antibody, for patients with advanced solid tumors.

RMC-4630 (**82**) is an oral allosteric inhibitor of SHP2 developed by Revolution Medicines. It selectively inhibits SHP2-dependent RAS signal mutations, including KRAS^G12C^, NF1, BRAF, and KRAS amplification. By disrupting RAS/MAPK pathway initiation, it significantly inhibits cancer growth in animal models of lung, skin, colon, and pancreatic cancers. RMC-4630 is indicated for the treatment of advanced solid tumors, colorectal cancer, metastatic NSCLC, NSCLC, and pancreatic tumors.^275^ In August 2018, clinical phase I trials of RMC-4630 were initiated, and it has now progressed to a phase II trial (NCT05054725) evaluating its combination with sotorasib in subjects with KRAS^G12C^ mutations after failure of prior standard therapy.^276^ Other clinical trials (NCT03634982, NCT03989115, NCT04916236) evaluating RMC-4630 as a single agent and in combination with other agents, such as cobimetinib, are ongoing. A phase I/II clinical trial (NCT03989115) was completed in February 2022, but the results are yet to be published.

JAB-3312 (**83**) is an allosteric inhibitor of SHP2 independently designed and developed by Jacobio. It blocks KRAS-MAPK signaling pathway, relieves the immunosuppressive microenvironment of tumors, and enhances the efficacy of existing tumor immunotherapy. Preclinical studies have demonstrated that JAB-3312 can be used to treat a variety of solid tumors, including esophageal tumors, head and neck tumors, advanced solid tumors, metastatic NSCLC, metastatic colorectal cancer, squamous cell carcinoma, and other indications.^277^ Clinical phase I trials of JAB-3312 (NCT04045496) began in August 2019. In 2020, the drug was granted orphan drug status by the FDA for the treatment of esophageal cancer. In June 2020, Abbvie and Jacobio formed a global strategic partnership to jointly develop and commercialize JAB-3068 and JAB-3312. Currently, the fastest trials involve clinical phase I/II trials (NCT05288205, NCT04720976) evaluating the safety, tolerability, pharmacokinetics, and antitumor activity of JAB-21822 in combination with JAB-3312 in patients with advanced solid tumors carrying KRAS^G12C^ mutations. Additional phase I trials (NCT04121286) are underway to evaluate the safety, tolerability, pharmacokinetics, and antitumor activity of JAB-3312 in adults with advanced solid tumors.

RLY-1971 (**84**) is an allosteric inhibitor of SHP2 developed by Relay Therapeutics, which has demonstrated significant antitumor activity in preclinical studies and may delay or overcome drug resistance. In December 2020, Relay Therapeutics entered into a collaboration with Roche to jointly develop and commercialize RLY-1971.^278^ The phase I trial (NCT04252339) of RLY-1971 was initiated in January 2020 to assess its efficacy in patients with advanced or metastatic solid tumors. The trial was completed in November 2022 and the results are currently pending.

MD Anderson Cancer Center has discovered BBP-398 (**85**) as a novel SHP2 allosteric inhibitor that specifically target SHP2 (IC_50_ = 15.7 nm) and has no inhibitory effect on SHP1. It is primarily intended for patients with advanced or metastatic KRAS^G12C^ mutated NSCLC, KRAS^G12C^ mutated solid tumors other than NSCLC, and other solid tumors with altered MAPK pathways. Navire Pharma, a subsidiary of Bridge Bio, is developing BBP-398 in collaboration with Lintobio, a Chinese company.^279^ The phase I clinical trial of BBP-398 (NCT04528836) began in July 2020. Currently, several clinical trials have reached phase I (NCT05621525, NCT05480865, NCT05375084) to evaluate BBP-398 alone or in combination with KRAS^G12C^ inhibitor (sotorasib) and programmed death receptor-1 blocking antibody (nivolumab), assessing the safety, tolerability, pharmacokinetics, and initial anticancer activity in patients with advanced solid tumors and advanced NSCLC with KRAS mutations.

ERAS-601 (**86**) is an oral selective SHP2 allosteric inhibitor developed by Erasca as a best-in-class drug. It has demonstrated antitumor activity in RAS/MAPK-driven tumor models and is selective for SHP2 with no significant inhibition against any off-target kinase or phosphatase among the 300 kinases and 12 phosphatases tested. In a series of human cancer cell line models of oncogenic alterations in the RAS/MAPK pathway, ERAS-601 showed antiproliferative activity. ERAS-601 also inhibited the growth of tumors with EGFR, KRAS, BRAF III and NF1 LOF mutations in a variety of RAS/MAPK-driven CDX and PDX models.^280^ In December 2020, ERAS-601 entered clinical phase I trials (NCT04670679) to evaluate its safety and efficacy in a dose-escalation/expansion study involving patients with advanced or metastatic solid tumors. ERAS-601 is currently in phase I/II clinical trials (NCT04959981, NCT04866134) either alone or in combination with other agents to evaluate its anticancer therapy studies targeting MAPK pathways in patients with advanced NSCLC. Additionally, the compound is being studied in combination with ERAS-007, an ERK1/2 inhibitor, for combination therapy in patients with advanced or metastatic solid tumors (HERKULES 1).

SH3809 (**87**) is a small molecule allosteric inhibitor targeting SHP2 developed by Nanjing Sanhome Pharmaceutical Co Ltd. In vitro enzyme activity inhibition assays and cell viability inhibition assays have demonstrated that SH3809 inhibits SHP-2 enzyme activity and inhibits tumor growth. Additionally, in vivo xenograft tumor animal models based on human NSCLC NCI-H358 cells and human esophageal squamous carcinoma KYSE520 cells have shown that SH3809 could provide better therapeutic options for patients with solid tumors.^281^ On 19 December 2020, SH3809 tablets received clinical trial approval from the FDA, and a phase I trial (NCT04843033) was initiated in April 2021 to determine the safety, tolerability, pharmacokinetics, and initial efficacy of SH3809 tablets in Chinese patients with advanced solid tumors.

ET-0038 (**88**) is an allosteric inhibitor of SHP2 with global intellectual property rights, developed independently by Etern Biopharma. Preclinical studies have shown that ET-0038 is highly effective in inhibiting SHP2 activity in a variety of tumor models in vitro and in vivo that carry carcinogenic mutations in the RTK/RAS pathway, resulting in significant antitumor activity. Moreover, preclinical translational medicine studies have demonstrated that ET-0038 has significant synergistic antitumor effects with various drugs targeting the RTK/RAS pathway or cell cycle regulation. It also shows favorable inhibition against osimertinib-resistant tumors caused by EGFR^C797S^ mutation or c-Met amplification.^282^ The phase I clinical trial of ET-0038 (NCT05354843) began in March 2022, and a subsequent phase I trial (NCT05525559) was conducted to investigate the efficacy of ET0038 monotherapy in the treating advanced solid tumors.

ICP-189 (**89**) is a highly selective oral SHP2 allosteric inhibitor developed by Inno Care Pharma Ltd. Preclinical studies have shown that ICP-189 is highly selective for SHP2 and has no significant inhibition against other phosphatases. In vivo efficacy studies have demonstrated significant antitumor effects of ICP-189 in various xenograft models, particularly in the treatment of advanced solid tumors.^283^ In April 2022, a phase I trial (NCT05370755) was initiated to evaluate the safety, tolerability, pharmacokinetics, and initial antitumor activity of ICP-189, both as monotherapy and in combination with anti-PD-1 monoclonal antibody, in patients with advanced solid tumors.

#### SHP2 allosteric inhibitors in preclinical research

In 2015, Novartis discovered a weak inhibitor of SHP2, SHP836 (IC_50_ = 12 μM), through high throughput screening. The compound binds to a “tunnel pocket” rather than the PTP domain, which stabilizes the inactive autoinhibitory conformation of SHP2 and prevents it from functioning as a phosphatase.^284^ Subsequently, building on SHP836 as a lead compound, Novartis researchers developed SHP099 (**90**) (IC_50_ = 0.07 μM), a compound with good selectivity and activity and good oral bioavailability.^285^ The reporting of SHP099 is a milestone for small molecule SHP2 allosteric inhibitors, which further led to the development of aforementioned SHP2 inhibitor in clinical trials, TNO-155.^286,287^ SHP099 has favorable physicochemical properties, showing selectivity against 21 phosphatases and 66 kinases. It binds simultaneously to the interfaces of the N-SH2, C-SH2 and PTP structural domains of SHP2, creating hydrogen bonding interactions with key amino acid residues (Arg111, Phe113 and Glu250) and stabilizing SHP2 in a closed, self-inhibited conformation.^288^ This mechanism of action inhibits SHP2 activity through a metastable mechanism, leading to the inhibition of RAS/ERK signaling and suppressing RTK-driven proliferation of human cancer cells in vitro.^286–288^

Revolution Medicines has been developing variant inhibitors targeting SHP2, and RMC-4550 (**91**) (IC_50_ = 0.583 nM) is a potent and selective SHP2 inhibitor that has shown efficacy against human cancer models with RAS-GTP-dependent oncogenic BRAF, NF1 deletion, or nucleotide cycle oncogenic RAS (e.g., KRAS^G12C^).^289^ RMC-4550 reduces oncogenic RAS/RAF/MEK/ERK signaling and cancer growth by disrupting SOS1-mediated RAS-GTP. Furthermore, RMC-4550 inhibits the growth of multiple subtypes of lung, melanoma, colorectal, and pancreatic cancer cells.^290,291^

PCC0208023 (**92**) is a promising SHP2 metastable inhibitor developed by Tian’s team at Yantai University, utilizing a novel azetidine-substituted aryl sequence reported by Novartis, NI-1.^287^ Using NI-1 as a template compound, PCC0208023 was designed via a scaffold hopping strategy. The study demonstrated that PCC0208023 has an IC_50_ value of 2.10 nM for the SHP2 holoenzyme and lacks activity against the SHP2 free catalytic domain. Inhibition of the RAS/MAPK signaling pathway was confirmed by the reduction of p-MEK, p-ERK, p-SHP2, and RAS-GTP levels in LS180 and HCT116 cells after PCC0208023 treatment, which also inhibited the proliferation of CRC cell lines. Furthermore, in an in vivo study, PCC0208023 demonstrated antitumor efficacy in a subcutaneous LS180 xenograft model, significantly reducing tumor weight and growth without any significant effect on mouse body weight.^292^

Sun’s team from Nanjing University and Yu’s team from Zhengzhou University developed a screening method for the enzymatic activity of SHP2-related proteins and identified TK-453 (**93**) (*K*_d_ = 150 nM) as a selective SHP2 metastable conformation inhibitor. TK-453 binds to the N-SH2, C-SH2 and PTP structural domains of SHP2 proteins at the interface to stabilize the inactive self-inhibited conformation of SHP2. Moreover, TK-453 exhibited a notable capacity to inhibit protease activities of SHP2^WT^ with an IC_50_ value of 0.023 nM, outperforming its impact on SHP2-related proteins (SHP2^E76K^, SHP2^PTP^) as well as their homologs (SHP1 and PTP1B).^293^ In an animal model of imiquimod (IMQ)-induced psoriasis, TK-453 demonstrated potential therapeutic effects by ameliorating macrophage inflammation in mice through the inhibition of the IL-23/Th17 axis. These findings suggest SHP2 as a promising therapeutic target for psoriasis.^294^

### GPCR allosteric modulators

G protein-coupled receptors (GPCRs) play essential roles in human physiology and are a major target for many pharmaceuticals on the market.^295^ While initial efforts have focused on developing orthosteric inhibitors that target active site endogenous ligands (orthosteric site), the selectivity of these inhibitors has been hindered by the high conservation of orthosteric binding sites in GPCRs.^296,297^ Therefore, allosteric modulators have opened up new possibilities for controlling GPCR activity by designing different subtype cooperativities based on distinct allosteric pockets, or by the cooperating with orthosteric modulators.^298^

Recent discoveries of allosteric sites in GPCRs, along with ligand- or structure-based allosteric drug discovery and design, have resulted in the approval of several drugs, including avacopan, cinacalcet, ticagrelor, and maraviroc, as a complement C5a receptor treatment for anti-neutrophil cytoplasmic autoantibody-associated vasculi, a CaS calcium-sensing receptor (CaSR) PAM for hyperparathyroidism and calciphylaxis, a purinergic receptor P2Y12 antagonist for the prevention of thrombosis, and a C-C motif chemokine receptor NAM for HIV infection, respectively.^299–302^ Currently, four GPCR allosteric inhibitors have been marketed, and dozens of molecules are being tested in clinical trials of phases I-III, targeting various GPCRs, along with hundreds of promising GPCR allosteric modulators in preclinical researches.

## PPI inhibition

Protein–protein interactions (PPIs) play a crucial role in numerous biological processes, including signal transduction, cell proliferation, growth, differentiation, apoptosis.^303–305^ However, abnormalities in PPIs can result in a various diseases, such as cancers, infectious diseases, and neurodegenerative diseases.^306–308^ Undruggable proteins, which possess flat active sites, often act through PPI networks, which are characterized by large, flat, and undefined interfaces between proteins. These networks have been considered undruggable biological processes for decades.^309–311^ Therefore, designing drugs for PPIs is an effective strategy to target undruggable proteins, even though it is more challenging to design modulators that target PPI interfaces compared to those that target protein-ligand interactions (found in enzymes, ion channels, or receptors).

To date, numerous modulators have been developed to target PPIs, and many of them have successfully entered the clinic.^312–316^ In terms of structure types, existing PPI inhibitors can be classified into several categories, including small molecules, antibodies, peptides or recombinant proteins, each of which has its advantages and disadvantages.^317^ Small molecules are the preferred choice of pharmaceutical chemists due to the desirable pharmacokinetic properties, such as cell membrane penetration and oral bioavailability, and their accessibility at low costs both in time and economy. However, their suitability for tight and narrow PPI interfaces and poor selectivity limits their application in targeting PPIs.^305^ In comparison, therapeutic antibodies are more suitable for the generally wide PPI interfaces with stronger targeting specificity and high efficiency, but correspondingly large molecular weight limits their application to extracellular targets. Besides, adverse reactions related to immune response caused by monoclonal antibodies cannot be ignored. Peptides or combinate proteins are designed based on the hot-spots, with retained key properties in binding to targets, which can form a stronger affinity and targeting specificity with target proteins. However, peptides and proteins have general physicochemical properties, such as instability under physiological conditions, poor oral bioavailability and solubility, which make them less optimal drugs.^19^

**Fig. 3:**
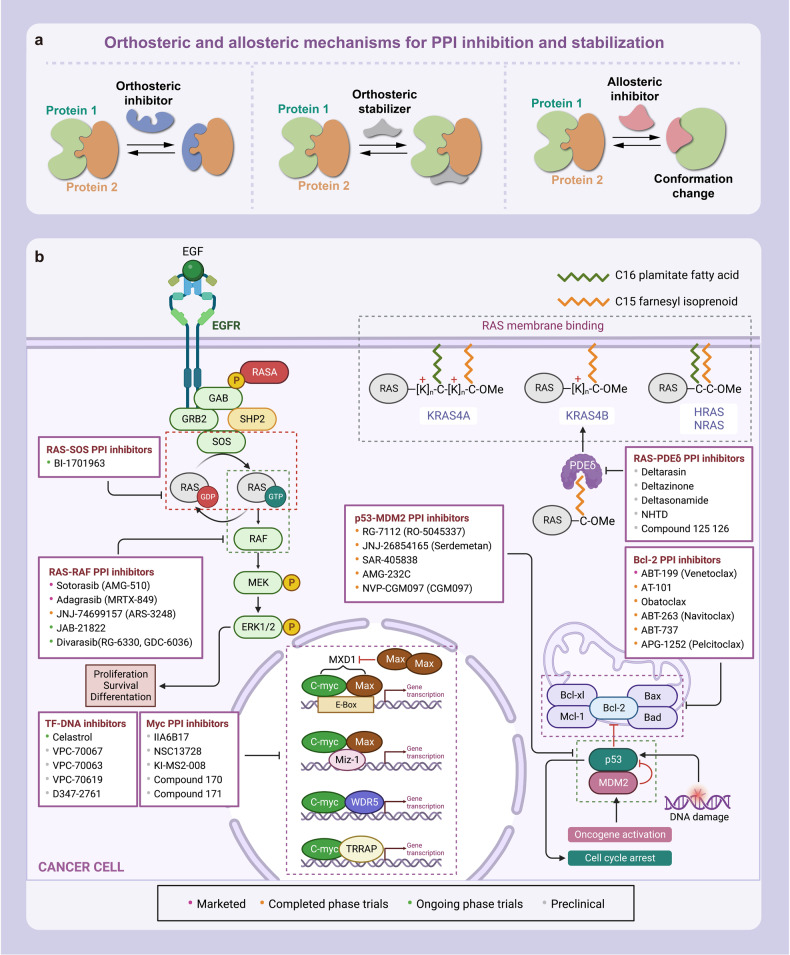
PPI inhibitors and protein–DNA interaction inhibitors targeting undruggable proteins. **a** Binding modes and mechanisms of PPI modulators: according to binding modes, PPI modulators can be categorized as orthosteric or allosteric modulators; according to mechanisms, PPI modulators can be categorized as PPI inhibitors and PPI activators. **b** Map of marketed, clinical and preclinical PPI modulators and protein-DNA interaction inhibitors in signaling pathways. PPI inhibitors targeting RAS include RAS-RAF inhibitors, RAS-SOS inhibitors and RAS-PDEδ inhibitors. Bcl-2 PPI inhibitors restore the function of pro-apoptotic proteins such as Bax and Bad through blocking their interactions with anti-apoptotic protein Bcl-2. P53-MDM2 inhibitors restore the tumor-suppressing function of p53. PPI inhibitors targeting Myc include Myc-Max inhibitors, Myc-WDR5 inhibitors and Myc-TRRAP inhibitors. Protein-DNA interaction inhibitors have been used in targeting undruggable TFs

The discovery of “hot spots” has made it possible to target PPIs effectively.^318^ Hot spot residues that form binding sites between proteins are called PPI interfaces.^319–321^ Depending on whether inhibitors bind to PPI interfaces or not, PPI modulators can be categorized as orthosteric inhibitors or allosteric modulators. Orthosteric inhibitors are designed to target PPI interfaces by identifying hot spots, while allosteric modulators induce a conformational change in the target proteins by regulating their non-interaction regions when there are no hot spots defined on PPI interfaces.^322^ Similarly, PPI modulators can be classified into non-covalent ones and covalent ones according to the binding mode. Therefore, some of cases described above in covalent inhibitors and allosteric modulators are also PPI inhibitors due to their mechanism of PPI modulation. In this section, we summarize recent advances in drugging undruggable PPI-related proteins such as RAS, Bcl-2, p53, Myc through PPI modulations (Table 3 and Fig. 3).

**Table 3:** Protein–protein/DNA modulators targeting undruggable proteins

Compound name and structure	Target	Cancer cell line (activity)	Indications	Status/clinical trial identifier	Ref.
BI-1701963 (**118**)^a^	KRAS-SOS2	–	Adenocarcinoma, colorectal cancer, NSCLC	*Completed* NCT04975256(I) *Ongoing* NCT04111458(I) *Terminated* NCT04835714(I), NCT04627142(I)	^334^
Deltarasin (**119**)	KRAS-PDE*δ*	HCT-116, Hke3, Hkh2	–	*Preclinical*	^341,342^
Deltazinone (**120**)	KRAS-PDE*δ*	HCT-116, Hke3, Hkh2	–	*Preclinical*	^343^
Deltasonamide (**121**)	KRAS-PDE*δ*	HCT-116, Hke3, Hkh2	–	*Preclinical*	^344,345^
Triazole PDE*δ* inhibitor (**122**)	KRAS-PDE*δ*	A549	–	*Preclinical*	^346^
NHTD (**123**)	KRAS-PDE*δ*	A549 (IC_50_ = 6.36 μM)	–	*Preclinical*	^347^
Deltaflexin (**124**)	KRAS-PDE*δ*	HT116 (IC_50_ = 11 μM)	–	*Preclinical*	^348^
Quinazolinone-based inhibitor (**125**)	KRAS-PDE*δ*	–	–	*Preclinical*	^353^
Quinazolinone-based inhibitor (**126**)	KRAS-PDE*δ*	Capan-1 (IC_50_ = 12.4 μM)	–	*Preclinical*	^353^
Compound (**127**)	KRAS-PDE*δ*	–	–	*Preclinical*	^354^
Compound (**128**)	KRAS-PDE*δ*	–	–	*Preclinical*	^354^
Compound (**129**)	KRAS-PDE*δ*	Capan-1 (IC_50_ = 8.8 μM)	–	*Preclinical*	^354^
Compound (**130**)	KRAS-PDE*δ*	–	–	*Preclinical*	^355^
Compound (**131**)	KRAS-PDE*δ*	Mia PaCa-2 (IC_50_ = 6.3 μM)	–	*Preclinical*	^355^
MK-1084 (**132**)^a^	KRAS^G12C^	–	NSCLC	*Ongoing* NCT05067283(I)	^681^
RMC-6236 (**133**)	RAS	–	Colorectal cancer, NSCLC, etc.	*Ongoing* NCT05379985(I)	^682^
ABT-199 (Venetoclax) (**134**)	Bcl-2/Bax	–	Acute myelogenous leukemia, chronic lymphocytic leukemia	*Marketed*	^361^
AT-101 (**135**)	Bcl-2/Bcl-xl/Mcl-1	–	B-cell lymphoma, non-Hodgkin lymphoma	*Completed* NCT00286780(II), etc. *Ongoing* NCT05603572(I/II), NCT05338931(I/II), etc. *Withdrawn* NCT00934076(I) *Terminated* NCT01003769(I/II), etc.	^683^
Obatoclax (**136**)	Bcl-2/Bcl-xl	–	Small cell lung cancer	*Completed* NCT00684918(II), NCT00359892(II), etc. *Withdrawn* NCT01238146(I), etc. *Terminated* NCT00918931(II), etc.	^684^
ABT-263 (Navitoclax) (**137**)	Bcl-2/Bcl-xl	–	Myelofibrosis	*Completed* NCT01557777(II), etc. *Ongoing* NCT04472598(III), NCT04468984(III), etc. *Withdrawn* NCT00918450(II), etc. *Terminated* NCT01828476(II), etc.	^685^
ABT-737 (**138**)	Bcl-2/Bcl-xl	–	Myelodysplastic syndrome	*Completed* NCT01440504(II)	^686^
APG-1252 (Pelcitoclax) (**139**)	Bcl-2/Bcl-xl	–	Peripheral T cell lymphoma, NSCLC, etc.	*Completed* NCT03080311(I) *Ongoing* NCT05186012(I/II), etc. *Withdrawn* NCT04354727(I/II) *Terminated* NCT03387332(I), etc.	^687^
RG-7112 (RO-5045337) (**140**)	P53-MDM2	–	Hematological neoplasm	*Completed* NCT01677780(I), NCT01164033(I), NCT01143740(I), NCT00623870(I), NCT01635296(I), NCT01605526(I), NCT00559533(I)	^369^
JNJ-26854165 (Serdemetan) (**141**)	P53-MDM2	–	Prostate tumor, NSCLC	*Completed* NCT00676910(I)	^372^
MK-8242 (SCH900242) (**142**)	P53-MDM2	–	Acute myelogenous leukemia	*Terminated* NCT01451437(I), NCT01463696(I)	^377^
SAR-405838 (**143**)	P53-MDM2	–	Neoplasm malignant, lymphoma; malignancy	*Completed* NCT01636479(I), NCT01985191(I)	^381^
AMG-232 (**144**)	P53-MDM2	–	Glioblastoma, gliosarcoma, acute myelogenous leukemia, etc.	*Completed* NCT02110355(I), etc. *Ongoing* NCT03031730(I), NCT03217266(I) *Suspended* NCT04190550(I), etc.	^384^
NVP-CGM097 (**145**)	P53-MDM2	–	Solid tumor	*Completed* NCT01760525(I)	^388^
DS-3032 (Mliademetan) (**146**)	P53-MDM2	–	Liposarcoma	*Completed* NCT03671564(I), etc. *Ongoing* NCT05012397(II), NCT04979442(III) *Terminated* NCT03552029(I), etc.	^391^
NVP-HDM201 (**147**)	P53-MDM2	–	Myelofibrosis, acute myelogenous leukemia	*Completed* NCT02890069(I), etc. *Ongoing* NCT05180695(I/II), NCT04097821(I/II); NCT04116541(II), etc. *Withdrawn* NCT03760445(I/II) *Terminated* NCT04496999(I), etc.	^394^
ALRN-6924 (**148**)^a^	P53-MDM2	–	Peripheral T cell lymphoma, bone marrow depression	*Completed* NCT02264613(I/II), etc. *Ongoing* NCT05622058(I), etc. *Terminated* NCT04022876(I)	^395^
RG-7388 (Idasanutlin) (**149**)	P53-MDM2	–	Multiple myeloma, glioblastoma, colorectal carcinoma, acute myelogenous leukemia, etc.	*Completed* NCT02407080(I), etc. *Ongoing* NCT02633059(I/II), NCT04029688(I/II) *Terminated* NCT02545283(III), etc.	^399^
APG-115 (Alrizomadlin) (**150**)	P53-MDM2	–	Chronic myelogenous leukemia, myelodysplastic syndromes, stage IV melanoma	*Completed* NCT02935907(I) *Ongoing* NCT04785196(I/II); NCT04496349(II); NCT04358393(I/II), etc.	^403^
BI-907828 (**151**)^a^	P53-MDM2	–	Biliary tumor	*Ongoing* NCT05218499(II/III), etc.	^407^
ASTX-295 (**152**)	Myc-Max	–	Advanced solid tumor	*Ongoing* NCT03975387(I), ASTX295-01 (I/II)	^409^
OMO-103 (**153**)	Myc-Max	–	Advanced solid tumor	*Ongoing* NCT04808362(I/II)	^421^
IIA6B17 (**154**)	Myc-Max	NGP19, NB11, HCT-116-29	–	*Preclinical*	^688^
IIA4B20 (**155**)	Myc-Max	–	–	*Preclinical*	^689^
IIA3B16 (**156**)	Myc-Max	–	–	*Preclinical*	^689^
IA5B12 (**157**)	Myc-Max	–	–	*Preclinical*	^689^
10058-F4 (**158**)	Myc-Max	HL-60 (IC_50_ = 41.1 μM)	–	*Preclinical*	^472^
Compound **159**	Myc-Max	HL-60 (IC_50_ = 4.6 μM)	–	*Preclinical*	^690^
KJ-Pyr-9 (**160**)	Myc-Max	HL-60 (IC_50_ = 23 μM)	–	*Preclinical*	^691^
10074-G5 (**161**)	Myc-Max	HL-60 (IC_50_ = 22.5 μM)	–	*Preclinical*	^472^
JY-3-094 (**162**)	Myc-Max	HL-60	–	*Preclinical*	^692^
3jc48-3 (**163**)	Myc-Max	HL-60 (IC_50_ = 23 μM)	–	*Preclinical*	^692^
sAJM589 (**164**)	Myc-Max	P493-6 (IC_50_ = 1.9 μM)	–	*Preclinical*	^693^
MYCMI-6 (**165**)	Myc-Max	IMR-32, Kelly, SK-N-DZ, SK-N-F1, SK-N-AS and SK-N-RA	–	*Preclinical*	^694^
MYCi975 (**166**)	Myc-Max	P493-6 (IC_50_ = 3.7 μM)	–	*Preclinical*	^695^
NSC13728 (**167**)	Myc-Max	MCF7-35IM	–	*Preclinical*	^423^
KI-MS2-001 (**168**)	Myc-Max	P493-6 (IC_50_ = 1.98 μM)	–	*Preclinical*	^422^
KI-MS2-008 (**169**)	Myc-Max	P493-6 (IC_50_ = 1.28 μM)	–	*Preclinical*	^422^
Macdonald’s research Compound (**170**)	Myc-Max	HEK293T (IC_50_ = 29 μM)	–	*Preclinical*	^428^
Macdonald’s research Compound (**171**)	Myc-Max	HEK293T (IC_50_ = 100 μM)	–	*Preclinical*	^429^
Curcumin (**172**)	Myc-Max	–	–	*Preclinical*	^431^
Super-TDU (**173**)^a^	YAP-TEAD	MGC-803, BGC-823, HGC27	–	*Preclinical*	^437^
Verteporfin (**174**)	YAP-TEAD	–	Choroidal neovascularization, wet age related macular degeneration	*Preclinical*	^440^
MGH-CP1 (**175**)	YAP-TEAD	HEK293		*Preclinical*	^438^
ICG-001 (**176**)	β-catenin-TCF	MCF7, SW480	–	*Preclinical*	^448^
NLs-Stax-h (**177**)	β-catenin-TCF	SW-480, DLD-1	–	*Preclinical*	^449^
iCRT3 (**178**)	β-catenin-TCF	–	–	*Preclinical*	^450^
iCRT5 (**179**)	β-catenin-TCF	–	–	*Preclinical*	^450^
iCRT14 (**180**)	β-catenin-TCF	HCT116	–	*Preclinical*	^450^
Henryin (**181**)	β-catenin-TCF	–	–	*Preclinical*	^451^
PKF115-584 (**182**)^a^	β-catenin-TCF	HEK293T	–	*Preclinical*	^452^
CGP049090 (**183**)	β-catenin-TCF	Human GBM cell lines, T98G, U87, MCF-7	–	*Preclinical*	^452^
GDC-0152 (**184**)	XIAP-caspase-9	–	Solid tumors	*Terminated* NCT00977067(I)	^459^
GDC-0917 (CUDC-427) (**185**)	XIAP-caspase-9	–	Lymphoma	*Completed* NCT01908413(I) *Terminated* NCT01226277(I)	^460^
LCL-161 (**186**)	XIAP-caspase-9	–	Myelofibrosis, breast tumor, ovary tumor, multiple myeloma	*Completed* NCT01617668(II), etc. *Terminated* NCT02649673(I)	^461^
AT-406 (Xevinapant, Debio1143) (**187**)	XIAP-caspase-9	–	Metastatic head and neck cancer, squamous cell carcinoma	*Completed* NCT01078649(I) *Terminated* NCT01265199(I)	^462^
Birinapant (TL32711) (**188**)	XIAP-caspase-9	–	Squamous cell carcinoma	*Completed* NCT01940172(I), etc. *Ongoing* NCT03803774(I) *Withdrawn* NCT01766622(II), NCT02756130(I/II) *Terminated* NCT01681368(II), NCT02587962(I/II), etc.	^463^
ASTX-660 (**189**)	XIAP-caspase-9	–	Lymphoma, cutaneous T cell lymphoma, peripheral T cell lymphoma, adult T cell lymphoma	*Completed* NCT04411030(I), etc. *Ongoing* NCT05082259(I), NCT04362007(I/II), NCT02503423(I/II), *Terminated* NCT04155580(I)	^464^
Celastrol (**190**)	Myc-Max-DNA	–	Cancer, cardiac reperfusion injury	*Ongoing* NCT05413226(NA), NCT05494112(NA)	^471^
VPC-70067 (**191**)	Myc-Max-DNA	–	–	*Preclinical*	^472^
VPC-70063 (**192**)	Myc-Max-DNA	LNCaP (IC_50_ = 2.5 μM)	–	*Preclinical*	^472^
VPC-70619 (**193**)	Myc-Max-DNA	NCI-H660 (IC_50_ = 7 μM)	–	*Preclinical*	^473^
D347-2761 (**194**)	Myc-Max-DNA	Human myeloma cells, RPMI-8226, NCI-H929	–	*Preclinical*	^474^

Data collected from https://clinicaltrials.gov [last accessed March 2023]

^a^The chemical formula was not disclosed

### RAS PPI inhibitors

RAS acts as a molecular switch, transmitting signals of cell growth and differentiation by binding to GTP or GDP and switching between active and inactive states.^261^ When activated, RAS can activate multiple downstream signaling pathways. RAF-MEK-ERK is the most typical RAS effect pathway. Activated RAS proteins can interact with many downstream effector proteins, such as RAF kinase, son of sevenless (SOS), phosphatidylinositol 3-kinase (PI3K) and Ral guanine nucleotide dissociation stimulating factors (RalGDS), which regulate cell events such as cell proliferation and apoptosis.^323–325^

Designing PPI inhibitors to block the interaction of RAS and RAS effect factors represents a promising paradigm for modulating RAS function and provides new impetus for the discovery of related drugs.^86,326–329^

#### RAS-RAF inhibitors

Monomeric proteins, based on the synthetic protein (FN3) skeleton, are similar in function to antibodies and can be used in combination with the corresponding target. These proteins have a smaller molecular weight and clearer structure compared to traditional antibodies. Moreover, they are more stable in oxidation/reduction conditions, making them a new type of ideal inhibitor.^330,331^

Spencer-Smith et al. recently reported a novel allosteric inhibitor that targets protein interactions between RAS and RAF.^329^ The researchers used monomeric protein libraries to find a monomeric protein, NS-1, that specifically binds to HRAS but not NRAS. The binding sites of NS-1 and HRAS are located in the α4, α5, and β6 regions of the RAS protein opposite the RAS/RAF interface. The binding of NS-1 can allosterically induces structural changes in the SW-I and SW-II domains, thus disrupting the interaction between RAS and effector proteins, including RAF. Electron microscopy, BRET assay, and immunoprecipitation assay all confirmed that the interaction between RAS and RAF was destroyed by NS-1. Further cell experiments demonstrated that NS-1 could effectively inhibit the activation of MAPK signaling pathway downstream of the RAS protein in cells. Experiments in bladder cancer cells showed that NS-1 can inhibit endogenous mutated RAS signal and corresponding cell proliferation. These results suggest that disrupting the protein interaction between RAS and RAF through allosteric effects can inhibit RAS protein-mediated carcinogenesis, providing a new idea for the development of anti-cancer therapies targeting RAS proteins.

#### RAS-SOS inhibitors

SOS (son of sevenless) is a guanine nucleotide exchange factor (GEF) that is essential for the activation of KRAS proteins in cells and closely related to the expression of KRAS and its downstream signaling pathways. Therefore, developing KRAS inhibitors targeting SOS is considered a viable alternative strategy for countering KRAS mutations.^332^ The RAS-SOS complex alters the conformation of the RAS protein, blocking the hydrophilic interaction of the magnesium cofactor with the GDP’s phosphate group, thus reducing the affinity of the RAS for GDP and facilitating its release. GTP’s guanine riboside and ribose fractions then begin to bind to RAS, followed by the interaction of the γ-phosphate group of GTP with the magnesium cofactor of RAS, resulting in another conformational change of RAS and the detachment of SOS from the complex.^333^ Therefore, developing inhibitors targeting the RAS-SOS PPI is a promising strategy.

Boehringer Ingelheim (BI) developed BI-1701963 (**118**), the first pan-KRAS inhibitor, which shows broad activity against KRAS alleles (G12D, G12V, G12C and G13D) while preserving the KRAS- SOS2 interaction. BI-1701963 has potential applications in the treatment of various diseases, including adenocarcinoma, advanced solid tumors, biliary tract cancer, colorectal cancer, lung tumors, metastatic colorectal cancer, and metastatic pancreatic cancer.^334^ It has entered phase I clinical trials (NCT04111458) in October 2019 to explore different doses of BI-1701963 alone and in combination with trametinib, a MEK inhibitor, in patients with different types of advanced cancer (KRAS mutant solid tumor). Meanwhile, a phase I clinical trial (NCT04975256) was completed in November 2022, but the results are pending publication. In addition, after analyzing safety, efficacy, and PK data, BI terminated the phase I trial of BI-1701963 alone or in combination with the MEK inhibitor BI-3011441 (NCT04835714) and the phase I trial of BI-1701963 in combination with irinotecan in patients with advanced colorectal cancer with KRAS mutation (NCT04627142).

#### RAS-PDEδ inhibitors

Targeting proteases that regulate RAS binding to cell membranes is a promising development strategy for indirect targeting of KRAS, such as inhibiting the activity of farnesyltransferase and PDE*δ*. The KRAS protein is located on the inner side of the cell membrane and is attached to the cell membrane by a modification group of farnesyl. Farnesyl groups are added to KRAS proteins by post-translational protein modification under the action of farnesyltransferase. KRAS can only be fully activated by upstream signaling proteins when it is located on the cell membrane.^335^ Therefore, inhibiting the activity of farnesyltransferase can prevent the farnesylation of KRAS and reduce its localization to the cell membrane, ultimately inhibiting the activity of KRAS.^336^

PDE*δ* (phosphodiesterase 6 delta) is a protein that binds to KRAS protein and regulates its orientation to membrane compartments.^337,338^ PDE*δ* has a large hydrophobic pocket that can bind farnesyl proteins, especially the lipid portion of KRAS protein, thereby preventing the farnesyl modification of KRAS protein from binding to cell membrane and inhibiting tumor cell proliferation and growth.^339,340^

Through fragment-based molecular docking and high-throughput virtual screening, various KRAS-PDE*δ* inhibitors have been developed subsequently, including the triazole inhibitor, compound (**122**), tetrahydrodibenzofuran inhibitor, NHTD (**123**), and coumarin inhibitor, deltaflexin (**124**). Of these, the triazole PDE*δ* inhibitor effectively blocked the interaction between PDE*δ* and RAS protein, exhibiting over 2000-fold greater binding activity to PDE*δ* than deltarasin in the control group.^346^ NHTD, on the other hand, redistributes the localization of KRAS to the endometrium through PDE*δ*-targeting isoprene binding pockets, and studies in vitro and in vivo have shown that NHTD can induce apoptosis and inhibit carcinogenic KRAS signaling by disrupting KRAS-PDE*δ* interactions (IC_50_ = 40 μM) in NSCLC, effectively preventing tumor growth in xenografted and KRAS-mutated mouse models.^347^ Deltaflexin similarly disrupts the KRAS–PDE*δ* interaction, selectively destroying KRAS while preserving the membrane structure of HRAS. Moreover, deltaflexin selectively inhibits oncogenic KRAS-driven cell proliferation and tumor formation.^348^

Since 2017, Sheng’s research group has dedicated their efforts to developing small molecule inhibitors that target KRAS-PDE*δ* interactions. In 2017, they developed a novel quinazolinone-based small molecule inhibitor of KRAS-PDE*δ* by analyzing “hot spots” and conducting virtual screenings. By employing structure-based virtual screening, they identified a quinazolinone fragment, hit 4 (*K*_d_ = 467 ± 65 nM), with two optical isomers that acted on different “hot spots” of PDEδ.^349–352^ They then used fragment-based drug design to obtain four isomers, of which compound (**125**) with R and R configuration was the most active (*K*_d_ = 2.3 nM). Building on this, they optimized the structure to yield compound (**126**), a highly active small molecule KRAS-PDE*δ* inhibitor (*K*_d_ = 0.6 nM). Ultimately, both compound (125) and compound (126) effectively inhibited KRAS-PDE*δ* PPI, disrupted the correct localization of KRAS on the cell membrane, down-regulated the phosphorylation of key proteins Erk and Akt in the downstream pathway of KRAS, and induced apoptosis in KRAS-mutated pancreatic cancer cell lines.^353^

Later, after conducting molecular docking studies, they identified two fragments, quinazolinone fragment hit 4 and benzimidazole fragment 5, which showed the ability to inhibit the interaction between PDE*δ* and KRAS in a specific manner. Building upon these findings, the group developed a series of PDE*δ* inhibitors, including compound (**127**) (*K*_d_ = 9 ± 2.3 nM) and compound (**128**) (*K*_d_ = 168 ± 25 nM). Through structural optimization, they were able to synthesize compound 8, which contains a cyclopropyl group that forms hydrophobic interactions with the amino residues Ile129, Val145, and Leu147 in PDE*δ*. Compound (**129**) exhibited superior affinity for PDE*δ* (*K*_d_ = 38 ± 17 nM) and was found to be a more potent inhibitor of Capan-1 cells (IC_50_ = 8.8 ± 2.4 μM) than compound (**127**) and compound (**128**).^354^

As efficacy of known KRAS-PDE*δ* inhibitors is often weak in vivo, the Sheng research team then screened the library built by their own group to identify novel KRAS-PDE*δ* inhibitors with enhanced antitumor efficacy. A novel spirocline KRAS-PDE*δ* seedling compound (**130**) (*K*_d_ = 1471 ± 246 nM) was screened out and subjected to further structure optimization, leading to the discovery of compound 36I (*K*_d_ = 127 ± 16 nM). Compound (**131**) is a highly active helicoid KRAS-PDE*δ* inhibitor that down-regulates the phosphorylation of key proteins Erk and Akt in the downstream pathway of KRAS, and induces the apoptosis of KRAS-mutated pancreatic cancer cell lines. Compound (**131**) has demonstrated potent anti-pancreatic cancer activity both in vivo and in vitro. It is the first KRAS-PDE*δ* inhibitor to show in vivo efficacy in the PDX model, making it a promising candidate for further study. Additionally, it is the most effective small molecule inhibitor of KRAS-PDE*δ* reported to date, suggesting its potential as a therapeutic agent for the treatment of pancreatic cancer.^355^

### Bcl-2 PPI inhibitors

Bcl-2 family plays a crucial role in the endogenous apoptosis signal transduction pathway by regulating the mitochondrial/cytochrome C-mediated apoptosis process.^356,357^ This family consists of anti-apoptotic and pro-apoptotic proteins that act synergistically as apoptotic switches, ultimately determining the cell’s fate.^358–360^ Among them, Bcl-2 is an anti-apoptotic protein that is highly expressed in various hematological malignancies, including chronic lymphocytic leukemia (CLL) and mantle cell lymphoma (MCL), making it an attractive drug target. Pro-apoptotic proteins such as Bax and Bad are essential for the cell apoptosis process, but their function is inhibited when they bind to anti-apoptotic proteins like Bcl-2. Consequently, blocking the interaction between pro-apoptotic and anti-apoptotic proteins can prevent tumor cells from escaping apoptosis. However, the approximate area of the PPI interface of the Bcl-2 and Bak/Bax is 750-1500 Å2, which is flat and lacks grooves. the lack of suitable molecules to serve as a starting point for further development has long rendered Bcl-2 undruggable. This changed with the advent of “alanine scanning” technology, which identified hot spots and paved the way for further drug development of Bcl-2 PPI inhibitors.

Numerous Bcl-Bax inhibitors, including ABT-199 (venetoclax), ABT-737, ABT-263 (navitoclax), etc., have been developed. Among them, ABT-199 (134) is the first and currently the only approved small-molecule Bcl-2 inhibitor.^361^ Abbvie researchers introduced indolyl and azoindole groups into the skeleton structure of ABT-263 to create ABT-199 and studied its structure–activity relationship. The results revealed that ABT-199 exhibits potent activity against Bcl-2-dependent hematologic carcinoma and has a significant inhibitory effect on ALL cells with high Bcl-2 expression. ABT-199 is also effective in treating chronic lymphocytic leukemia by inducing cell apoptosis, leading to the elimination of tumor cells from the bloodstream. Notably, ABT-199 caused less damage to platelets than the second-generation drug ABT-263 in both in vitro and in vivo studies.^362–364^ Venetoclax, the generic name for ABT-199, has been approved by the FDA for treating adults with CLL or small lymphoblastic lymphoma (SLL). Clinical trials have demonstrated that the combination of venetoclax with rituximab, an anti-CD20 monoclonal antibody, is more effective than venetoclax monotherapy. Bcl-2 PPI inhibitors currently in clinical use and undergoing clinical trials are listed in (Table 3).

### P53-MDM2 PPI inhibitors

The p53 protein, a crucial tumor suppressor, plays a significant role in preventing cancer initiation and progression.^206–208^ MDM2, the most important negative regulator of p53, directly binds to p53 and forms complexes with it, regulating the stability and activity of p53 protein.^215^ MDM2 binds to the N-terminus of the transcriptional activation domain of p53, inhibiting the trans-activation of p53 and cell growth, regulating cell cycle and inducing cell apoptosis.^365–368^ Therefore, disrupting the MDM2-p53 interaction, presents a potential approach for treating cancer by restoring the impaired function of p53.^216,217^ Currently, a range of MDM2-p53 inhibitors have entered clinical trials, but some compounds have been discontinued for various reasons.

RG-7112 (**140**), developed by Roche, is a small molecule MDM2 inhibitor designed to occupy the p53 binding site of MDM2, clinically proven with an IC_50_ value of 18 nM and *K*_d_ value of 11 nM binding MDM2. It can be taken orally and can cross the blood-brain barrier. By binding to MDM2, RG-7112 inhibits the interaction between p53 and MDM2, preventing the degradation of the p53 proteasome, which leads to stabilization and increased levels of the tumor suppressor protein p53 in the cell.^369^ In vitro experiments showed that RG-7112 can inhibit and kill SJSA-1 osteosarcoma cells with high expression of MDM2 protein, inducing dose-dependent cell cycle arrest in G1 and G2/M phases in HCT116 and SJSA1 cells. In vivo studies show that a single oral administration of RG-7112 can activate p53 pathway, induce tumor cell apoptosis, reduce tumor growth and improve survival in GBM models.^370,371^ The earliest phase I clinical trial of RG-7112 (NCT00559533) began in November 2017. Currently, RG-7112 has completed several phase I clinical trials for the treatment of diseases including chronic myeloid leukemia, acute myeloid leukemia, solid tumors, hematoma, although no results have been posted.

JNJ-26854165 (**141**), also known as serdemetan, is an orally bioavailable compound that blocks the binding of the MDM2-p53 complex to the proteasome and inhibits the degradation of p53, thereby restoring p53-mediated apoptosis of tumor cells.^372^ Preclinical studies have shown that JNJ-26854165 exhibited strong activity against multiple myeloma (MM) cells both in vivo and in vitro. However, it was p53 independent in p53 mutants and nutlin-3a resistant cases. Meanwhile, its activation of E2F1 further confirmed the independent character of p53. The IC_50_ value of JNJ-26854165 against p53 mutant non-Hodgkin’s lymphoma cell lines was higher than that of wild-type p53 mutant non-Hodgkin’s lymphoma cell lines, and induced type I and type II programmed death of wild-type and mutant p53 cells, respectively. More preclinical studies reported that JNJ-26854165 increased p53 levels in U87 glioblastoma grafts, accompanied by apoptotic stimulation.^373–376^ In addition, JNJ-26854165 induces antitumor effects in breast, lung, colon, and prostate tumor models with mutant p53. JNJ-26854165 showed a series-specific antiproliferative effect and a higher anticancer effect when used in combination with other antitumor agents, such as rapamycin, doxorubicin, Bcl-2 inhibitors. These important preclinical studies have advanced the drug to clinical trials. A phase I clinical trial of JNJ-26854165 (NCT00676910) began in May 2008 and was completed in March 2010, with no further progress reported.

MK-8242 (SCH900242) (**142**), developed by Merck, is a potent inhibitor of MDM2 that prevents the interaction between HDM2 and p53, thereby rescuing p53 function. It exhibited a strong inhibitory effect on wild-type PPTP cells with an IC_50_ value of 0.07 μM, but had weak inhibitory effects on TP53-mutant PPTP cells with an IC_50_ value greater than10 μM), indicating a selectivity of more than 100 times. In vivo studies demonstrated that MK-8242 delayed tumor growth by a factor of two or more in 10 of 17 solid tumor xenografts containing wild-type TP53, but had no effect on osteosarcoma xenografts with low TP53 expression.^377–380^ MK-8242 is primarily used for the treatment of acute myeloid leukemia and advanced solid tumors. Clinical trials for MK-8242 were initiated in October 2011, evaluating its efficacy in the treatment of acute myeloid leukemia (P07649) (NCT01451437) in monotherapy or in combination with cytarabine, a DNA-directed DNA polymerase inhibitor. Another clinical trial (NCT01463696) is to evaluate its safety and pharmacokinetics in patients with advanced solid tumors (P07650). However, these trials were terminated in 2018 with no results reported.

SAR-405838 (**143**) is a spiroxindole-based MDM2 inhibitor that demonstrates strong binding affinity to MDM2 and effective growth inhibition activity. It has shown high selectivity for cancer cell lines with mutations or deletions of p53. In the SJSA-1 model, SAR-405838 effectively inhibited the growth of tumor cell lines and induced apoptosis in ABTR1 and ABTR2 sublines in a dose-dependent manner. In mouse xenograft models of SJSA1, RS411, LNCaP, and HCT-116, SAR-405838 achieved lasting tumor regression or complete inhibition of tumor growth under a well-tolerated dose regimen.^381,382^ Phase I clinical trials for SAR-405838 were initiated in July 2012 for related malignancies through NCT01985191 and NCT01636479. These trials were completed or terminated in 2016 and 2018, respectively. However, no results have been published as of yet.

AMG-232 (**144**) is a potent and highly selective oral piperidone inhibitor of MDM2-p53 interaction, with an IC_50_ of 0.6 nM and MDM2-binding *K*_d_ of 0.045 nM.^383,384^ Studies have shown that AMG-232 is more selective than RG-7112 for p53 wild cells. In vitro experiments demonstrated that AMG-232 induced p53 signal transduction and inhibited tumor cell proliferation in three p53 wild tumor cell lines (SJSA-1, HCT116, and ACHN). In vivo experiments showed that AMG-232 (10, 25, 75 mg kg^−1^, orally, once a day) activated the p53 pathway, effectively inhibited the growth of xenogeneic tumors in mice, blocked viral DNA synthesis, induced apoptosis, and caused dose-dependent tumor growth inhibition (ED_50_ = 16 mg kg^−1^).^385–387^ The phase I clinical trial of AMG-232 (NCT01723020) began in November 2012. Phase I clinical trials (NCT01723020, NCT02016729, NCT02110355) evaluating its efficacy in patients with advanced solid tumors or MM and metastatic melanoma have been completed, with results pending publication. Currently, phase I clinical trials (NCT03031730, NCT03217266) are ongoing for plasma cell tumor, acute myeloblastic leukemia, and glioblastoma.

NVP-CGM097 (**145**) is a highly effective and selective MDM2 inhibitor developed by Novartis. It binds to the p53 binding site of MDM2 protein and disrupts the interaction between proteins, leading to the activation of the p53 pathway. In wild-type p53 cells, NVP-CGM097 significantly induced the redistribution of p53 into the nucleus (IC_50_ = 0.224 μM).^388^ In a mouse model of human cancer, NVP-CGM097 exhibits favorable pharmacokinetic characteristics and impacted pharmacodynamic markers in human tumors, leading to tumor regression at well-tolerated dose levels. In human p53 wild-type tumor cells, NVP-CGM097 binds to human MDM2 protein with a Ki value of 1.3 nM, activating p53 in human cells and inducing p53-dependent cell cycle arrest and apoptosis.^389,390^ In January 2013, NVP-CGM097 was enrolled in a phase I clinical trial (NCT01760525) to evaluate the dose-escalation study of NVP-CGM097 in adults with advanced solid tumors. The trial was completed in 2021, but the results are currently pending publication.

DS-3032 (**146**), also known as mliademetan, is an innovative, specific, oral small molecule inhibitor of MDM2 that disrupts the MDM2-p53 interaction in tumor cells. It c has shown great potential in the study of acute myeloid leukemia and is currently being developed as a cancer therapy based on the p53 reactivation mechanism.^391^ In preclinical studies, DS-3032 has been found to selectively and dose-dependently induce apoptosis in neuroblastoma cells with wild-type TP53, while having no effect on TP53-mutated Kelly cells. Furthermore, in vivo pharmacodynamic experiments of DS-3032 on SH-SY5Y xenograft nude mice have demonstrated that DS-3032 activated the TP53 signaling pathway in transplanted tumor tissue, inhibited tumor growth, and prolonged the survival of mice.^392^ In addition, DS-3032 treatment has been found to increase apoptotic cells and decrease proliferative cells. DS-3032 has undergone clinical phase I trial (NCT01877382) since June 2013, and a number of clinical trials have been completed (NCT03671564, NCT03647202, NCT03634228, NCT03614455, NCT01877382), but the results have yet to be published. Unfortunately, the phase I trials of DS-3032 (NCT03552029, NCT02319369) were terminated due to a business decision by the developer. Currently, DS-3032 is being tested in a phase II clinical trial in advanced/metastatic solid tumors (NCT05012397) and a phase III clinical trial in combination with trabectedin for dedifferentiated liposarcoma (NCT04979442).

NVP-HDM201 (**147**), also known as siremadlin, is an imidazolpyrrolidone analog developed by Novartis that has demonstrated highly favorable in vivo properties as an orally effective and selective MDM2-p53 inhibitor and recently entered phase I clinical trials in cancer patients. It prevents p53 degradation by interfering with the interaction between human and mouse MDM2-p53, and has good pharmacokinetic and pharmacodynamic characteristics in animals, as well as good oral bioavailability. In preclinical studies, both once daily and once every three weeks dosing regimens showed comparable long-term efficacy.^393,394^ Phase I trials of NVP-HDM201 (NCT02143635) began in May 2014 and was completed in 2020, with results pending. However, two phase I trials of NVP-HDM201 (NCT04496999 and NCT02601378) have been discontinued due to lack of recruitment and business consolidation. Nevertheless, a number of clinical trials (NCT05180695, NCT05599932, NCT03940352, NCT03714958, NCT04097821, NCT04116541, NCT05447663, NCT05155709) are still being conducted for the treatment of myelofibrosis, acute myeloid leukemia, colorectal cancer, advanced cancer and more.

ALRN-6924 (**148**) is a staple peptide drug developed by Aileron to target a variety of cancers by reactivating p53-mediated tumor inhibition. ALRN-6924 is a staple peptide drug that combines the α-helical conformation of peptides to overcome the stability and membrane permeability issues that commonly affect traditional peptide drugs.^395^ Specifically, it targets two major p53 suppressor proteins, MDMX and MDM2, to induce cell cycle arrest and raise p21, a known inhibitor of the cell replication cycle. In preclinical mouse models, ALRN-6924 has shown activity as a radio-protectant that also prevents chemotherapy-induced toxicity.^217,395,396^ It has also been shown to promote the recruitment of tumor-infiltrating immune cells, especially CD8+ killer T cells, and increase tumor-suppressing M1 macrophages. The drug has exhibited strong targeting activity in vitro ACUTE MYELOGENOUS LEUKEMIA cell lines and primary cells, induced apoptotic cell death in TCL cell lines, and significantly reduced tumor burden.^397,398^ It is mainly used for acute leukemia, ACUTE MYELOGENOUS LEUKEMIA, myelosuppression, brain tumor, and began its first clinical trials in October 2014. A safety study of ALRN-6924 in patients with acute myeloid leukemia or advanced myelodysplastic syndrome (NCT02909972) and a phase I/II trial in patients with advanced solid tumors or lymphoma (NCT02264613) have been completed, but results are pending. A clinical trial (NCT04022876) evaluating its preventive effect on side effects of chemotherapy has been terminated due to a lack of statistically significant results. Nevertheless, several phase I clinical trials (NCT05622058, NCT03725436, NCT03654716) of ALRN-6924 in patients with TP53 breast cancer and in combination with paclitaxel, a naturally occurring antineoplastic agent, to treat whom in advanced, metastatic, or unresectable solid tumors are still ongoing.

RG-7388 (**149**), also known as idasanutlin, is the first potent oral MDM2 protein inhibitor developed by Roche.^399^ With its exceptional activity and selectivity, RG-7388 can effectively activate the p53 pathway, induce wild type p53 expression, trigger cell cycle block or apoptosis, and inhibit tumor proliferation in neuroblastoma transplant tumor. Notably, RG-7388’s inhibitory activity (IC_50_ = 6 nM) is three times that of RG-7112 (IC_50_ = 18 nM).^399,400^ In a mouse SJSA osteosarcoma xenograft model, oral administration of RG-7388 (25 mg kg^−1^) induced tumor growth inhibition and regression, as well as apoptosis and anti-proliferation induction.^401,402^ Clinical trials of RG-7388 (NCT02545283) began in September 2015 and have progressed to the phase III clinical stage, focusing on treating relapsed or refractory acute myeloid leukemia (ACUTE MYELOGENOUS LEUKEMIA) in combination with cytarabine. However, this study has been discontinued due to interim analysis efficacy results. Multiple other clinical trials of RG-7388 (NCT03850535, NCT02624986, NCT03135262, NCT03566485, NCT02545283, NCT03287245) were also terminated based on poor interim analysis data. While the phase I clinical trials (NCT02407080, NCT02828930, NCT03362723, and NCT02670044) have been completed, results are pending. Currently, only phase I/II trials of RG-7388 monotherapy in relapsed MM and RG-7388 in combination with chemotherapy or venetoclax in relapsed/refractory acute leukemia or solid tumors are ongoing.

APG-115 (**150**), also referred to as alrizomadlin, is a potent antitumor drug that was independently designed and developed by Yashang Pharmaceutical. The drug has been granted global intellectual property rights and acts on the novel target MDM2-p53. APG-115 is the first MDM2-p53 inhibitor to undergo clinical trials in China, thus filling a crucial gap in the field of drug development for this target in the country.^403^ APG-115 has significant activity, which is more than 10 times that of SAR-405838, and can overcome the chronic isomerization of SAR-405838.^404,405^ In vitro studies have shown that APG-115 has an impact on disease progression by inducing AGS and MKN45 cells with wild-type p53 to be blocked in the G0/G1 phase. It has also been found to activate p53, enhance the radiosensitivity of AGS and MKN45 cells, and induce concentration-dependent G2/M phase arrest and S phase reduction in wild-type p53 cell lines (TPC-1, KTC-1). In vivo experiments have demonstrated that when combined with radiotherapy, APG-115 can enhance the in vivo radioantitumor effect of gastric adenocarcinoma.^406^ APG-115 received FDA approval to enter clinical trials in June 2016 and is currently in clinical I b/II trials (NCT03611868) in combination with pembrolizumab, a PD-1 inhibitor, in solid tumors in the United States. In July 2017, it was approved by CFDA to carry out clinical trials and became the first new drug MDM2-p53 inhibitor to undergo clinical trials in China. A phase I trial of APG-115 in patients with advanced solid tumors or lymphoma has been completed, but results are still pending. Seven clinical trials are currently underway for the treatment of T-lymphoblastic leukemia, liposarcoma, advanced solid tumors, lymphoma, acute myeloid leukemia, metastatic melanoma, malignant salivary adenocarcinoma, and myelodysplastic syndrome.

BI-907828 (**151**) is an orally effective MDM2 inhibitor developed by BI with potential antitumor activity. When taken orally, it binds to the MDM2 protein, preventing the binding of the p53 transcription-activated region, thus restoring p53 transcriptional activity which mediates tumor cell apoptosis. Compared to other MDM2 inhibitors, its pharmacokinetic properties allow for dose optimization, potentially reducing myelosuppressive side effects.^407,408^ A phase I clinical trial (NCT03449381) has been conducted since February 2018 to assess the optimal dose of BI-907828 for patients with different types of advanced cancer (solid tumors). Additionally, ongoing phase I trials are being conducted in patients with biliary, pancreatic, lung or bladder cancers.

ASTX-295 (**152**) is an MDM2 inhibitor developed by Astex that blocks MDM2-p53 interactions. In a panel of p53 wild-type ACUTE MYELOGENOUS LEUKEMIA cell lines, ASTX-295 showed a high anti-proliferative effect, with GI_50_ < 30 nM in 9 out of 11 cell lines. Treatment with ASTX-295 alone induced apoptosis in both ACUTE MYELOGENOUS LEUKEMIA cell lines and primary ACUTE MYELOGENOUS LEUKEMIA with combination of ASTX-295 and decitabine increased the growth inhibitory effect compared to single agent treatments. Additionally, target engagement by ASTX-295 and decitabine was confirmed by upregulation of p53 transcriptional targets and decreased DNMT-1 expression.^409^ A phase I clinical trial of ASTX-295 began in June 2019 (NCT03975387). It is now in its phase II clinical stage and is the first phase I/II human trial in the United States (ASTX295-01). This trial will evaluate the safety, pharmacokinetics, and primary activity of ASTX-295 in 135 participants with wild-type TP53 advanced solid tumors.

### Myc PPI inhibitors

The Myc oncogene, including its three subtypes C-Myc, N-Myc, and L-Myc, is believed to act as a transcriptional “amplifier” that can trigger a variety of oncogenic transcriptional programs in different types of cancer.^410,411^ Overexpression or hyperactivation of Myc is one of the most common occurrences in cancer, making it a key factor in oncogenesis. Myc proteins, synthesized after transcription and translation of Myc gene, plays a critical role in pathways related to tumor development, including proliferation, apoptosis, differentiation, metabolism, and adaptive cancer resistance.^412–414^ Therefore, it is a highly validated anti-cancer target. However, it remains untouchable as it lacks hydrophobic pockets or grooves suitable for binding small molecule compounds.

Myc’s large and flat interaction surface forms protein complexes, making it a difficult target for drug development. As a result, Myc has been considered an undruggable target. Until now, no drugs targeting Myc have been approved for market. However, recent years have seen an increase in information about the structure of relevant proteins and the development of new computational tools, leading to the creation of strategies for targeting Myc inhibition. Significant progress has been made in the development of inhibitors that act on Myc-PPI, including three major types: Myc-Max PPI inhibitors, Myc-WDR5 PPI inhibitors, and Myc-TRRAP PPI inhibitors.

#### Myc-Max PPI inhibitors

Max is a constitutive expression protein with ubiquitous presence that plays a central role in controlling the Max-Max dimerin 1 (MXD1) axis. C-Myc forms a complex with Max through the bHLH-ZIP domain to activate gene transcription.^415,416^ The Myc-Max complex has two regulatory mechanisms: (1) Myc-Max binds to specific recognition sites (E-Box elements) in the promoter region to activate gene transcription; (2) Myc-Max heterodimer is indirectly recruited to DNA by zinc finger protein Miz-1, thus inhibiting Myc regulatory genes.^417^ Given the dependence on Myc-Max interaction for both regulatory mechanisms, regulating this interaction is an effective method to inhibit Myc function.

Omomyc is one of the most studied c-Myc/Max inhibitors, derived from the bHLH structural domain of Myc. It competes with c-Myc for binding to Max, forming Omomyc/Max complexes that prevent the transcriptional activation of Myc target genes involved in proliferation and metabolism.^418–420^ Peptomyc’s novel c-Myc inhibitor, OMO-103 (**153**),^421^ based on the Omomyc membrane penetrating peptide, has been approved by Spanish Pharmacovigilance Agency AEMPS to enter phase I/II clinical trials (NCT04808362) to assess its safety and efficacy. Furthermore, small molecules have been screened as promising Myc-Max PPI inhibitors (Table 3).

On the other hand, formation of Max-Max homodimer provides an alternative mechanism for blocking Myc/Max interactions and preventing Myc/Max heterodimers from binding to DNA, resulting in reduced transcriptional activity. Thus, stabilizing the Max-Max homodimer can prevent Myc-Max transactivation of target genes.^422^ To this end, several active small molecules have been identified as promising Max-Max homodimer stabilizers, including NSC13728 (**167**) and KI-MS2-008 (**169**). In 2009, Jiang et al. reported the development of NSC13728, a symmetrical small molecule that stably inhibits c-Myc-regulated oncogenic transformation, cell growth, and target gene transcription through the stabilization of Max-Max homodimers.^423^ Analytical ultracentrifuge experiments have demonstrated that NSC13728 shifts the monomer/dimer equilibrium of Max towards the dimer and reduces the dissociation constant of Max-Max homodimers by 1000-fold. NSC13728 significantly inhibited the oncogenic transformation of Myc-dependent chick embryo fibroblasts and exhibited good selectivity for Src, Jun, and P3K induced transformation. Despite these promising results, the strategy of indirectly regulating Myc-driven transcription through Max/Max homodimer stabilization has not been widely explored.^424^ In 2019, Struntz et al. utilized SMM (small molecule microarray) screening to identify KI-MS2-001 (**168**) (IC_50_ = 1.98 μM), a molecule with a unique asymmetric polycyclic lactam compound.^422^ Further structural optimization led to the development of KI-MS2-008 (IC_50_ = 1.28 μM), which exhibits significantly increased binding affinity for Max-Max homodimers, reduces Myc protein levels, perturbs Myc-driven transcriptional programs, induces Myc-dependent cellular differentiation, and affects viable cell growth of both engineered and non-engineered cell lines. Experimental data indicate that KI-MS2-008 is effective in reducing tumor size at a low dose (0.06 mg·kg^−1^, iv) in mouse T-cell acute lymphoblastic leukemia (T-ALL) transplant tumor models.

#### Myc-WDR5 PPI inhibitors

WDR5 is a highly conserved WD-40 repeat protein that plays a critical role in hematopoietic processes, regulating H3K4 methylation and HOX gene expression.^425,426^ Myc-WDR5 interaction is crucial for the control of cell proliferation, differentiation, and apoptosis through the regulation of gene expression. Myc generally binds to the WBM site of WDR5, which has a distinct structural feature conserved in Myc proteins. Hence, it is well-suited for developing small molecule inhibitors of Myc-WDR5 PPI.^427^ In 2019, Macdonald et al. discovered a class of Myc-WDR5 inhibitors with a sulfonamide structure via high-throughput screening (HTS).^428^ Although compound (**170**) was the most active (IC_50_ = 29 nM), it was unsuitable for in vivo studies. To address this, Chacon Simon et al. identified several other compound fragments via NMR fragment screening, resulting in compound (**171**) (IC_50_ = 100 nM).^429^ This compound is now being used to study the biological effects of the Myc-WDR5 complex.

#### Myc-TRRAP PPI inhibitors

Transformation/transcription domain associated protein (TRRAP) is a highly conservative 434 kDa protein that belongs to the phosphatidylinositol 3-kinase related kinase (PI3K) family.^430^ It is a key cofactor of Myc and a member of the histone acetylation (HAT) complex that assists TFs such as Myc in controlling gene expression. The interaction between Myc and TRRAP takes place in a precise region of the Myc protein called MB-II, which is also at the core of the Myc transactivation domain (TAD).^431^ Despite the disordered nature of the Myc TAD, evidence suggests that MB-II becomes a well-defined structure when it interacts with TRRAP, providing an opportunity to develop inhibitors that can block Myc-TRRAP interactions and specifically target Myc-driven cancers.^432–434^

Curcumin (**172**), the primary compound in the natural product turmeric, exhibits tautomeric properties in the form of both aldehydes and ketones, with high chemical reactivity towards a range of biological targets. Its anticancer properties are attributed to its ability to prevent the formation, spread, and metastasis of tumor cells. In 2021, Alexander et al. discovered curcumin interferes with the fundamental function of Myc and induces cross-linking between this oncogenic TF and its co-activator, TRRAP.^431^ Covalent cross-linking of TRRAP with Myc can permanently isolate TRRAP, resulting in interference in the binding balance between TRRAP and its tumor suppressor partners. The c-Myc gene has been originally identified as transduced viral allele termed v-Myc in the transforming avian acute leukemia virus MC29. To test for oncogenic Myc activity, Alexander et al. consequently used an original avian cell system and the retroviral RCAS expression vector. Experiments showed that in the presence of curcumin, endogenous Myc protein levels were significantly reduced, while constitutive expression of ectopic v-Myc was almost unaffected. Consequently, the endogenous Myc levels were drastically decreased, and cell proliferation was inhibited.

### Other PPI inhibitors targeting undruggable proteins

There are several other undruggable proteins can be regulated by PPI modulation, such as YAP/TAZ, β-catenin, XIAP, etc.^19^ Among them, YAP/TAZ and β-catenin belong to TFs have traditionally been considered “undruggable” targets. YAP/TAZ primarily pairs with the TEAD family of TFs to induce gene expression signatures that play an important role in cancer development, progression, and metastasis.^435,436^ Therefore, inhibition of YAP/TAZ-TEAD is an attractive and viable new cancer treatment option. Over the years, a number of inhibitors targeting YAP/TAZ-TEAD have been reported, including peptides such as super-TDU (**173**),^437^ and small molecules such as verteporfin (**174**) and MGH-CP1 (**175**).^438–440^

β-catenin, a critical mediator of the WNT signaling pathway, plays an important role in tumorigenesis.^441–444^ When WNT ligands are present, β-catenin degradation is impeded, causing its nuclear translocation and subsequent binding to T-cell factor (TCF), which results in the transcriptional activation of their target genes.^443,445,446^ Moreover, β-catenin has been shown to stimulate c-Myc expression by activating the c-Myc promoter, which comprises multiple T cell factor-4 (TCF-4) binding sites.^447^ In addition, several compounds have been selected as inhibitors targeting β-cateninde PPI, including small molecules such as ICG-001 (**176**),^448^ NLs-StAx-h (**177**)^449^ and CRT inhibitors (iCRT3 (**178**), iCRT5 (**179**), iCRT14 (**180**)),^450^ Henryin (181),^451^ PKF115-584 (**182**) and CGP049090 (**183**).^452^

Apoptosis protein inhibitors (IAPs), such as XIAP, C-IAP1, C-IAP2, are a crucial class of endogenous anti-apoptosis proteins.^453,454^ IAPs bind to caspase or other pro-apoptotic proteins, hinder their functions, promote their degradation, and thus regulate apoptosis.^455,456^ Caspase, a major actor of apoptosis, is an aspartic proteolytic enzyme containing cysteine. Endogenous protein inhibitors of the XIAP-caspase-9 interaction are present in the form of Smac (second mitochondrial-derived caspase activator).^457^ Upon release from mitochondria, the N-terminal amino acid alanine-valine-proline-isoeuceucine (AVPI) of Smac binds to the domain of XIAP, depriving XIAP of its ability to bind to caspase, thereby promoting apoptosis.^458^ Mimics of Smac proteins that exhibit a similar affinity to XIAP can inhibit the interaction between XIAP and caspase-9. Researchers have developed related drugs, including GDC-0152 (**184**),^459^ GDC-0917 (CUDC-427) (**185**),^460^ LCL-161 (**186**),^461^ AT-406 (Xevinapant, Debio1143) (**187**),^462^ Birinapant (TL32711) (**188**),^463^ ASTX-660 (**189**).^464^

### TF–DNA interaction inhibitors

In addition to PPI inhibitors, blocking the interaction of TFs with DNA is another option, as some of these factors bind directly to DNA.^465–467^ Protein–DNA interactions are important for the function of TFs and other DNA-binding proteins, including histones, DNA methyltransferases, polymerases, and topoisomerases. Unlike the ligand binding pocket that binds to the active site of the enzyme or receptor, the protein-DNA interface of action is directly exposed to the solvent. Hence, amino acid residues with a high positive charge, such as lysine and arginine, can be directly matched to the DNA skeleton.

DNA alkylation drugs, as the first class of drugs targeting DNA, have been clinically used for nearly 70 years to treat cancers.^468^ Over the past two decades, significant advancements have been made in the development of small molecules that can specifically bind to DNA to regulate TFs activity.^469^ For example, Hiroshi Sugiyama’s group has recently developed a new class of artificial TF mimics chemicals based on pyrrole-imidazole polyamides that can target specific DNA sequences and regulate gene expression.^470^ However, no DNA-binding small molecule has been approved as a regulator of TF due to its poor specificity.

Edward et al. reported that celastrol (**190**) (Table 3), a naturally occurring triterpenoid compound, is an inhibitor of the c-Myc oncoprotein that is overexpressed in many human cancers. They demonstrated that celastrol binds to and alters the quaternary structure of the preformed dimer, thereby eliminating its DNA binding ability.^471^ Subsequently, Carabet et al. used the Site Finder module of molecular operating environment (MOE) to identify potential binding sites at the Myc-Max-DNA interface based on the crystal structure of the c-Myc-Max heterodimer bound to the DNA sequence. Two compounds, VPC-70067 (**191**) and VPC-70063 (**192**) (Table 3), were ultimately obtained.^472^

In 2022, Artem Cherkasov’s research group discovered pockets of the Myc bHLHZ domain in a previous study, leading to the discovery of the compound VPC-70063 as a c-Myc inhibitor. Through a series of molecular docking virtual screening and optimization, they subsequently obtained compound VPC-70619 (**193**) (Table 3), a potent oral active n-Myc inhibitor that binds to the DNA binding region and prevents n-Myc/Max from binding to DNA.^473^ This compound demonstrated strong inhibitory activity against n-Myc dependent cell lines and high bioavailability for oral and intraperitoneal administration. At 5 μM, it achieved nearly 100% transcriptional inhibition, and its proliferation inhibition and cell selectivity in n-Myc high expression cell lines were characterized.

In 2022, Xu Kelin’s research group employed molecular docking and screening in a compound library based on the crystal structure of the c-Myc/Max complex, and conducted experimental screening of the resulting compounds, leading to the identification of the most active compound D347-2761 (**194**) (best activity at 10 μM) (Table 3).^474^ In activity characterization experiment, D347-2761 bound to the DNA-binding region of Myc protein and demonstrated proliferation inhibitory activity on myeloma cells at 5–10 μM, with no inhibitory effect on normal cells. The compound induced cell death by promoting apoptosis. In a mouse model of osteosarcoma, D347-2761 inhibited tumor growth and reduced the expression level of Myc. It also influences the heterodimerization of c-Myc and Max, thereby affecting the stability of c-Myc and regulating downstream targeted genes.

## Targeted proteins’ regulation

**Fig. 4:**
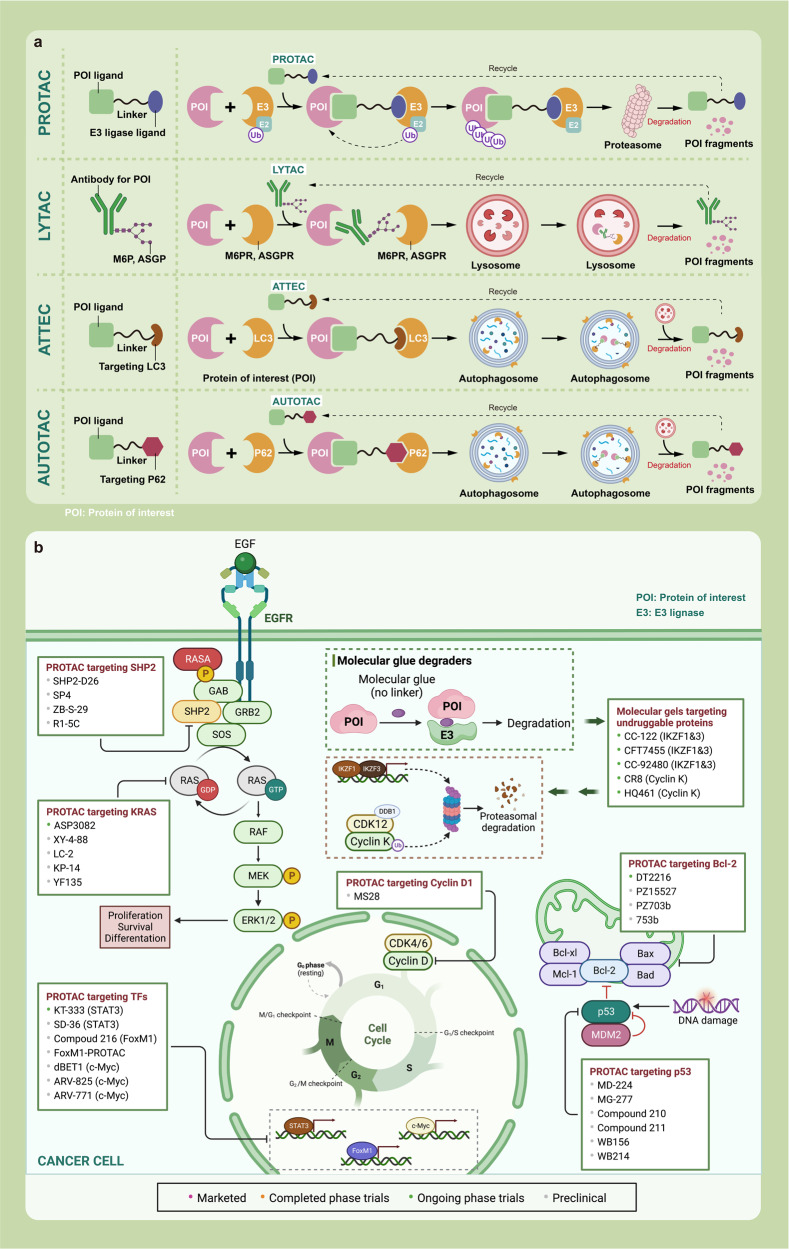
Targeted proteins regulation strategies targeting undruggable proteins. **a** Mechanisms of selected targeted proteins regulation strategies: PROTAC, LYTAC, ATTEC, and AUTOTAC utilize an “event-driven” degradation process instead of an “occupancy-driven” binding mechanism, allowing them to target and degrade undruggable proteins of interest (POIs) through proteasomal or lysosomal degradation pathways. **b** Map of marketed, clinical and preclinical targeted proteins regulation molecules in signaling pathways. PROTAC, in particular, has demonstrated the ability to degrade previously deemed undruggable POIs such as SHP2, KRAS, Bcl-2, and p53, as well as regulate TFs including STAT3, FoxM1, and c-Myc. Additionally, molecular glues have been utilized for the degradation of undruggable targets, such as IKZF and Cyclin K

Considering the inherent challenges in designing drugs for previously deemed undruggable proteins, due to the presence of flat surfaces and the lack of active sites, coupled with the direct correlation between protein levels and disease, direct regulation of disease-related proteins has emerged as an exceptionally promising therapeutic strategy.^475^ Protein regulation can be categorized into two primary classes based on the disease mechanism: protein degradation and protein stabilization.^476^ In recent years, remarkable progress in disease treatment through direct protein degradation by proteolysis-targeting chimeras (PROTAC) has underscored the importance of protein regulation drugs. Although some have entered clinical trials with a primary focus on PROTAC, the diverse range of protein regulation methods has undoubtedly laid a robust foundation for developing drugs targeting previously undruggable proteins.^477–479^ This section will offer an overview of current protein regulation technologies and their applications in addressing undruggable proteins (Fig. 4).

### Proteasome-based targeted protein degradation (TPD)

Fueled by the challenges posed by traditional small-molecule inhibitors, PROTAC emerged as a groundbreaking approach for protein regulation. At the core of PROTAC’s mechanism of action lies the degradation of target proteins via the ubiquitin-proteasome system (UPS). As a heterobifunctional molecule, PROTAC simultaneously targets the protein of interest and the E3 ubiquitin ligase, forming a ternary complex. Within this complex, the target protein is tagged with ubiquitin, facilitating its recognition and subsequent degradation by the proteasome.^480,481^ In contrast to conventional small-molecule inhibitors, PROTAC employs an “event-driven” degradation process rather than an “occupancy-driven” binding mechanism, rendering PROTAC highly efficient and capable of rapidly degrading intracellular target proteins, including drug-resistant mutants.^482^ With the potential to degrade nearly 80% of human intracellular proteins, PROTAC technology has broad applications across various fields, most notably in cancer therapy.^483^

In the following sub-sections, we will focus on the advancements made in PROTAC research and state-of-the-art TPD technologies, particularly emphasizing their application to “undruggable” targets.

#### PROTAC targeting KRAS

The KRAS gene, one of the most frequently mutated oncogenes in cancer, has long evaded drug targeting efforts due to the absence of effective druggable pockets on its corresponding proteins.^69^ In 2013, a breakthrough by a group led by Kevan Shokat reported molecules capable of covalently and selectively binding to KRAS^G12C^ mutant cysteines, reigniting hope for KRAS drug discovery. Subsequently, several oral KRAS^G12C^ inhibitors were optimized and introduced.^86^ The approval of Lumakras, an Amgen-developed drug, in May 2021 for treating NSCLC patients with KRAS^G12C^ mutations marks a significant milestone in KRAS-targeting drug discovery.^53^ However, the rapid emergence of adaptive resistance and reactivation of MAPK signaling following treatment necessitates the development of KRAS-targeted drugs utilizing TPD technologies, such as PROTACs.^484^

**Fig. 5:**
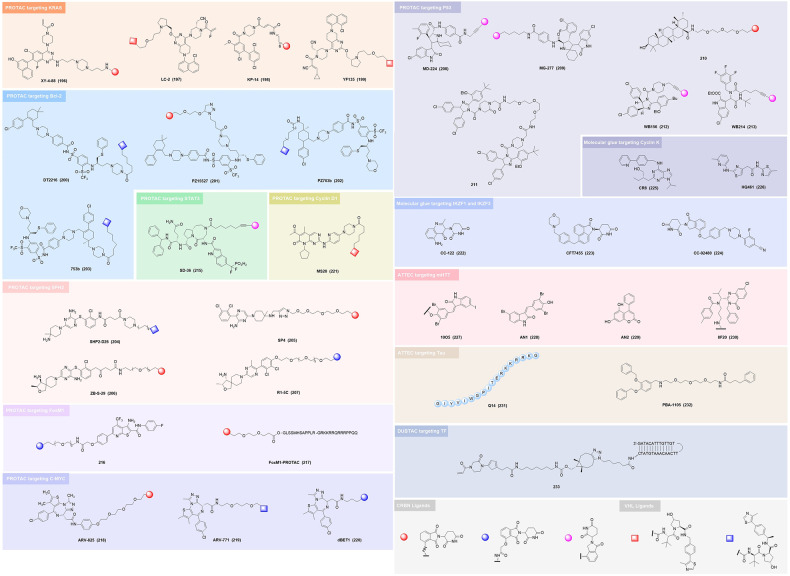
Selected chemical structures of targeted protein regulator targeting undruggable proteins. PROTAC technology targets the degradation of undruggable targets, such as KRAS, Bcl-2, Cyclin D1, STAT3, SPH2, FoxM1, C-MYC, and p53, which share two common E3 ubiquitin ligase ligands. Other innovative technologies, such as molecular glues for targeted degradation of IkZF, ATTEC for targeting mHTTh or Tau, and DUBTAC for targeting TF, have also shown promise in targeting undruggable targets

In 2019, Arvinas first patented a PROTAC-based method to degrade KRAS^G12C^, constructing various degraders using ARS-1620 derivatives and E3 ligase ligands. Their experiments showed effective KRAS^G12C^ degradation in NCI-H2030 cells, with higher drug concentrations increasing degradation rates. Although these PROTAC molecules require optimization, they demonstrate the potential for targeting KRAS^G12C^. Later, Astellas introduced its first-ever KRAS degrader, ASP3082 (**195**), to clinical trials (Table 4). Targeting KRAS^G12D^ specifically, this PROTAC is being tested in Phase I trials for patients with KRAS^G12D^ mutations in advanced or metastatic solid tumors. Astellas is also investigating the use of ASP3082 in combination with the EGFR-blocking antibody cetuximab in Phase I trials, with encouraging results indicating the potential of combining EGFR blockers with KRAS^G12C^ inhibitors to control KRAS signaling (Fig. 5).^485^

**Table 4:** Degradation of undruggable proteins by the proteasome or lysosome systems

Compound name	Target	Cancer cell line (activity)	Indications	Status/clinical trial identifier	Ref.
ASP3082 (**195**)	KRAS^G12D^	–	Solid tumor	*Ongoing* NCT05382559(I)	^485^
XY-4-88 (**196**)	KRAS^G12C^	Cereblon cellular (IC_50_ = 79 nM)	–	–	^51^
LC-2 (**197**)	KRAS^G12C^	NCI-H23 (DC_50_ = 0.25 μM)	–	–	^486^
KP-14 (**198**)	KRAS^G12C^	NCI-H358 (DC_50_ = 1.25 µM)	–	*–*	^487^
YF135 (**199**)	KRAS^G12C^	H358 (DC_50_ = 3.61 µM)	–	–	^488^
DT2216 (**200**)	Bcl-2		Solid tumor hematologic malignancy	*Ongoing* NCT04886622(I)	^492^
PZ15527 (**201**)	Bcl-2	Non-senescent (DC_50_ = 46 nM)	–	–	^493^
PZ703b (**202**)	Bcl-2	MOLT-4 (DC_50_ = 14.3 nM)	–	*Preclinical*	^494^
753b (**203**)	Bcl-2	293 T (DC_50_ = 6 nM)	–	*Preclinical*	^495^
SHP2-D26 (**204**)	SHP2	MV4; 11 (DC_50_ = 2.6 nM)	–	–	^497^
SP4 (**205**)	SHP2	Hela (IC_50_ = 4.3 nM)	–	–	^498^
ZB-S-29 (**206**)	SHP2	MV4;11 (IC_50_ = 0.207 μM)	–	–	^499^
R1-5C (**207**)	SHP2	–	–	–	^500^
MD-224 (**208**)	P53	RS4;11 (IC_50_ = 1.5 nM)	–	–	^54^
MG-277 (**209**)	P53	RS4;11 (DC_50_ = 1.3 nM)	–		^503^
Compound **210**	P53	A549 (IC_50_ = 0.23-0.39 μM)	–	–	^504^
Compound **211**	P53	A549 (IC_50_ = 1.4 μM)	–	–	^505^
WB156 (**212**)	P53	RS4; 11 (IC_50_ = 7.2 nM)			^508^
WB214 (**213**)	P53	RS4; 11 (IC_50_ = 1.2 nM)	–	–	^508^
KT-333 (**214**)	STAT3		NHL; PTCL; CTCL; LGL-L	*Ongoing* NCT05225584(I)	^511^
SD-36 (**215**)	STAT3	MOLM-16 (IC_50_ = 10 nM)	–	*Preclinical*	^512^
Compound **216**	FoxM1	TNBC MDA-MB-231 (DC_50_ = 1.96 μM)	–	–	^514^
FoxM1-PROTAC (**217**)	FoxM1		–	–	^513^
ARV-825 (**218**)	c-MYC	Molt4 (DC_50_ = 4.75 nM)	–	*Preclinical*	^518^
ARV-771 (**219**)	c-MYC	22Rv1 (IC_50_ < 1 nM)	–	*Preclinical*	^519^
dBET1 (**220**)	c-MYC		–	–	^520^
MS28 (**221**)	Cyclin D1	Calu-1 (DC_50_ = 950 nM)	–	–	^522^
CC-122 (**222**)	IKZF1 and IKZF3		Non-Hodgkin lymphoma; melanoma	*Ongoing* NCT01421524(I) NCT02031419(I) NCT02417285(I) NCT02509039(I) NCT05688475(I) *Completed* NCT02049528(I) NCT02234999(I) NCT02323906(I) NCT02406742(I/II) NCT02859324(I/II) NCT03097016(I) NCT03283202(I) NCT03340662(I) NCT03834623(II)	^526^
CFT7455 (**223**)	IKZF1 and IKZF3	–	Multiple myeloma; lymphoma; non-Hodgkin’s	*Ongoing* NCT04756726(I/II)	^527^
CC-92480 (**224**)	IKZF1 and IKZF3	–	Relapsed or refractory multiple myeloma; healthy volunteers; multiple myeloma	*Ongoing* NCT03374085(I/II) NCT03989414(I/II) NCT05372354(I/II) NCT05389722(I) NCT05519085(III) NCT05552976(III) *Completed* NCT03803644(I) NCT04211545(I) NCT04560738(I) NCT04839809(I)	^528^
CR8 (**225**)	Cyclin K	–	*Mycobacterium ulcerans* infection	*Ongoing* NCT01659437(II/III)	^530^
HQ461 (**226**)	Cyclin K	A549 (DC_50_ = 0.132 μM)	–		^531^
10O5 (**227**)	mHTT	Primary cortical neurons (the effective dose = 6.0 nM)	–	–	^539^
AN1 (**228**)	mHTT	Primary cortical neurons (the effective dose = 6.0 nM)	–	–	^539^
AN2 (**229**)	mHTT	Primary cortical neurons (the effective dose = 75 nM)	–	–	^539^
8F20 (**230**)	mHTT	Primary cortical neurons (the effective dose = 75 nM)	–	–	^539^
Q14 (**231**)	USP30	A172 (IC_50_ = 57.2 nM)	–	–	^540^
PBA-1105 (**232**)	Tau	SHSY5Y (DC_50_= ~1–10 nM)	–	–	^541^
Compound **233**	FOXO3A				^549^

Data collected from https://clinicaltrials.gov [last accessed March 2023]

Moreover, Gray’s team designed and synthesized covalent degrader molecules (PROTACs) targeting cysteine 12 (C12) in KRAS^G12C^. One compound, named XY-4-88 (**196**), displayed the ability to induce dimerization of CRBN-KRAS^G12C^ (Table 4). However, despite its covalent binding to KRAS^G12C^, XY-4-88 failed to degrade endogenous KRAS^G12C^ and did not demonstrate a degradation-dependent phenotype.^51^

Crews’s group reported the development of LC-2 (**197**) in 2020, the first PROTAC molecule capable of degrading endogenous KRAS^G12C^ (Table 4). LC-2 binds covalently to KRAS^G12C^ through the MRTX849 warhead and recruits the E3 ligase VHL. Experiments showed that LC-2 induced rapid and sustained degradation of KRAS^G12C^, inhibiting MAPK signaling in various cancer cell lines with both homozygous and heterozygous KRAS^G12C^. Notably, LC-2’s ability to overcome increased KRAS^G12C^ expression suggests that KRAS^G12C^ degraders may be more effective in maintaining suppression of downstream signaling than inhibitors alone.^486^

A year later, Chen’s team successfully designed and synthesized a series of PROTACs targeting KRAS^G12C^, employing the KRAS G12C-IN-3 inhibitor and pomalidomide as degradation agents. Among the series, the degradant KP-14 (**198**) exhibited exceptional KRAS^G12C^ degradation activity in NCI-H358 cells, with a DC_50_ of 1.25 µM (Table 4). Mechanistic experiments revealed that KP-14 selectively degraded KRAS^G12C^, but not other KRAS mutants such as G13D, through the protein-ubiquitin system. Furthermore, KP-14 displayed potent antiproliferative activity and inhibited tumor colony formation in NCI-H358 cells.^487^

One drawback of PROTACs is their irreversible binding mode, potentially impacting substoichiometric activity and reducing effectiveness. Recently, the Lu group made a breakthrough by creating the first reversible-covalent PROTAC, YF135 (**199**), which is based on cyanacrylamide and linked to a VHL ligand scaffold using MRTX849 (Table 4). YF135 demonstrated optimal degradation activity in both H358 and H23 cells, inducing KRAS^G12C^ protein degradation with DC_50_ values of 3.61 µM and 4.53 µM, respectively. The antiproliferative activity of YF135 was also observed to be favorable in both tumor cell lines, with IC_50_ values of 153.9 nM and 243.9 nM, respectively. Although YF135’s degradation activity was not as robust as LC-2’s, this study still offers a promising new direction for the development of reversible-covalent degraders.^488^

#### PROTAC targeting Bcl-2

The Bcl-2 family of proteins is integral to tumor growth and metastasis, encompassing three subgroups based on structure and function. The inhibition or degradation of apoptosis-inhibiting Bcl-2 proteins, such as Bcl-2, Mcl-1, and Bcl-xL, offers new therapeutic avenues. Nevertheless, these targets involve PPIs, and their large binding interfaces present challenges in designing small molecules to inhibit these interactions.^489^ Additionally, the extensive Bcl-2 binding pocket, containing approximately 13-15 amino acids, complicates the creation of small molecules capable of blocking the entire pocket. Consequently, Bcl-2 proteins are considered a particularly challenging class of undruggable targets.^490,491^

In 2019, Zhou’s team identified DT2216 (**200**), the first selective Bcl-xL degrader based on ABT263 (navitoclax), by linking ABT263 to VHL ligands (Table 4). DT2216 exhibited high potency against various Bcl-xL-dependent leukemia and cancer cells while demonstrating lower platelet toxicity than ABT263. In vivo, DT2216 effectively inhibited the growth of multiple xenograft tumors without causing significant thrombocytopenia.^492^ Zhou’s team also designed PZ15527 (**201**), which efficiently degraded Bcl-xL protein in non-senescent cells, reduced platelet toxicity, and caused minimal thrombocytopenia (Table 4).^493^ Furthermore, PZ703b (**202**) showed enhanced degradation activity, reduced platelet toxicity, and improved efficacy in cancer cell lines compared to ABT263 and DT2216 (Table 4).^494^ Finally, 753b (**203**) displayed high selectivity for cancer cells over normal platelets and a lower toxicity profile, making it a promising candidate for future cancer therapy (Table 4).^495^ These findings highlight the potential of TPD technology in developing safer and more effective therapeutic degrader for cancer therapy.

#### PROTAC targeting SHP2

SHP2, a non-receptor protein tyrosine phosphatase encoded by the PTPN11 gene, is widely expressed in adult tissues and associated with numerous cancers. Efficient and selective inhibition of SHP2’s catalytic activity center has proven challenging due to the positively charged and highly conserved PTP structural domains present in the PTP family.^496^ As a result, SHP2 has been deemed an “undruggable” target. However, recent efforts have focused on direct SHP2 degradation as a promising approach for inhibiting tumor cell growth and reducing drug resistance in cancer therapy.

In this context, Wang’s group recently reported on the first PROTAC molecule (SHP2-D26) (**204**) targeting SHP2 (Table 4). This degrader employs a VHL ligand to recruit an E3 ubiquitin ligase, facilitating SHP2 protein degradation. Researchers tested SHP2-D26’s efficacy in KYSE520 and MV4;11 cell lines, observing dose-dependent degradation with DC_50_ values of 6.0 nM and 2.6 nM, respectively. At a concentration of 30 nM, the degradation rate exceeded 95% in both cell lines. SHP2-D26’s degradation kinetics were investigated in KYSE520 and MV4;11 cells, where it effectively reduced SHP2 protein within 4 h and nearly completely degraded it at 8 h. Notably, SHP2-D26 was more effective than control SHP2 inhibitors in suppressing ERK phosphorylation and cancer cell growth.^497^

In addition to SHP2-D26, two other SHP2 degraders were designed using thalidomide as the ligand, linked to the SHP2 inhibitors SHP099 or TNO155 through varying lengths of linkers. These compounds, SP4 (**205**) (SHP2WT IC_50_ = 4.3 nM in Hela cells)^498^ and ZB-S-29 (**206**)^499^ (SHP2WT IC_50_ = 0.207 μM in MV4;11 cells), effectively degraded SHP2 in KYSE520 cells (Table 4). Another degrader, R1-5C (**207**), employed pomalidomide as the ligand and connected it to RMC-4550 via a PEG linker, showcasing superior SHP2 degradation (Table 4). By inducing SHP2 degradation and inhibiting the MAPK signaling pathway, R1-5C effectively suppresses leukemic cell growth and offers a therapeutic option for treating ERK-dependent cancers (Table 4).^500^

#### PROTAC targeting p53

The p53 gene is a critical anticancer gene promoting DNA repair and regulated cell death of abnormal cells, preventing cancer development and metastasis. Unfortunately, p53 mutations are prevalent in numerous cancers, with up to 50% of malignancies harboring p53 mutations. Due to the absence of a typical drug target, no approved p53-based therapies exist globally. MDM2, a negative regulator of p53, has been targeted using MDM2 inhibitors, but these have not demonstrated significant therapeutic effects. PROTACs based on MDM2 inhibitors hold potential as a treatment option for p53-driven cancers.^501,502^

In 2018, Wang’s team successfully developed PROTAC molecules degrading MDM2, utilizing the MDM2 inhibitor MI-1061 and the CRBN ligands thalidomide and lenalidomide. MD-224 (**208**) proved to be a highly effective MDM2 degrader, capable of rapidly degrading MDM2 at concentrations below 1 nM in human leukemia cells (Table 4). Results indicated that MD-224 was more potent in inhibiting growth and inducing apoptosis in p53 wild-type leukemia cells compared to the inhibitor MI-1061. A single dose of MD-224 induced MDM2 degradation and p53 activation in RS4;11 leukemia xenograft tissues, leading to complete and long-lasting tumor regression in a mouse model at well-tolerated doses. Consequently, MD-224 is a promising and highly efficient MDM2 degrader with potential for treating human leukemia and other cancers.^54^

Subsequently, the same group discovered a novel compound, MG-277 (**209**), targeting MDM2 for degradation while also acting as a “molecular glue” to facilitate the degradation of an additional target protein, GSPT1 (Table 4). Their study demonstrated that the designed phthalimide-conjugated compound functioned effectively as both a true degrader of the target protein and a molecular glue, recruiting new substrate proteins into the E3 ligase for ubiquitination and degradation.^503^

In 2021, Wang’s team designed and synthesized a series of PROTACs based on ursolic acid (UA) and thalidomide. They discovered that compound **210**, linked to POE-3 (3-polyoxyether), effectively inhibited the growth of various cancer cells (Table 4). Further analysis using Western blotting demonstrated that compound **210** substantially degraded MDM2 and increased the expression of p21 and PUMA proteins, thereby suppressing cell proliferation and promoting apoptosis in A549 cells. Employing the natural compound UA to target MDM2 presents a novel avenue for MDM2-targeted therapies.^504^

Concurrently, Sheng’s team designed and synthesized a PROTAC molecule, compound **211**, targeting MDM2 degradation (Table 4). This molecule was based on the MDM2 inhibitor Nutlin-3. Experimental results revealed that PROTAC **211** effectively dimerized MDM2 with high binding activity and induced its degradation in A549 NSCLC cells. The study also demonstrated that compound **211**, which has a chiral center, and its enantiomeric isomer **211-1** exhibited stronger antiproliferative abilities than PROTAC **211** in tumor cells and displayed potent in vivo antitumor activity in an A549 xenograft nude mouse model.^505^

In 2018, Wang’s team designed and synthesized PROTAC molecules using the MDM2 inhibitor MI-1061, which not only exhibited significant degradation of leukocytes but also markedly inhibited tumor growth in a mouse model.^506^ Subsequently, Tang’s team developed WB156 (**212**), a highly efficient MDM2-PROTAC comprising a nutlin derivative linked to the CRBN ligand lenalidomide (Table 4). In leukemic cells, WB156 effectively depleted MDM2 and activated wild-type p53, inducing apoptosis; however, it was only effective in a limited number of leukemic cell lines.^507^ After further optimization, the team identified WB214 (**213**) as the most potent antiproliferative agent across various leukemic cell lines (Table 4). Unlike WB156, WB214 does not activate p53 but instead induces its degradation, and subsequent investigations revealed that its mechanism of action more closely resembles that of a molecular glue.^508^

#### PROTAC targeting TFs

TFs are proteins that bind to specific DNA sequences to regulate gene expression, determining cell types and controlling developmental patterns. They play crucial roles in various human diseases, including cancer. However, targeting TFs with small molecule drugs (SMDs) has been challenging due to the lack of prominent binding sites on these proteins. Unlike kinases, direct regulation of TFs necessitates modulating both protein–protein and protein–DNA interactions, which proves difficult due to the flat surface and absence of binding pockets on these proteins, as well as the highly positively charged nature of DNA.^22,509^ TPD technology offers a solution to these challenges and holds the potential to target “undruggable” substances without prominent binding sites.

Signal transducer and activator of transcription 3 (STAT3), a member of the STAT family, plays a critical role in transmitting extracellular signals to the nucleus, governing inflammation, combating viral infections, and regulating antitumor immune responses. Overexpression of STAT3 can result in the development of various diseases. Inhibiting the formation of STAT3-STAT3 dimers by targeting the SH2 domain is currently the primary approach for antitumor therapy. However, the SH2 domain is highly conserved, making it difficult to find specific small molecule inhibitors for STAT3, leading to STAT3 classification as an “undruggable” protein.^510^ Recently, Kymera advanced its STAT3 degrader, KT-333 (**214**), into clinical trials for the treatment of hematologic malignancies and solid tumors (Table 4). KT-333 is a potent and selective bifunctional small molecule protein degrader targeting the STAT3 protein.^511^ Similarly, Wang’s team developed a small molecule degradation agent for STAT3, known as SD-36 (**215**), to overcome the challenges faced in targeting this protein (Table 4). SD-36 effectively degraded STAT3 protein in both xenograft tumor tissue and normal mouse tissue, leading to complete and durable tumor regression in a xenograft tumor model at well-tolerated doses. The high specificity of SD-36 for STAT3 was demonstrated as it induced rapid degradation of STAT3 at low nanomolar concentrations in cells and did not degrade other STAT proteins.^512^

FoxM1 is a critical TFs that plays a pivotal role in various physiological and pathological processes, including cell proliferation, embryonic development, aging, and tumors. Developing small molecule inhibitors that directly target FoxM1 has proven challenging due to the absence of a well-defined binding site or large interaction interface. However, TPD technology has gained significant attention as it holds the potential to degrade “undruggable” targets like FoxM1.^513^ In 2021, Xiang’s research team pioneered the discovery of the first set of PROTAC degraders targeting FOXM1, including compound **216** (Table 4). Among them, **216** emerged as the most potent FOXM1 degrader, exhibiting a DC_50_ value of 1.96 μM in TNBC MDA-MB-231 cells.^514^ In 2022, Huang’s team introduced a novel FoxM1-PROTAC (**217**) that targets and degrades FoxM1 (Table 4). Utilizing an antagonistic peptide, FIP-1, which comprises a FOXM1-targeting peptide and a cell-penetrating peptide sequence with FoxM1 inhibitory properties, the researchers linked FIP-1 with the E3 ubiquitin ligase ligand pomalidomide to form FoxM1-PROTAC, demonstrating a stronger inhibitory effect on cancer cells than FIP-1 alone.^513^

Comprising C-MYC, MYCN, and MYCL, the MYC oncogene family has been implicated in the development of numerous tumor types. Research indicates that approximately 70% of human cancers involve dysregulated MYC expression. C-Myc, specifically, plays a crucial role in regulating gene expression, cell proliferation, and differentiation.^515^ However, its nuclear location poses challenges for targeting with large molecules. Directly targeting c-Myc with small molecule inhibitors has proven difficult, rendering it an “undruggable” target. Presently, small molecule drug development has shifted focus to targeting the Myc-associated protein X (Max) and c-Myc heterodimer, driven by bromodomain and extraterminal (BET) proteins, particularly bromodomain-containing protein 4 (BRD4).^516,517^

In 2021, Hu’s team revealed, through cellular in vitro experiments, that ARV-825 (**218**) effectively degraded the BRD4 protein while inhibiting proliferation and inducing apoptosis (Table 4). In primary drug-resistant T-cell acute lymphoblastic leukemia (T-ALL) cells, the researchers observed that ARV-825 eradicated cells through BRD4 protein degradation, significantly reducing c-Myc expression.^518^ Crews’s team also developed ARV-771 (**219**), another PROTAC molecule featuring VHL as the E3 ligase ligand, which efficiently degrades BRD4 protein in prostate cancer cells, exhibiting a potency of BRD2/3/4 degradation in various prostate cell lines below 5 nM (Table 4). The researchers also observed that ARV-771 effectively downregulated c-Myc levels.^519^

Moreover, dBET1 (**220**) is a PROTAC engineered with CRBN as an E3 ubiquitin ligase ligand, specifically designed for the treatment of microglia and colorectal cancer (Table 4).^520^ These PROTACs address the challenges small-molecular inhibitors (SMIs) face when attempting to locate active sites in TFs. Researchers discovered that the degradation efficacy of PROTACs is influenced by valence number. The trivalent PROTAC named SIM1, developed by Satomi Imaide’s team, exhibited significantly enhanced degradation compared to its divalent counterpart.^521^

#### PROTAC targeting Cyclin D1

Cyclin D1, a crucial cell cycle regulator, activates CDK4/6 by phosphorylating Rb protein. This activation process results in the release of E2F repression and the induction of proteins that promote the G1 to S phase transition. Cyclin D1 is frequently overexpressed and amplified in various cancer types, such as breast, esophageal, and lung cancers, due to its role in mitogen-dependent cell cycle control. Although Cyclin D1 plays a significant role in cancer, it is largely dispensable for normal physiology, as mice lacking CCND1 can survive with only minor developmental defects. However, the absence of a known small molecule binding pocket and reported inhibitors for Cyclin D1 has rendered it an undruggable target.^522^

In a recent study, Jin’s team proposed an innovative strategy for developing bridging PROTACs and successfully synthesized MS28 (**221**), a PROTAC that selectively degrades Cyclin D1 (Table 4). MS28 consists of a VHL ligand linked to the CDK4/6 ligand palbociclib, and its degradation of Cyclin D1 relies on CDK6, VHL, and the ubiquitinase system. MS28 preferentially degrades Cyclin D1 over other essential cell turnover proteins or CDKs, demonstrating superior antiproliferative effects compared to CDK4/6 inhibitors and degraders.^522^

#### Molecular glues targeting protein degradation

The concept of molecular glues was first introduced in the early 1990s, with immunosuppressants Cyclosporine A (CsA) and FK506 among the initial examples. Although CsA and FK506 have distinct direct receptors, their mechanisms of action are similar, relying on calcium-regulated phosphatase (CaN). Later, Rapamycin and several of its analogs received FDA approval.^478^ Molecular glues are a class of small molecules that promote interaction between E3 ubiquitin ligase, substrate receptors, and target proteins, leading to ubiquitination and degradation by the proteasome. Unlike PROTACs, molecular glues possess a dual-ligand structure for both E3 ubiquitin ligase and target proteins, enabling the ubiquitination and degradation of previously undruggable targets and PPIs. While PROTACs are products of rational design, molecular glues are considered serendipitous discoveries. They feature smaller molecular weights, simpler chemical mechanisms, reduced steric interference, and superior drug formation.^523,524^ However, they cannot be designed through large-scale component screening like PROTACs. The molecular glue approach effectively overcomes the limitations of traditional inhibitors, rendering previously “undruggable” targets “druggable”.

The Ikaros zinc finger (IkZF) gene family, a TF that regulates blood cancer cell differentiation, is significant in the development and progression of leukemia. IkZF1 plays a crucial role, while IkZF3 mutations are vital in the development of malignancy and chronic lymphocytic leukemia. Studies have demonstrated the potential of thalidomide-derivative-based molecular glues to degrade TFs.^525^ Several Bristol Myers Squibb (BMS) compounds have shown promising outcomes in the degradation of specific IKZF factors, including IKZF1 and IKZF3, in preclinical or clinical trials targeting various hematological cancers. Generally, CC-122 (**222**) has demonstrated a favorable safety profile and exhibited anti-cancer activity in clinical trials (Table 4).^526^

CFT7455 (**223**), an innovative molecular glue developed by C4 Therapeutics, targets IKZF1 and IKZF3 and is currently undergoing clinical trials for the treatment of MM and non-Hodgkin’s lymphoma (NHL) (Table 4). This orally bioavailable compound selectively targets IKZF1 (Ikaros) and IKZF3 (Aiolos), causing tumor cell death upon their depletion from malignant B cells and T cell activation when depleted from the tumor microenvironment. In vitro and in vivo models of MM and NHL, including mesenchymal large cell lymphoma and diffuse large B-cell lymphoma, have demonstrated that CFT7455 exhibits higher activity than comparable agents, laying a strong foundation for its efficacy evaluation in the first human phase I study.^527^

Joshua D. Hansen’s research team has developed CC-92480 (**224**), a protein degrader with potential for treating MM (Table 4). Derived from lenalidomide, this therapy binds to the E3 ubiquitin ligase CRBN, leading to the degradation of TFs such as Ikaros and Aiolos. In cell culture assays, the researchers observed that the more efficiently CC-92480 degraded Aiolos, the faster it induced the death of MM cell lines, including those resistant to lenalidomide (LEN) and pomalidomide (POM). The therapy also exhibited potent immunostimulatory activity.^528^

Cyclin K, a recently discovered member of the cyclin family, is thought to play a role in transcriptional regulation, influencing CDK and RNA polymerase II activity. Research indicates that Cyclin K preserves the pluripotency and genomic stability of embryonic stem cells. Its dysregulation is implicated in various cancers, rendering it a promising therapeutic target.^529^ CR8 (**225**), a small molecule, acts as a molecular glue within cells, triggering Cyclin K’s ubiquitination and proteasomal degradation (Table 4). Unlike conventional molecular glues, CR8 bypasses classical E3 ubiquitin ligase substrate receptors like CRBN and DCAF15, binding directly to CDK12. This CDK12-DDB1 interaction results in Cyclin K’s ubiquitination and degradation, offering a unique means of controlling its activity.^530^

Han’s team identified HQ461 (**226**) as another molecular glue with therapeutic potential. The in vitro experiments revealed that HQ461 directly facilitates DDB1’s binding to the CDK12/cyclin K complex, without requiring any other DCAF E3 ligase (Table 4). In essence, this molecular glue operates independently of classical E3 ubiquitin ligases and instead mediates the ubiquitin molecule’s transfer from E2 to the substrate protein Cyclin K via the CUL4 ubiquitin ligase complex backbone. This process culminates in Cyclin K’s polyubiquitination and degradation, positioning HQ461 as a promising candidate for treating Cyclin K-associated cancers.^531^

#### Other proteasome-based TPD technologies for medicinal research

While numerous small molecule-based PROTAC drugs are undergoing clinical trials, researchers continue to explore the potential of PROTACs, resulting in the development of new variants with analogous technologies.

##### p-PROTAC

Peptides, with their unique physicochemical properties, bridge the gap between small and large molecules, enabling access to drug binding sites that small molecule drugs find challenging. This advance transforms previously “undruggable” targets into “druggable” ones and helps mitigate drug resistance associated with small molecule drugs. In 2020, Chen’s team reported the use of peptide-based PROTACs for TPD, introducing xStAx-VHL, a peptide-based PROTAC, to degrade β-catenin. This PROTAC directly recognizes the protein and promotes its degradation via the ubiquitin-proteasome pathway, inhibiting Wnt signaling at the cellular level and demonstrating oncogenic effects in various mouse tumor models and colon cancer patients.^532^ Qu’s team developed another peptide-based PROTAC, CPD-PBD-PTM, targeting the α-synuclein protein, which is linked to Parkinson’s syndrome. The permeable CPD-PBD-PTM peptide promotes α-syn-specific degradation in SK-N-SH cells, alleviating reduced cellular activity and increased toxicity caused by α-syn overexpression. These studies underscore the potential benefits of peptide-based PROTACs (p-PROTACs) for TPD, particularly for challenging targets.^533^

##### PROTAB

The innovative proteolysis-targeting antibodies (PROTABs) protein degradation technology platform utilizes a bispecific antibody approach. It comprises a binding domain (anti-tag) for ubiquitin ligases targeting transmembrane E3 ligases, such as RNF43 or ZNRF3, and a binding domain (anti-POI) for the target protein. In one study, PROTAB effectively degraded the insulin-like growth factor 1 receptor (IGF1R) in rectal cancer patients while causing minimal degradation in normal organs. The researchers also demonstrated the platform’s potential by degrading Her2 and PD-L1 targets. The results showed specific degradation of the targets and successful replication of PROTAB’s degradation mechanism across multiple targets. Although promising, PROTAB is still in its early stages and has not been tested on “undruggable” targets.^534^

##### CHAMP

Chaperone-mediated protein degradation (CHAMP) represents another proteasome-based protein degradation technique that employs molecular chaperones. CHAMP’s structure resembles that of PROTAC, featuring a targeting protein ligand at one end and a molecular chaperone at the other, connected by a suitable linker. Like PROTAC, CHAMP facilitates the ubiquitination of the target protein and its subsequent degradation by the proteasome.^535^ Despite its potential, CHAMP has not yet been applied to regulate “undruggable” targets.

### Lysosome-based TPD

The field of protein degradation through the lysosomal pathway has seen significant progress, complementing advancements in PROTAC-based protein degradation. The lysosomal and proteasomal systems, as the two primary intracellular protein degradation pathways, possess distinct functions. The proteasome system primarily degrades short-lived, soluble, and misfolded monomeric proteins, while the lysosomal system focuses on long-lived proteins, protein aggregates, and damaged organelles. Both systems can achieve substrate degradation through ubiquitination and can also collaborate to degrade the same intracellular substrate. Utilizing the lysosomal pathway for protein degradation allows targeting a broader range of substrates, including traditionally non-targetable proteins.^536^ This field currently encompasses two primary classes of technologies: autophagy-lysosome pathways (such as AUTACs, ATTECs, and AUTOTACs) and endocytosis-lysosome pathways (such as LYTACs and MoDE-As). Among these, ATTEC technology holds the most promise for clinical translation.

Lysosomal protein degradation is primarily applied to neurodegenerative diseases, which cause nerve cell damage and result in motor or cognitive dysfunction in patients. These conditions are closely associated with insoluble aggregates formed by protein misfolding.^537,538^ Traditionally considered “undruggable” by inhibitors and agonists/antagonists, these aggregates now present potential therapeutic targets through emerging protein degradation technology.

#### ATTEC targeting mHTT and UPS30

In 2019, Lu et al. proposed a direct strategy for the selective degradation of mHTT using autophagosome-tethering compound (ATTECs). Small-molecule microarray-based screening identified four compounds (10O5 (**227**), AN1 (**228**), AN2 (**229**), and 8F20 (**230**)) that interacted with LC3 and mHTT, leading to degradation (Table 4). The polyglutamine (polyQ) region in mHTT, which differs from wild-type huntingtin (wtHTT), offers a target site for selective degradation. These small molecules may target the polyQ region, demonstrating better selectivity and lower maximal degradation (Dmax) rates than mHTT-PROTAC. Concentration-dependent mHTT degradation without affecting wtHTT highlights the feasibility of TPD for HD therapy. Autophagy inhibitors, such as NH_4_Cl or chloroquine, blocked the mHTT-lowering effects of ATTEC, indicating small-molecule compounds’ involvement in the autophagy machinery for selective degradation. Blood-brain barrier (BBB) permeability and mHTT-lowering effects after peripheral injection of these compounds offer potential for drug discovery in treating HD and other polyQ-related diseases.^539^

Parkinson’s disease (PD) is a debilitating disorder primarily affecting the gastrointestinal tract and autonomic nervous system, resulting in progressive motor delays and tremors that intensify with age. Aberrant expression of α-synuclein and other proteins, coupled with mitochondrial damage, has been implicated in PD. Deubiquitinase ubiquitin-specific protease 30 (USP30), an enzyme situated on the outer mitochondrial membrane, functions as a negative regulator of mitochondrial autophagy, mitigating the excessive accumulation of dysfunctional mitochondria, thus presenting a potential therapeutic target for PD treatment. In 2021, Li et al. reported a novel peptide (Q14) (**231**) capable of traversing the cell membrane and binding to mitochondria-anchored USP30, inhibiting deubiquitination and degradation of mitochondrial proteins (Table 4). Mechanistic investigations revealed that Q14 variably modulated the USP30 fingers domain to suppress enzymatic activity. Furthermore, Q14 directly bound LC3 via its LC3 interaction region (LIR) domain, connecting the USP30 protein and mitochondria to the autophagic membrane in an ATTEC-dependent manner, thereby enhancing autophagic vesicle formation and synergistically promoting mitochondrial autophagy. This study substantiates the feasibility of targeting USP30 and establishes a foundation for developing bifunctional ATTEC molecules that may herald a breakthrough in PD treatment.^540^

#### AUTOTAC targeting Tau

Alzheimer’s disease (AD) is a multifaceted neurological disorder predominantly affecting the elderly population, characterized by diverse etiologies. The most direct and practical strategies for AD treatment entail degrading Aβ and Tau aggregates through autophagy. The AUTOphagy-TArgeting Chimera (AUTOTAC) platform, devised by Kwon et al., demonstrates more potent and selective degradation of mutant Tau protein compared to PROTAC mechanisms. AUTOTAC PBA-1105 (**232**) provokes autophagic degradation of TauP310L with remarkable efficacy at approximately 1–10 nM DC_50_ (Table 4). In a transgenic mouse model treated with 20 or 50 mg kg^−1^ PBA-1105 injections thrice weekly for one month, significant clearance of TauP301L aggregates was observed, indicating that blood-brain barrier penetration was not a constraint. Coimmunoprecipitation experiments unveiled that the ubiquitin-associated domain and recognition were not essential for AUTOTAC PBA-1105, differentiating its mode of action from that of AUTAC, which will be discussed in the subsequent subsection.^541^

#### Other lysosome-based TPD technologies for medicinal research

Lysosome-based TPD technologies have exhibited remarkable potential in treating various diseases, especially those considered undruggable. Therefore, Lysosome-based TPD technologies provide an essential technological foundation for overcoming the challenges associated with such elusive targets in the future.

##### AUTAC

Autophagy-targeted chimeras (AUTAC) represent the pioneering technique for harnessing the lysosomal autophagic pathway to degrade target proteins. In 2019, Takahashi et al. developed AUTAC, building upon the understanding that endogenous 8-nitroguanosine 3’,5’-cyclic monophosphate (8-nitro-cGMP) is a vital signaling molecule for cellular recruitment of autophagosomes. The researchers designed a series of AUTAC molecules by connecting target protein or organelle ligands to autophagosome-recruiting tags (guanine derivatives) through linkers. These molecules successfully degraded methionine aminopeptidase 2 (MetAP2), 506-binding protein (FKB12), BRD4 (BET family protein), and other disease-related target proteins. Furthermore, AUTAC4 (mito-AUTAC), which targeted the mitochondrial outer membrane transporter protein ligand 2-phenyl-3-acetonamide, degraded fragmented mitochondria and significantly enhanced mitochondrial activity in Down syndrome fibroblasts, showcasing AUTAC’s considerable potential for treating related diseases.^542^

##### LYTAC

The lysosomal protein degradation pathway extends beyond targeting intracellular protein domains, as the cell surface lysosomal targeting receptor family (LTRs) can transport extracellular proteins to lysosomes for degradation. Consequently, researchers have proposed lysosomal targeting chimera technologies (LYTACs), chimeric molecules that combine cell surface lysosomal targeting receptors and extracellular or membrane proteins, to expand TPD technologies to extracellular targets. Bertozzi’s group at Stanford University developed the first LYTAC molecule, Ab-2, by fusing cetuximab (ctx), a monoclonal antibody targeting EGFR, with an M6P-modified peptide (which binds to CI-M6PR protein). Experimental results showed that Ab-2 significantly reduced EGFR levels in HeLa cells. Since this development, LYTAC molecules have been applied to degrade a wider array of extracellular targets, including HER2, ApoE4, PD-L1, and others.^543,544^

##### MoDE-As

MoDE-As, or molecular degraders that degrade extracellular proteins via the asialoglycoprotein receptor (ASGPR), represent a novel extracellular protein degradation technology initially reported by Professor David A. Spiegel of Yale University. MoDE-As comprise three GalNAc ligands recognized by ASGPR, a protein-of-interest (POI) binding element, and a polyethylene glycol (PEG) spacer fragment linking the two components. In vivo and in vitro results demonstrated that MoDE-A molecules facilitated the rapid clearance of α-DNP antibodies or cytokine MIF from circulation, suggesting the platform’s potential for TPD of therapeutically relevant proteins in vivo. Notably, this is the first molecule shown to effectively mediate extracellular protein degradation both in vivo and in vitro.^545^

##### KineTAC

To address the challenge of targeting soluble proteins outside cells, James A. Wells’ team developed cytokine receptor-targeting chimeras (KineTACs), a modular cytokine receptor targeting chimera for the targeted degradation of cell surface and extracellular proteins. KineTAC is a fully recombinant bispecific antibody based on a human scaffold that employs the internalization of homologous receptors through endogenous cytokine-mediated pathways for efficient delivery of cell surface and extracellular proteins to the intracellular environment. The N-terminal end of human CXCL12 chemokine is fused with the Knob Fc structural domain, while the second arm contains the antigen-binding fragment (Fab) antibody sequence of Atz, an FDA-approved PD-L1 inhibitor. In a proof-of-concept demonstration, the authors generated a KineTAC CXCL12-Atz targeting PD-L1, which effectively degraded PD-L1 in breast cancer cells after 24 h of treatment. In contrast, single-arm Atz Fab or CXCL12 alone failed to induce PD-L1 degradation, emphasizing the dependence of PD-L1 degradation on the bispecific KineTAC scaffold. The KineTAC platform also effectively degraded various membrane proteins, including HER2 and EGFR, demonstrating its versatility for degrading different cell surface proteins. However, no studies have been conducted on undruggable targets.^546^

##### NanoTAC

Rebecca Feltham and her team developed a novel heterobifunctional protein degrader that targets substrate proteins by binding to NanoLuc, a luminescent tag. NanoTACs are NanoLuc inhibitors bound to E3 ligase ligands, using the complex to trigger the degradation of NanoLuc-labeled POIs. The group synthesized a series of NanoLuc-CRBN (NC) targeting NanoLuc using the E3 ligase ligand CRBN. Treatment with NanoTAC led to the degradation of cells expressing the H-FF-N-F fusion protein, while the control compound NC* had no effect. Degradation efficiency was concentration-dependent, with NC4 exhibiting the most effective degradation capacity. However, a hook effect was observed at higher concentrations, where degradation was prevented due to the inability of the ligand to form ternary complexes. NanoTACs effectively recruited the CRL4CRBNE3 ligase complex, leading to the degradation of NanoLuc-tagged substrate proteins. Furthermore, NanoTACs acted as catalytic degradation catalysts. Yet, no studies have been conducted on undruggable targets.^547^

### Targeted protein stabilization (TPS)

TPD technology is a powerful tool that addresses the issue of blocked target protein degradation. While TPD has been shown to be effective in some disease treatments, excessive protein degradation can be detrimental. Therefore, targeted protein stabilizing drugs are urgently needed to counteract excessive protein degradation.

#### DUBTAC

Nomura’s team, inspired by PROTAC, developed deubiquitinase-targeting chimeras (DUBTACs) for TPS. They identified EN523, a molecule that selectively binds to the OTUB1 enzyme, and combined it with lumacaftor to create NJH-2-057. This DUBTAC effectively inhibits ΔF508-CFTR degradation, increasing protein levels and demonstrating improved stabilization compared to lumacaftor alone in cystic fibrosis donor cells.^548^ Building on this foundation, Wei’s team developed a TF-DUBTAC platform capable of selectively stabilizing oncoproteins, including FOXO3A, p53, and IRF3. They chose FOXO-specific DNA motifs as binding ligands and incorporated an azide group at the 5’ end of the DNA motifs. Utilizing the Click reaction, they linked EN523-BCN and N3-FOXO-ODN to generate TF-DUBTACs. After evaluating the activity of each compound, the authors selected two molecules for further investigation. They discovered that compound **233** significantly upregulated FOXO3A in a concentration-dependent manner, exhibiting selective stabilization of FOXO3A compared to the structurally similar FOX1 within the same family (Table 4). Unbiased mass spectrometry analysis revealed that compound 6 efficiently and specifically stabilized FOXO3A while inhibiting Myc expression, demonstrating its potential as a promising therapeutic approach.^549^

## Nucleic acid-based approach

**Fig. 6:**
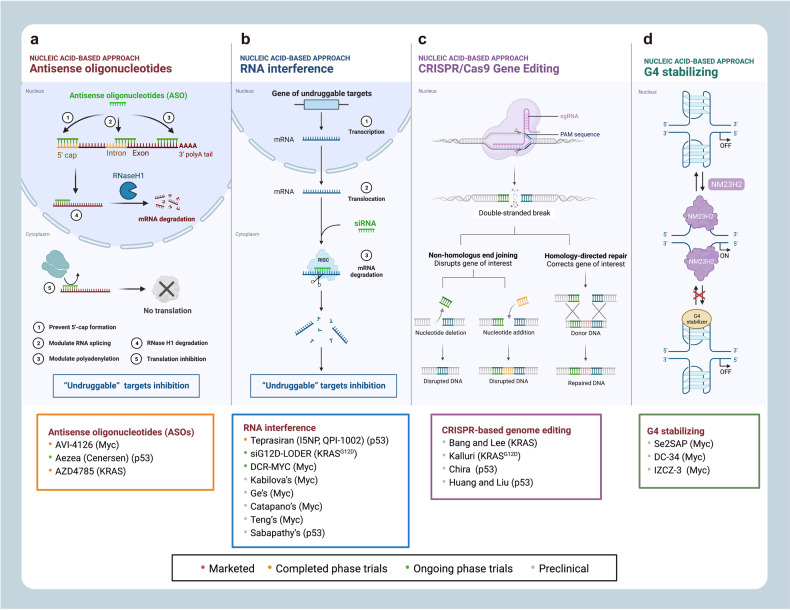
Nucleic acid-based approaches and cases targeting undruggable proteins. **a** Antisense oligonucleotides (ASOs) inhibit translation of RNAs thereby targeting undruggable proteins. **b** RNA interference (RNAi) triggers the degradation of particular RNAs thereby down-regulating undruggable proteins. **c** CRISPR/Cas9 edits mutant oncogenic allele thereby targeting undruggable proteins. **d** G-quadruplex (G4) stabilizer regulates c-Myc transcription thereby targeting the undruggable Myc

With the increasing recognition of the vital role played by RNA in transferring cellular information and regulating genes, targeting RNAs has become an exciting opportunity to therapeutically modulate cellular processes linked to previously “undruggable” protein targets.^550–552^ RNA-based therapeutics have proven to be a stunning approach to regulate “undruggable” proteins at the genetic/transcription level, driven by the Watson-Crick complementary rule of binding, which has broadened the range of druggable targets.^553^ Since it was first proposed in the 1970s, RNA-based therapeutics, also known as oligonucleotide therapeutics, have been well-developed and classified into the following types: antisense oligonucleotides (ASOs), RNA interference (RNAi) including small interference RNAs (siRNAs) and microRNAs (miRNAs), CRISPR-based genome editing, and G4 stabilizing. The first two types have emerged as the most representative for clinical development and therapeutic application. In terms of undruggable proteins, siRNA molecules occupy a prominent position in drug discovery (Table 5 and Fig. 6).

**Table 5:** Compounds targeting undruggable proteins through nucleic acid-based approach

Compound name and structure	Target	Cancer cell line (activity)	Indications	Status/clinical trial identifier	Ref.
Antisense oligonucleotides (ASOs)					
AVI-4126 (**234**)^a^	Myc	LNCaP, PC3, DU 145	Prostate tumor, lung metastasis	*Completed* NCT00343148(I)	^554^
Aezea (Cenersen) (**235**)^a^	P53	KASUMI-1, MV4-11, K562	Myelodysplastic syndromes, lymphoma, acute myelogenous leukemia	*Completed* NCT00074737(II) *Withdrawn* NCT00967512(II), *Terminated* NCT02243124(I), NCT00636155(II)	^557^
AZD4785 (**236**)^a^	KRAS	NCl-H1299, NCl-H1793, Colo201	Advanced solid tumors	*Completed* NCT03101839(I)	^564^
RNA interference					
Teprasiran (I5NP, QPI-1002) (**237**)^a^	P53	–	Kidney acute renal failure	*Completed* NCT02610296(III) *Terminated* NCT00683553(I), NCT03510897(III)	^570^
siG12D-LODER (**238**)^a^	KRAS^G12D^	–	Advanced pancreatic cancer	*Completed* NCT01188785(I) *Ongoing* NCT01676259(II)	^571^
DCR-MYC (**239**)^a^	Myc	–	Hepatocellular carcinoma	*Terminated* NCT02110563(I), NCT02314052(I/II)	^572^
Sabapathy’s research p53-specific siRNAs Compound (**240**)^a^	P53	RD, PLC-PRF5, H1975	–	*Preclinical*	^574^
Kabilova’s research Compound (**241**)^a^	Myc	KB-3-1	–	*Preclinical*	^575^
Ge’s research Compound (**242**)^a^	Myc	HT-29	–	*Preclinical*	^576^
Catapano’s research Compound (**243**)^a^	Myc	human PC3	–	*Preclinical*	^577^
Teng’s research Compound (**244**)^a^	Myc	SKOV3	–	*Preclinical*	^578^
CRISPR-based genome editing					
Bang and Lee’s research Compound (**245**)^a^	KRAS	SW620, SW480, AsPC-1, SNU497	–	*Preclinical*	^579^
Kalluri’s research Compound (**246**)^a^	KRAS^G12D^	KPC689	–	*Preclinical*	^580^
Huang and Liu’s research Compound (**247**)^a^	P53	p53 knockout cell		*Preclinical*	^582^
G4 stabilizing					
Se2SAP (**248**)	Myc	Hela	–	*Preclinical*	^583^
DC-34 (**249**)	Myc	L363 (IC_50_ = 3.5 μM)	–	*Preclinical*	^584^
IZCZ-3 (**250**)	Myc	SiHa (IC_50_ = 3.3 μM), HeLa (IC_50_ = 2.1 μM), Huh7 (IC_50_ = 4.1 μM), A375 (IC_50_ = 4.2 μM), BJ (IC_50_ = 15.9 μM), mesangial cells (IC_50_ = 15.6 μM)	–	*Preclinical*	^585^

Data collected from https://clinicaltrials.gov [last accessed March 2023]

^a^The chemical formula was not disclosed

### Antisense oligonucleotides (ASOs)

ASOs are short synthetic single-stranded oligonucleotides ranging in size from 12 to 30 nucleotides that can inhibit gene expression and modulate splicing through the classic Watson-Crick base pairing. Despite the approval of oligonucleotide therapeutics for the treatment of rare diseases, none of the approved ASOs are related to undruggable proteins.

As early as 1997, Laxmanan’s group verified c-Myc antisense oligonucleotide (c-Myc-ASO) induces cell death in three kinds of human prostate cancer cell lines, LNCaP, PC3, and DU 145, by decreasing DNA synthesis and cell viability.^554^ AVI BioPharma Inc. developed a novel antisense phosphorodiamidate morpholino oligomer (PMO), AVI-4126 (**234**), that can inhibit prostate tumor growth and lung metastasis both in vitro and in vivo by targeting and inhibiting c-Myc translation. Its safety was demonstrated in a single center, open label, and dose-escalating phase I clinical trial (NCT00343148) involving healthy volunteers with i.v. administration.^555,556^

Aezea (Cenersen) (**235**) is a 20-mer phosphorothioate ASO with a specific nucleotide sequence 5′-d[P-Thio] (CCCTG CTCCC CCCTG GCTCC)- 3′ that acts on TP53, which encodes for the undruggable tumor protein p53.^557^ Cenersen has entered phase I clinical trials for treatment of myelodysplastic syndromes (NCT02243124) and phase II for lymphoma and acute myelogenous leukemia (NCT00967512, NCT00074737, NCT00636155), either alone or in combination with chemotherapy. Unfortunately, few desired results have been obtained in clinical trials, and no further advancement has been made since 2012.^558–560^

Numerous modified antisense oligonucleotides, varying in size, have been reported to exhibit inhibitory activities on various cancer cell lines in vitro and in vivo by targeting specific mutated *RAS* genes.^561–563^ Among them, AZD4785 (**236**) is a high-affinity constrained ethyl-modified ASO that has shown potential activity in downregulating KRAS mRNA by complementing the KRAS mRNA sequence both in vitro and in vivo. The advanced chemistry of AZD4785 allows it to achieve this without any delivery agent.^564^ The ASO was developed by Ionis in collaboration with Astra Zeneca and has undergone only one phase I clinical trial sponsored by Astra Zeneca. This clinical trial was first posted in 2017 and updated in 2019 for a dose-escalation study in patients with advanced solid tumors. However, no results have been posted yet (NCT03101839).

### RNA interference

RNA interference (RNAi) is an endogenous cellular process that triggers the degradation of particular RNA targets by double-stranded RNAs, result in the subsequent down-regulation of corresponding proteins.^565–567^ RNAi-based therapy is the most approach for targeting undruggable proteins at the genetic/transcription level. Short double-stranded RNAs (20-24 nt), called siRNAs, with distinct structures containing 5’-phosphate/3’-hydroxyl endings and two 3’-overhang ribonucleotides on each duplex strand, are used for RNAi-based therapies in their fancy.^568,569^ Despite of this, the rapid advances in preclinical and clinical studies may encourage further exploration of siRNA for undruggable disease treatment.

I5NP (QPI-1002) (**237**), also known as teprasiran, is an siRNA that targets mutant-specific p53 developed by Quark Pharmaceuticals for the treatment of kidney acute renal failure (NCT00683553).^570^ It has entered the phase III clinical trials to prevent major adverse kidney events in subjects at high risk for acute kidney injury following cardiac surgery (NCT03510897) and to prevent delayed graft function in recipients of older donor kidney transplant (NCT02610296).

siG12D-LODER (**238**) is a mutant-specific siRNA-polymeric nanoparticles against KRAS^G12D^ developed by Silenseed Ltd. to treat advanced pancreatic cancer. Preclinical trials showed that KRAS^G12D^ suppressed the growth of pancreatic tumors in rat models with local and systemic safety and tolerability.^571^ As expected, it has entered a phase II clinical trial in combination with chemotherapy treatment (NCT01676259), following the completion of a previous phase I clinical trial reporting an enhanced therapeutic effect (NCT01188785).

Dicerna Pharmaceuticals has developed DCR-MYC (**239**), a siRNA lipid-based nanoparticle for the treatment of various cancers such as solid tumors, non-Hodgkin’s lymphoma, MM, pancreatic neuroendocrine tumors (PNET), Non-Hodgkin Lymphoma (NHL) and hepatocellular carcinoma (HCC), using EnCore Dicerna’s proprietary technology for liposomal delivery.^572^ Although the initial clinical trial showed promising clinical and metabolic responses at various dose levels, all clinical trials (NCT02110563, NCT02314052) were terminated on Dicerna Pharmaceuticals decision due to the lack of gene-silencing effectiveness.

Besides the aforementioned siRNAs that target undruggable proteins in clinical trials, there are also mutation-specific siRNAs capable of selectively silencing the expression of intended mutant gene forms. In 2002, a single base difference was found between wtp53 and mutp53, demonstrating that synthetic siRNAs are efficient tools to inactive oncogenic mutations and restore p53 pathways.^573^ In 2019, Kanaga Sabapathy and colleagues designed a series of siRNAs that selectively target four p53 hot-spot mutants. Some of these mutant p53-specific siRNAs have been shown to elevate the dominant-negative activity of mutant p53 over the wild-type form, such as compound (**240**), thus sensitize tumor cells to therapeutic treatment, abrogating the addiction of tumor cells to mutant p53 for survival, promoting cell death of cancer cells expressing mutant p53, and retarding tumor growth in patient-derived xenografts without any side effects or organ toxicity. These findings highlight the enormous therapeutic potential that can be further enhanced in combination with other chemotherapeutic agents or radiotherapy.^574^

In 2006, Kabilova and colleagues synthesized siRNAs, compound (**241**), targeting different specific regions of the c-Myc mRNA using both enzymatic and chemical methods. Both types of synthesized siRNAs were effective in reducing c-Myc mRNA levels in KB-3-1 human epidermoid carcinoma cells, leading to inhibition of cell proliferation.^575^ In 2009, Ge’s group obtained a c-Myc siRNA, compound (**242**), through in vitro transcription and transfected it into HT-29 cells. This resulted in down-regulation of c-Myc expression, inhibition of cell proliferation, and induction of apoptosis in vitro. Furthermore, the growth of colon cancer cells was suppressed in vivo.^576^ Catapano’s group later demonstrated that a promoter-targeted siRNA, compound (**243**), could inhibit transcription of the c-Myc gene, leading to growth arrest and cell senescence of human PC3 cells.^577^ In 2013, Teng’s group designed and synthesized a c-Myc-siRNA, compound (**244**), and transfected it into SKOV3 ovarian carcinoma cell lines, demonstrating its potential in cancer therapy. The siRNA inhibited cell growth and proliferation, and down-regulated the expression of c-Myc mRNA and protein through oncogene silencing.^578^

### CRISPR-based genome editing

CRISPR/Cas9 is a cutting-edge technology that provides a promising avenue for therapeutic gene editing to treat complex diseases and target undruggable proteins. While researchers have attempted to target undruggable proteins such as KRAS and p53 using gene-editing techniques, the application of CRISPR/Cas9 for this purpose are largely theoretical.

In 2017, Bang and Lee’s group successfully designed single-guide RNAs, compound (**245**), to selectively target a single-nucleotide substitution by CRISPR/Cas9 on codon-12 of KRAS in CRC cell lines. By disrupting the oncogenic alleles, cancer cell growth was inhibited, indicating that KRAS mutant-specific CRISPR/Cas9-mediated genome editing could potentially be adopted for cancer therapy.^579^

In 2021, Kalluri and co-workers disclosed that engineered exosomes could serve as natural cell-derived nanocarriers and be developed as an alternative, nonviral delivery system for CRISPR/Cas9. They applied these exosomes, like compound (**246**), loaded with CRISPR/Cas9 to target the mutant KRAS^G12D^ oncogenic allele in pancreatic cancer cells, suppressing cell proliferation and inhibiting tumor growth in vivo. Thus, exosomes provide an optional platform for CRISPR/Cas9 gene editing to target undruggable KRAS protein.^580^

In 2019, Chira’s group proposed an innovative idea for the highly tumor-specific delivery of TP53 to replace the mutant TP53 gene with a functional copy in tumor cells, resulting in sustained expression of the undruggable p53 protein and tumor regression.^581^ In the meantime, Huang and Liu’s group constructed a p53 genetic sensor compound (**247**), and combined it with diphtheria toxin using the CRISPR-Cas9 system to specifically eliminate p53-deficient cells. The sensor selectively killed p53-deficient tumor cells by becoming activated in the presence of WT p53, while protecting normal cells from diphtheria toxin.^582^

### G4 stabilizing

G-quadruplexes (G4s) are noncanonical DNA structures that play essential roles in cellular processes such as the transcriptional control of c-Myc. As such, they are attractive therapeutic targets, providing a promising strategy for drug discovery to target undruggable Myc by recognizing, targeting and stabilizing G4s. In 2005, Hurley’s group designed and synthesized Se2SAP (5,10,15,20-[tetra(N-methyl-3-pyridyl)]-26,28-diselenasapphyrin chloride) (**248**), a selenium-substituted expanded porphyrin analog based on the modeling and comparative analysis of the binding of a G4 stabilizer TMPyP4 and a natural product telomestatin to c-Myc G4. Se2SAP selectively binds with the c-Myc G4 in the presence of duplex DNA and other G4s, offering a potential approach to selectively target undruggable Myc.^583^ In 2018, Walters, Mock and Schneekloth Jr’s group synthesized a library of drug-like compounds and reported a small molecule, named DC-34 (**249**), which showed impressive inhibitory activity on Myc at the transcriptional level only when a G4 is present in the promoter.^584^ In the same year, Chen and Tan’s group reported a novel four-leaf clover-like ligand, called IZCZ-3 (**250**), through structural modification of aryl-substituted imidazole/carbazole conjugates, which binds and stabilizes the c-Myc G4 at the molecular level. IZCZ-3 inhibits cell growth by inducing cell cycle arrest and apoptosis, and suppresses tumor growth in a mouse xenograft model, providing a promising avenue for drug discovery targeting undruggable Myc.^585^

## Immunotherapy

**Fig. 7:**
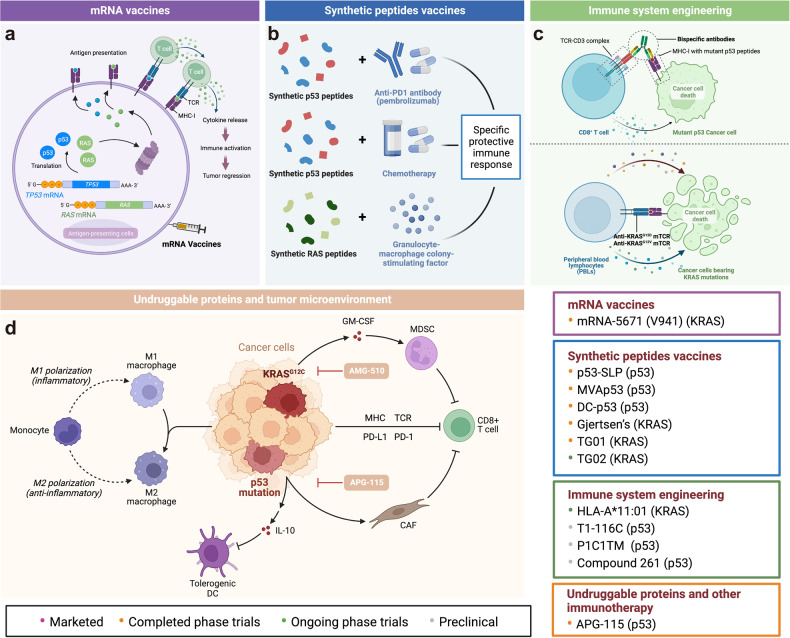
Immunotherapy approaches and cases targeting undruggable proteins. **a** mRNA vaccines targeting p53 and KRAS. **b** Synthetic peptides vaccines targeting p53 and KRAS. **c** Immune system engineering targeting p53 and KRAS includes adoptive cell therapy and T cell receptor (TCR)-like antibodies. **d** Undruggable protein-related gene mutations lead to a cancer-supportive tumor immune microenvironment (TIME)

The immune system plays a crucial role in regulating disease progression and has demonstrated outstanding success in treating several types of cancer through immunotherapy. Therefore, immunotherapy has gained considerable attention in recent years, as it holds potential in treating miscellaneous diseases caused by undruggable proteins. A wide range of immunotherapies, such as immune checkpoint inhibitors, vaccines, specific antibodies, and adoptive cell therapy, have been employed to improve the management of diseases associated with p53 and RAS (Table 6). These advancements offer great promise for the future of disease treatment and highlight the importance of continued research in this field (Fig. 7).

**Table 6:** Immunotherapies targeting undruggable proteins

Compound name and structure	Target	Cancer cell line (activity)	Indications	Status/clinical trial identifier	Ref.
Vaccines					
mRNA-5671 (V941) (**251**)^a^	KRAS	–	Colorectal cancer, NSCLC, pancreas tumor	*Completed* NCT03948763(I)	^587^
p53-SLP (**252**)^a^	P53	PBMC	Colorectal cancer	*Completed* NCT01639885(I/II)	^590^
MVAp53 (**253**)^a^	P53	–	Refractory gastrointestinal cancer, ovarian cancer	*Completed* NCT02275039(I) *Ongoing* NCT02432963(I), NCT03113487(II)	^591^
DC-p53 (**254**)^a^	P53	–	Lung cancer	*Completed* NCT01042535(I/II) *Ongoing* NCT03406715(II)	^596^
Gjertsen’s research Compound (**255**)^a^	KRAS	–	Adenocarcinoma	*Completed* CTN RAS 95002(I/II), CTN RAS 97004(I/II)	^598^
TG01 (**256**)^a^	KRAS	–	Adenocarcinoma	*Completed* NCT02261714(I/II), NCT00569114(I) *Ongoing* NCT05638698(II)	^600^
TG02 (**257**)	KRAS	MV4-11, HL-60	Colorectal cancer	*Withdrawn* NCT03738111(I) *Completed* NCT02942264(I/II), etc. *Terminated* NCT02933944(I)	^601^
Immune system engineering					
HLA-A*11:01 (**258**)^a^	KRAS	–	Colorectal cancer, lung cancer, pancreas cancer	*Ongoing* NCT03745326(I/II), NCT03190941(I/II)	^606^
T1-116C (**259**)^a^	P53	A2058, AU565, CALU6, COR-L23, G361, Hs-695T, MDA-MB-231, NCI-H1299, NCI-H1395, NCI-H1930, NCI-H1975, NCI-H2087, PANC-1	–	*Preclinical*	^607^
P1C1TM (**260**)^a^	P53	SaoS2, HepG2, HT29	–	*Preclinical*	^608^
Zhou’s research Compound (**261**)^a^	P53	–	–	*Preclinical*	^609^

Data collected from https://clinicaltrials.gov [last accessed March 2023]

^a^The chemical formula was not disclosed

### Vaccines

The role of mRNA vaccination is paramount during the ongoing coronavirus disease 2019 (COVID-19) pandemic, heralding a new era for mRNA vaccines’ application in immunotherapy for various challenging diseases. As a matter of fact, several studies have already demonstrated the potency of mRNA in targeting undruggable proteins. As early as 2010, Met’s group determined the immune response of p53 mRNA-transfected dendritic cells in patients with primary breast cancer. Compared to healthy donors and patients with breast cancer expressing p53 at low levels, over 70% of patients with breast cancer expressing p53 at high levels displayed a robust p53-specific interferon gamma (IFNγ) T cell response in vitro, indicating promising pre-existent immunity and paving the way for clinical development of dendritic cell (DC)-based cancer vaccines.^586^ mRNA-5671 (**251**), also known as V941, is a nanoparticle-formulated mRNA vaccine taken up by antigen-presenting cells and presented on the cell surface after translation, leading to T cell responses to the mutant RAS neo-epitopes.^587^ Merck Sharp and Dohme LLC has sponsored a phase I clinical trial (NCT03948763) to assess its safety and tolerability as monotherapy and in combination with pembrolizumab in patients with KRAS mutant advanced or metastatic NSCLC, colorectal cancer or pancreatic adenocarcinoma. Although the trial’s results are not yet available, this development offers immense hope for treating challenging diseases using mRNA-based immunotherapies.

Synthetic peptides have proven to be a valuable tool for investigating the binding capacities, proteolytic activities and immunogenicity properties of proteins, enabling the development of vaccine candidates that can create a specific protective immune response by stimulating both cellular and humoral immunity with the B and T cell epitopes.^588^ Since the 1990s, researchers have been developing vaccines to raise cellular immunity against cancer cells that bear excessive amounts of p53 protein. In 2009, Speetjens and co-workers reported that a synthetic peptide vaccine, compound (**252**), comprising 10 overlapping peptides, with a sequence derived from regions of the wtp53 protein that are rarely mutated in cancer, effectively elicited a T cell response predominated by CD4+ T cells in metastatic colorectal cancer, with mild adverse events reported.^589^ However, a subsequent clinical trial conducted by Leffers and co-workers in 2012 failed to show any benefit over historical controls.^590^ Later in 2014 and 2018, Hardwick’s group conducted two early-phase clinical trials involving a modified vaccinia virus ankara vaccine encoding wtp53 (MVAp53 (**253**)). The studies showed that MVAp53 could induce CD8+ and CD4+ T cell responses in patients with refractory gastrointestinal cancer and ovarian cancer, respectively. Currently, the combination of MVAp53 and an anti-PD1 antibody, pembrolizumab, is being tested in clinical trials (NCT03113487, NCT02432963).^591–594^ In researches from Antonia’s and Chiappori’s group, a modified autologous dendritic cells expressing p53 peptides (DC-p53 (**254**)) was used as a p53 vaccine and applied in combination with chemotherapy in patients with small cell lung cancer (SCLC).^595,596^ Although objective clinical responses were observed in some patients, the phase II randomized trial in 2019 of salvage chemotherapy after immunization with DC-p53 in recurrent SCLC failed to meet expectations.^597^

In 2001, Gjertsen’s group conducted a phase I/II trial (CTN RAS 95002, CTN RAS 97004) to evaluate the clinical benefit of a synthetic mutant RAS peptides-based vaccine, compound (**255**), combined with granulocyte-macrophage colony-stimulating factor in patients with adenocarcinoma of the pancreas.^598,599^ The trial demonstrated a desirable association between prolonged survival and an immune response caused by the RAS peptide vaccination. In 2017, Palmer et al. reported the results of a phase I/II trial (NCT02261714) conducted by Targovax ASA, which demonstrated that a mixture of seven RAS peptides, called TG01 (**256**), could induce RAS mutant-specific T-cell responses that could be enhanced by co-administration of GM-CSF in patients with resected RAS-mutant adenocarcinoma of the pancreas.^600^ Targovax ASA subsequently sponsored another clinical trial of an upgraded version of the TG01 vaccine, called TG02 (**257**), to evaluate its safety and immune activation in patients with locally advanced primary and recurrent oncogenic RAS exon 2 mutant colorectal cancer (NCT02933944). However, the latest update posted on January 26, 2022 reported undesirable safety and moderate immune status.^601^

### Immune system engineering

Engineering the immune system to recognize antigens that are specific to mutant undruggable proteins has emerged as another promising approach to treating undruggable protein-driven diseases. This approach involves utilizing specific antibodies and adoptive cell therapy to target these antigens.

Adoptive cell therapy is an immunotherapy that involves genetically modifying T cells to express either a chimeric antigen receptor or T cell receptor (TCR) that can recognize specific antigens displayed on tumors, leading to clinical benefits.^602,603^ CD8^+^ TILs and TCRs in CD4^+^ T cells have been found to specifically recognize KRAS-related mutations, which can be further used to engineer T cells.^604,605^ In 2016, Wang and co-workers isolated highly reactive T-cell receptors (TCR) to mutated KRAS variants G12V and G12D from murine T cells in immunized HLA-A*11:01 (**258**) transgenic mice, which were then transduced into peripheral blood lymphocytes (PBLs). The transduced PBLs could recognize multiple HLA-A*11:01(+) tumor lines bearing the appropriate KRAS mutations. Adoptive transfer of these transduced PBLs resulted in a significant reduction in tumor growth in a xenograft model by detecting human KRAS-mutant pancreatic cells.^606^ Currently, two phase I/II clinical trials using engineered TCRs are recruiting patients with cancer that has the KRAS^G12V^ or KRAS^G12D^ molecule on the surface of tumors, to determine if anti-KRAS^G12D^ mTCR- or anti-KRAS^G12V^ mTCR-transduced PBLs can mediate the regression of tumors harboring the corresponding RAS mutation (NCT03745326, NCT03190941).

TCR-like antibodies, also known as TCR mimic antibodies, provide another promising approach to targeting intracellular undruggable proteins by engineering immune system. These antibodies recognize epitopes displayed by MHC class I on the cell surface. In 2017, Li and Banham’s group innovatively generated a TCR mimic antibody, T1-116C (**259**), that recognize a p53-derived epitope selectively displayed on MHC class I only by cancer cells, inducing tumor regression in mice with breast cancer xenografts.^607^ Later in 2019, Wang’s group developed another p53-specific TCR-like antibody, P1C1TM (**260**), that can induce cellular cytotoxicity towards cancer cells bearing p53 mutations dependent on selective antibody.^608^ Bispecific antibodies are another type of specific antibody that shows promise in cancer immunotherapy. In 2021, Zhou and co-workers generated a single-chain mutp53-based bispecific antibody, compound (**261**), that can recognize a neoantigen derived from the p53 (R175H) hot-spot mutant and the TCR-CD3 complex. The tight coupling between such bispecific antibody and the p53 (R175H) peptide-HLA complex on tumor cells and TCR-CD3 complex on T cells ameliorate the hampered immune elimination caused by the original low density of neoantigens. As a consequence, the bispecific antibody can selectively redirect T cells to recognize cancer cells presenting the mutant peptide and exhibited selective cytotoxicity against p53 (R175H)-expressing cancer cells, providing a promising approach for immunotherapy.^609^

### Undruggable proteins and other immunotherapy

It is widely accepted that gene mutations, including those affecting undruggable proteins, have a significant impact on the efficacy of cancer immunotherapy. These mutations can affect the tumor immune microenvironment (TIME) by altering cytokine expression and regulating the tumor’s sensitivity to immune checkpoint inhibitors. For examples, cancer cells with functional wild-type genes tend to have a desirable cancer-restrictive TIME, while an imbalance in their expression can create a cancer-supportive TIME.^610,611^ Some missense mutant proteins can lose the transcriptional effects of wild-type genes, such as keeping PDL1 expression levels low, thereby making cancer cells more susceptible to cytotoxic T cells and NK cells, limiting the immune system’s ability to attack cancer cells. On the other hand, activating wild-type proteins can increase IFNγ signaling and sensitize tumors to immune checkpoint inhibitors, whereas corresponding mutant proteins can reduce the efficacy of immune checkpoint inhibitors.^612–614^ In brief, the status of genes can influence tumor cells not only through the impact of wild-type gene loss but also through their effects on the TIME.

Therefore, combination of drugs that restore or boost wild-type gene functionality in cancer cells and immunotherapy regimens has a higher likelihood of success compared to monotherapies. Tumor cells can evade detection of the immune system through various checkpoints, including cytotoxic T lymphocyte protein 4 (CTLA4), PD1 and PDL1. As a result, immune checkpoint inhibitors have been approved for use in immunotherapy. A phase II clinical trial (NCT03600883) is underway to investigate the combination of AMG 510 and anti-PD1 or anti-PDL1 for the treatment of NSCLC, as AMG 510 has been shown to synergize with anti-PD1 treatment. Additionally, a phase I clinical trial (NCT04000529) is evaluating the combination of a SHP2 inhibitor, TNO155, and an anti-PD1 antibody, spartalizumab. Recently, in July 2022, PMV Pharmaceuticals declared a clinical trial collaboration with Merck of PC14586 in combination with the immune checkpoint inhibitor pembrolizumab (KEYTRUDA), in patients with advanced solid tumors that carry the p53(Y220C) mutation.

Of note, as non-cancer cells in the tumor microenvironment (TME) retain wild-type genes, and combinations of wild-type gene activators with immunotherapy are emerging as potential treatment options. APG-115 is an MDM2 inhibitor could activate p53 through MDM2-p53 interaction, and its combination with pembrolizumab has been shown good tolerability and preliminary indications of antitumor activity in a clinical trial for patients with metastatic melanoma and advanced-stage solid tumors (NCT03611868).^403,405^ Besides, the synergistic effects of p53 activation and immunotherapy have also been verified in gene therapy modules, providing promising evidence for further attempts at combined application.^615^

## Others

### Targeting upstream/downstream effectors or cofactors

As some undruggable protein targets are involved in intricate biological networks and signal pathways, regulating their relevant signaling pathways by targeting their upstream or downstream proteins has become an alternative approach to indirectly target undruggable proteins. Take RAS for example, there are more than 11 RAS effector families that have been identified so far. Inactivation of RAS upstream receptor tyrosine kinases, such as EGFR family, can reduce RAS activation, making inhibition of EGFR an effective way to treat RAS-mutant tumors. On the other hand, MAPK and PI3K are effector pathways which could be respectively activated by RAS, and complete suppression of MAPK pathway can help treat RAS-mutant tumors.^616,617^ As a consequence, RAF inhibitors, MEK inhibitors and ERK inhibitors can be used in combinations with other inhibitors to indirectly target mutant-RAS due to the failures of monotherapies.^618,619^ Despite attempts to verify the efficacy of combined inhibition of PI3K and MEK, no desirable clinical result has been achieved for mutant-RAS tumors, and none of the approved PI3K inhibitors is adapted to treat them.^620–622^ Additionally, targeting TFs is a promising approach to modulate dysregulated transcription, as their action is highly related to chemical perturbation from individual general transcriptional cofactors, such as transcriptional kinases, epigenetic proteins, and co-activators. CDK9 inhibitor KB-0742 and BRD4 bromodomain 2 (BD2) inhibitor ABBV-744 have shown high selectivity in transcriptional programs, making them attractive candidates for therapeutic development.^623,624^

### Inducing synthetic lethality

Synthetic lethality is a concept that originated from classical genetics, referring to a pathomechanism in which simultaneous mutation of two genes leads to cell death, but mutation of either gene alone is compatible with viability. In other words, if one of synthetic lethality pair genes is dysregulated, the other one might be essential for survival. Therefore, identifying genes that are synthetically lethal with undruggable proteins driven by mutant gene is of great significance. For instance, *RAF1* and *SHOC2*, which encode CRAF and SHOC2 respectively, have been shown to be important in *KRAS*-mutant cell lines, suggesting inhibition of CRAF and SHOC2 could be used in *KRAS*-mutant cancer. In the case of Myc, there has already been a phase I clinical trial concerning synthetic lethality of Myc by assessing the safety and tolerability of weekly dinaciclib in combination with pembrolizumab in patients with advanced breast cancer (NCT01676753). In Myc-overexpressing triple-negative breast cancer (TNBC) xenografts, inhibition of cyclin-dependent kinase 1 (CDK1) with dinaciclib leads to synthetic lethality and attenuates distant metastasis. In the latest progress report of this ongoing clinical trial, the toxicities were reported to be generally manageable and non-overlapping. Similarly, checkpoint kinase 1 (CHK1) inhibitors^625–627^ and glutaminase (GLS) inhibitors^628–634^ are also considered potential candidates for treating Myc-overexpressing cancers. High-throughput siRNA screening in 2012 Grandori’s group identified a network of genes required for survival of c-Myc overexpressing cells, among which *CAMK2G* was screened out and further verified as the most potential gene in such networks. Later, Ca^2+^/calmodulin-dependent protein kinase II γ (CAMKIIγ), which was encoded by *CAMK2G*, was proven to be an essential target to inhibit T cell lymphoma by destabilizing c-Myc. Inhibiting CAMKIIγ with its specific inhibitor berbamine could suppress development of T cell lymphoma and reduce tumor burden.^635,636^

### Targeting post-translational modifications (PTMs)

Multiple post-translational modifications (PTMs), such as ubiquitination, hydroxylation, methylation, acetylation, and phosphorylation, play critical roles in orchestrating the activities of undruggable TFs, including protein stability, subcellular localization, PPIs and sequence-specific DNA binding. Hence, targeting PTMs provides an alternative approach to regulate undruggable TFs thus treat relevant disease. For instance, inhibition of JAKs is an effective way to block aberrant activation of JAK/STAT pathway in various immune-mediated diseases and cancers, as STATs can be phosphorylated by JAKs. As such, inhibition of HIF-α hydroxylation can be achieved using PHD inhibitors, such as Roxadustat, which is approved in China and Europe for the treatment of renal anemia in CKD, and other PHD inhibitors, daprodustat, vadadustat, enarodustat and molidustat, which have been approved in Japan.^637–642^

Besides, the exploration of these modifications could lay the foundation for targeting the PTMs. In 2014, a proteomics study identified 222 PTMs of 99 residues on p53, a tumor suppressor protein. These modifications can alter the DNA binding activity and cofactor interactions of TFs, representing highly tractable targets for modulation of TF activity.^643,644^

### Designing conjugates

Diverse conjugates have been developed to incorporate desirable properties, such as effective delivery function and cytotoxic activity, by combining various types of molecules, including antibodies, drugs, and small molecules, into a single chemical entity. This provides an attractive approach to rationally design potential compounds that can target undruggable proteins. Various types of conjugates, including antibody-drug conjugates (ADCs), antibody-siRNA conjugates (ARCs), small molecule-drug conjugates (SMDCs) and small molecule-assisted receptor targeting (SMART), have been designed as inhibitors of undruggable protein. ADCs, in particular, have become a mainstream type of conjugate that has been successfully used in cancer treatment. Composed of cytotoxic drugs and monoclonal antibodies linked by chemical linkers, ADCs combine the activity of small-molecule drugs and the delivery function of antibodies. They bind to cell surface receptors that are upregulated by mutant RAS and exhibit potent antitumor activity, demonstrating their efficacy in targeting undruggable proteins outside the cell.^645–649^ P1C1TM is a p53-specific TCR-like antibody, which has been verified by Wang’s group to be suitable for promoting drug delivery into tumor cells with p53 mutation via ADCs.^608^ TfR1, an antigen previously considered undruggable, is highly expressed in both tumor cells and normal cells and participate in cellular iron transport. CX-2029 is an ADC that linked a protease-activatable antibody prodrug targeting TfR1 to cytotoxin monomethyl auristatin E (MMAE) using a cysteine protease-cleavable dipeptide as linker. It is designed to be unmasked in tumor microenvironment, resulting in tumor selectivity.^650^ In 2021, Johnson’s group disclosed a phase I (NCT02222922) first-in-human study involving a CX-2029 to evaluate its therapeutic effects on adults with solid tumors.^651^

Despite the significant success achieved in the development of ADCs in recent years, their application is still limited by the low drug loading capacity at safe doses. To overcome this limitation, ARCs have emerged as promising vectors for targeted siRNA delivery, building upon the concept of ADCs and the FDA approval of siRNA drugs.

Furthermore, there are emerging cases where conjugates composed of linked effective compounds and other moieties are utilized as carriers. Lately in 2022, Collisson and Renslo’s group developed an innovative approach by linking an FDA-approved MEK inhibitor with a ferrous iron-activatable drug conjugate (FeADC). This conjugate was based on their discovery that oncogenic KRAS signaling induces early ferrous iron (Fe^2+^) accumulation throughout mutant KRAS, allowing selective blockage of MAPK and targeting of KRAS-driven solid tumors.^652^ This groundbreaking research demonstrates the potential for utilizing conjugates as carriers to target previously undruggable proteins, offering new hope for cancer treatment.

## Concluding remarks and future perspectives

The discovery of drug targets based on an in-depth study of disease mechanisms is essential for the development of effective treatments. Traditionally, an ideal target should possess a well-defined, deep, and narrow binding site with high-affinity ligands, resulting in changed activity, i.e., druggable. However, many key proteins, especially those whose sustained expression is necessary for the maintenance of a pathological state, have been identified as pharmacologically undruggable. Nonetheless, it is widely believed that there is a great potential for success in drugging these undruggable targets. With the continuous advancement and utilization of cutting-edge drug discovery technology, multiple methods have been developed to target undruggable proteins directly or indirectly, including the development of covalent inhibitors, the identification of allosteric sites, targeting PPIs, affecting protein stability, regulating RNA, and modulating immunity. As a result, dozens of molecules have been proven to be effective, with some already on the market. This has transformed what was once considered “undruggable” into “difficult to drug” or “yet to be drugged”. In this review, we have introduced various strategies for drug discovery on undruggable proteins, summarized successful or potential cases of therapeutic entities developed through different approaches, with a focus on drugs that have been approved for marketing or are currently in clinical trials. Undoubtedly, immense progress has been made in the innovation of multiple therapeutic entities and systematic drug design strategies on undruggable proteins, driven by the emergency of new technologies. However, there are still gaps in our understanding that need to be filled.

From aspect of the undruggable protein, continued target discovery paired with deep-going structure identification is crucial for the application of these untouchable but attractive targets in the treatment of difficult miscellaneous diseases. On the one hand, while less than 5% of unique proteins have been validated as drug targets currently, it is foreseeable that the scope of drug targets will be further expanded, including unconventional targets like immune checkpoint proteins, metabolic pathway enzymes, and key proteins in programmed cell death. While tumorigenesis has benefited from pioneering research efforts on mechanism and target discovery, there is an increasing focus on other complex diseases. On the other hand, a lack of accurate structure information of undruggable proteins remains a significant obstacle in the drug discovery. While cryo-electron microscopy (cryo-EM) has made significant progress, low resolutions still need to be addressed. Fortunately, in addition to improved sample preparation and upgraded microscopy, machine-learning and artificial intelligence (AI) algorithms such as AlphaFold2 and RoseTTAFold have achieved significant breakthroughs in analyzing undruggable protein structures, including how they interact with cofactors or molecular chaperones, and the hunting of allosteric sites. These constantly evolving experimental approaches and virtual methods are expected to reveal more targets, illustrate their structures, and facilitate the discovery of drugs targeting them directly or indirectly. Researchers should prioritize hunting for covalent binding sites and allosteric sites, as covalent and allosteric inhibitors have shown rare success in targeting undruggable proteins. In the near future, primary mutant subtypes of undruggable proteins, such as KRAS^G12D^, which currently lack effective drugs, will be significantly impacted.

From the perspective of the drug design strategies, both the objectives and ideas are of extremely pivotal. On the one hand, undruggable proteins have complicated connections in biological networks, so targeting multiple biological processes such as PPIs and RNA participating proteins expression could be effective. As per the undruggable proteins themselves, the emerging concept of targeted covalent inhibition (TCI), allosteric inhibitors and PROTAC facilitate drugging the undruggable by binding or regulating activity of target proteins or degrading them directly. As per biological processes around undruggable proteins, inhibiting their up/downstream effectors or cofactors and interfering their PPIs by targeting other proteins are conventional approaches. As most of existed modulators to undruggable proteins are inhibitors, activators for undruggable proteins like p53 need to be developed. Similarly, although identifying small molecules that modulate PPIs is a focus of research, the development of PPI stabilizers has been less explored. Of note, in addition to efforts on protein level, genetic therapies such as therapeutic RNAs and synthetic lethal screening have also been effective in targeting undruggable proteins indirectly. However, they are limited by off-target effects and side effects, highlighting the need for further research. On the other hand, state-of-the-art technologies biological display technologies, DNA-encoded libraries, fragment-based drug discovery, gene editing, etc., computing science and technologies such as AI, computer-aided drug design, virtual screening, machine learning and deep learning can improve efficiency and save resources. Ideas of drug design and application can be further inspired by designing conjugates to integrate individual drug design ideas thus achieve synergistic effects with a single chemical entity, as well as applying combination with drugs developed by different strategies.

The last but not the least, as increasingly potential compounds are emerging to touch the undruggable, translation to clinical practice needs to be promoted by well-developed efficacy and safety studies. For drugs that are already on the market, upcoming resistance is a major challenge that needs to be foreseen, and protective plans must be developed.

Essential undruggable targets have hampered therapeutic regimens for diseases, but with intended efforts and serendipitous discoveries, these once untouchable proteins have begun to turn into hot therapeutic targets, even being deemed as representative of the cutting edge of drug discovery. Hopefully, the scope of drug targets will expand with future identification of pathogenesis and superior tackling strategies, touching various diseases with promising drugs.

## References

[1] Qin R (2022). Naturally derived indole alkaloids targeting regulated cell death (RCD) for cancer therapy: from molecular mechanisms to potential therapeutic targets. J. Hematol. Oncol..

[2] Gibbs JB (2000). Mechanism-based target identification and drug discovery in cancer research. Science.

[3] Zhang G (2022). Strategies for targeting undruggable targets. Expert Opin. Drug Discov..

[4] Peng F (2022). Regulated cell death (RCD) in cancer: key pathways and targeted therapies. Signal Transduct. Target. Ther..

[5] Ledford H (2022). Cancer drugs are closing in on some of the deadliest mutations. Nature.

[6] Dang CV (2017). Drugging the ‘undruggable’ cancer targets. Nat. Rev. Cancer.

[7] Zorn JA, Wells JA (2010). Turning enzymes ON with small molecules. Nat. Chem. Biol..

[8] Lazo JS, Sharlow ER (2016). Drugging undruggable molecular cancer targets. Annu. Rev. Pharmacol. Toxicol..

[9] Huang L (2021). KRAS mutation: from undruggable to druggable in cancer. Signal Transduct. Target. Ther..

[10] Blair HA (2021). Sotorasib: first approval. Drugs.

[11] Papke B, Der CJ (2017). Drugging RAS: know the enemy. Science.

[12] Moore AR (2020). RAS-targeted therapies: is the undruggable drugged?. Nat. Rev. Drug Discov..

[13] Brautigan DL (2013). Protein Ser/Thr phosphatases-the ugly ducklings of cell signalling. FEBS J..

[14] Fahs S (2016). Approaches to study phosphatases. ACS Chem. Biol..

[15] Sabapathy K, Lane DP (2018). Therapeutic targeting of p53: all mutants are equal, but some mutants are more equal than others. Nat. Rev. Clin. Oncol..

[16] Henley MJ, Koehler AN (2021). Advances in targeting ‘undruggable’ transcription factors with small molecules. Nat. Rev. Drug Discov..

[17] Tao Z, Wu X (2023). Targeting transcription factors in cancer: from “undruggable” to “druggable”. Methods Mol. Biol..

[18] Ye F (2019). Targeting epigenetic machinery: emerging novel allosteric inhibitors. Pharmacol. Ther..

[19] Lu H (2020). Recent advances in the development of protein-protein interactions modulators: mechanisms and clinical trials. Signal Transduct. Target. Ther..

[20] Zhuang JJ (2022). Current strategies and progress for targeting the “undruggable” transcription factors. Acta Pharmacol. Sin..

[21] Pathmanathan S (2022). Drugging the undruggable proteins in cancer: a systems biology approach. Curr. Opin. Chem. Biol..

[22] Bushweller JH (2019). Targeting transcription factors in cancer - from undruggable to reality. Nat. Rev. Cancer.

[23] Wang J (2019). Drug design of “undruggable” targets. Chin. J. Chem..

[24] Han B (2021). Asymmetric organocatalysis: an enabling technology for medicinal chemistry. Chem. Soc. Rev..

[25] Li Z (2021). Perspective of drug design with high-performance computing. Natl Sci. Rev..

[26] Moitessier N (2016). Medicinal chemistry projects requiring imaginative structure-based drug design methods. Acc. Chem. Res..

[27] Chen H (2018). Targeting oncogenic Myc as a strategy for cancer treatment. Signal Transduct. Target. Ther..

[28] Bauer RA (2015). Covalent inhibitors in drug discovery: from accidental discoveries to avoided liabilities and designed therapies. Drug Discov. Today.

[29] Zhang T (2019). Recent advances in selective and irreversible covalent ligand development and validation. Cell Chem. Biol..

[30] Singh J (2011). The resurgence of covalent drugs. Nat. Rev. Drug Discov..

[31] Boike L (2021). Discovery of a functional covalent ligand targeting an intrinsically disordered cysteine within MYC. Cell Chem. Biol..

[32] Li, H. P. et al. A straightforward access to trifluoromethylated natural products through late-stage functionalization. *Nat. Prod. Rep.* **40**, 988–1021 (2023). 10.1039/d2np00056c 36205211

[33] Smith AJ (2009). Beyond picomolar affinities: quantitative aspects of noncovalent and covalent binding of drugs to proteins. J. Med. Chem..

[34] Hagel M (2011). Selective irreversible inhibition of a protease by targeting a noncatalytic cysteine. Nat. Chem. Biol..

[35] Dal-Ré R (2022). Availability of oral antivirals against SARS-CoV-2 infection and the requirement for an ethical prescribing approach. Lancet Infect. Dis..

[36] Liu M (2023). Recent advances on small-molecule bromodomain-containing histone acetyltransferase inhibitors. J. Med. Chem..

[37] Zhu HP (2023). Discovery of tetrahydrofuranyl spirooxindole-based SMYD3 inhibitors against gastric cancer via inducing lethal autophagy. Eur. J. Med. Chem..

[38] Mu J (2021). Discovery of spirooxindole-ferrocene hybrids as novel MDM2 inhibitors. Chin. Chem. Lett..

[39] Herbst RS, Schlessinger J (2019). Small molecule combats cancer-causing KRAS protein at last. Nature.

[40] McCormick F (2015). KRAS as a therapeutic target. Clin. Cancer Res..

[41] Ostrem JM, Shokat KM (2016). Direct small-molecule inhibitors of KRAS: from structural insights to mechanism-based design. Nat. Rev. Drug Discov..

[42] Peeters M (2015). Prevalence of RAS mutations and individual variation patterns among patients with metastatic colorectal cancer: a pooled analysis of randomised controlled trials. Eur. J. Cancer.

[43] Cagir A, Azmi AS (2019). KRAS(G12C) inhibitors on the horizon. Future Med. Chem..

[44] Stephen AG (2014). Dragging ras back in the ring. Cancer Cell.

[45] McCarthy MJ (2019). Discovery of high-affinity noncovalent allosteric KRAS inhibitors that disrupt effector binding. ACS Omega.

[46] Forbes SA (2011). COSMIC: mining complete cancer genomes in the Catalogue of Somatic Mutations in Cancer. Nucleic Acids Res..

[47] Cox AD (2014). Drugging the undruggable RAS: mission possible?. Nat. Rev. Drug Discov..

[48] Visscher M (2016). Covalent targeting of acquired cysteines in cancer. Curr. Opin. Chem. Biol..

[49] Lu S (2016). The structural basis of oncogenic mutations G12, G13 and Q61 in small GTPase K-Ras4B. Sci. Rep..

[50] Boike L (2022). Advances in covalent drug discovery. Nat. Rev. Drug Discov..

[51] Zeng M (2020). Exploring targeted degradation strategy for oncogenic KRAS(G12C). Cell Chem. Biol..

[52] Fakih, M. et al. Phase 1 study evaluating the safety, tolerability, pharmacokinetics (PK), and efficacy of AMG 510, a novel small molecule KRAS(G12c) inhibitor, in advanced solid tumors. *J. Clin. Oncol*. **37**, 3003 (2019).

[53] Skoulidis F (2021). Sotorasib for lung cancers with KRAS p.G12C mutation. N. Engl. J. Med..

[54] Canon J (2019). The clinical KRAS(G12C) inhibitor AMG 510 drives anti-tumour immunity. Nature.

[55] Lanman BA (2020). Discovery of a covalent inhibitor of KRAS(G12C) (AMG 510) for the treatment of solid tumors. J. Med. Chem..

[56] Sotorasib tackles KRASG12C-mutated pancreatic cancer. *Cancer Discov*. **12**, 878–879 (2022). 10.1158/2159-8290.CD-NB2022-0015 35373275

[57] Hallin J (2020). The KRAS(G12C) inhibitor MRTX849 provides insight toward therapeutic susceptibility of KRAS-mutant cancers in mouse models and patients. Cancer Discov..

[58] Fell JB (2020). Identification of the clinical development candidate MRTX849, a covalent KRAS(G12C) inhibitor for the treatment of cancer. J. Med. Chem..

[59] Ni D (2019). Drugging K-Ras(G12C) through covalent inhibitors: mission possible?. Pharmacol. Ther..

[60] Santarpia M (2023). Targeted therapies for KRAS-mutant non-small cell lung cancer: from preclinical studies to clinical development-a narrative review. Transl. Lung Cancer Res..

[61] Jacobio Pharma. Jacobio’s KRAS G12C inhibitor JAB-21822 was granted breakthrough therapy designations by China CDE. http://en.jacobiopharma.com/news/122.html (2022).

[62] Li, A., Li, S., Wang, P., Dang, C. & Liu, D. KRAS mutant protein inhibitor. China patent CN112552295A (2021).

[63] Uprety D, Adjei AA (2020). KRAS: from undruggable to a druggable cancer target. Cancer Treat. Rev..

[64] Nagasaka M (2020). KRAS G12C Game of Thrones, which direct KRAS inhibitor will claim the iron throne?. Cancer Treat. Rev..

[65] Veluswamy R (2021). KRAS G12C-mutant non-small cell lung cancer: biology, developmental therapeutics, and molecular testing. J. Mol. Diagn..

[66] Wang J (2022). Phase I study of JNJ-74699157 in patients with advanced solid tumors harboring the KRAS G12C mutation. Oncologist.

[67] Cascetta, P. et al. KRAS in NSCLC: state of the art and future perspectives. *Cancers* **14**, 5430 (2022). 10.3390/cancers14215430 PMC9656434 36358848

[68] Meng L (2022). Assessment of KRAS G12C target engagement by a covalent inhibitor in tumor biopsies using an ultra-sensitive immunoaffinity 2D-LC-MS/MS approach. Anal. Chem..

[69] Punekar SR (2022). The current state of the art and future trends in RAS-targeted cancer therapies. Nat. Rev. Clin. Oncol..

[70] Dai, X. et al. Heterocyclic compounds, preparation methods and uses thereof. China patent WO2020233592A1 (2020).

[71] Zhe, S. et al. Potent in vivo anti-tumor activity of D-1553 as a single agent and in combination with targeted therapeutics in a broad spectrum of patient-derived xenograft tumor models with KRas G12C mutation. https://www.abstractsonline.com/pp8/#!/9325/presentation/1988 (2021).

[72] Lorthiois E (2022). JDQ443, a structurally novel, pyrazole-based, covalent inhibitor of KRAS(G12C) for the treatment of solid tumors. J. Med. Chem..

[73] Weiss A (2022). Discovery, preclinical characterization, and early clinical activity of JDQ443, a structurally novel, potent, and selective covalent oral inhibitor of KRASG12C. Cancer Discov..

[74] Peng, S.-B. et al. Preclinical characterization of LY3537982, a novel, highly selective and potent KRAS-G12C inhibitor. https://www.abstractsonline.com/pp8/#!/9325/presentation/2344 (2021).

[75] Bröker J (2022). Fragment optimization of reversible binding to the Switch II pocket on KRAS leads to a potent, in vivo active KRAS(G12C) inhibitor. J. Med. Chem..

[76] Rudolph FSAGD (2021). Abstract 1271: In vitro and in vivo characterization of BI 1823911 - a novel KRASG12C selective small molecule inhibitor. Cancer Res..

[77] A phase 1, open-label study of BPI-421286 in subjects with advanced solid tumors. https://clinicaltrials.gov/ct2/show/NCT05315180 (2022).

[78] Nichols, R. J. RMC-6291, a next-generation tri-complex KRASG12C(ON) inhibitor, outperforms KRASG12C(OFF) inhibitors in preclinical models of KRASG12C cancers. https://www.abstractsonline.com/pp8/#!/10517/presentation/14958 (2022).

[79] Ryan, C. IBI351 gets breakthrough therapy designation for KRAS G12C–mutated NSCLC in China. https://www.onclive.com/view/ibi351-gets-breakthrough-therapy-designation-for-kras-g12c-mutated-nsclc-in-china (2023).

[80] Rockville, M. A. S. Innovent anounces NMPA’s breakthrough therapy designation for IBI351 (KRAS G12C inhibitor) as monotherapy for previous treated advanced non-small cell lung cancer. https://www.prnewswire.com/news-releases/innovent-announces-nmpas-breakthrough-therapy-designation-for-ibi351-kras-g12c-inhibitor-as-monotherapy-for-previous-treated-advanced-non-small-cell-lung-cancer-301713957.html (2023).

[81] Tanaka N (2021). Clinical acquired resistance to KRAS(G12C) inhibition through a novel KRAS Switch-II pocket mutation and polyclonal alterations converging on RAS-MAPK reactivation. Cancer Discov..

[82] Knox, J. RMC-9805 (RM-036), a first-in-class, orally-bioavailable, tri-complex covalent KRASG12D(ON) inhibitor, drives profound anti- tumor activity in KRASG12D mutant tumor models. https://s3.us-west-2.amazonaws.com/rvmdpubs.revmed.com/2022/AACR_2022_Knox.pdf (2022).

[83] Singh, M. Indole derivatives as RAS inhibitors in the treatment of cancer. WO2022060836A1 (2022).

[84] Janes MR (2018). Targeting KRAS mutant cancers with a covalent G12C-specific inhibitor. Cell.

[85] Erlanson DA (2000). Site-directed ligand discovery. Proc. Natl Acad. Sci. USA.

[86] Ostrem JM (2013). K-Ras(G12C) inhibitors allosterically control GTP affinity and effector interactions. Nature.

[87] Yang A (2021). The research progress of direct KRAS G12C mutation inhibitors. Pathol. Oncol. Res..

[88] Yang A (2021). Corrigendum: The research progress of direct KRAS G12C mutation inhibitors. Pathol. Oncol. Res..

[89] Patricelli MP (2016). Selective inhibition of oncogenic KRAS output with small molecules targeting the inactive state. Cancer Discov..

[90] Hunter JC (2014). In situ selectivity profiling and crystal structure of SML-8-73-1, an active site inhibitor of oncogenic K-Ras G12C. Proc. Natl Acad. Sci. USA.

[91] Lim SM (2014). Therapeutic targeting of oncogenic K-Ras by a covalent catalytic site inhibitor. Angew. Chem. Int. Ed. Engl..

[92] Xiong Y (2017). Covalent guanosine mimetic inhibitors of G12C KRAS. ACS Med. Chem. Lett..

[93] Zhang Z (2022). Chemical acylation of an acquired serine suppresses oncogenic signaling of K-Ras(G12S). Nat. Chem. Biol..

[94] Zhang Z (2022). Chemoselective covalent modification of K-Ras(G12R) with a small molecule electrophile. J. Am. Chem. Soc..

[95] Zeng M (2017). Potent and selective covalent quinazoline inhibitors of KRAS G12C. Cell Chem. Biol..

[96] Fell JB (2018). Discovery of tetrahydropyridopyrimidines as irreversible covalent inhibitors of KRAS-G12C with in vivo activity. ACS Med. Chem. Lett..

[97] Shin Y (2019). Discovery of N-(1-acryloylazetidin-3-yl)-2-(1H-indol-1-yl)acetamides as covalent inhibitors of KRAS(G12C). ACS Med. Chem. Lett..

[98] Lamnman, B. A. KRASG12C inhibitors and methods of using the same. US2018334454A1 (2018).

[99] Yarden Y, Sliwkowski MX (2001). Untangling the ErbB signalling network. Nat. Rev. Mol. Cell Biol..

[100] Heldin CH (1995). Dimerization of cell surface receptors in signal transduction. Cell.

[101] Yarden Y, Schlessinger J (1987). Epidermal growth factor induces rapid, reversible aggregation of the purified epidermal growth factor receptor. Biochemistry.

[102] Salomon DS (1990). Transforming growth factor-alpha: an oncodevelopmental growth factor. Cancer Cells.

[103] Gschwind A (2004). The discovery of receptor tyrosine kinases: targets for cancer therapy. Nat. Rev. Cancer.

[104] Burgess AW (2008). EGFR family: structure physiology signalling and therapeutic targets. Growth Factors.

[105] Schlessinger J, Lemmon MA (2006). Nuclear signaling by receptor tyrosine kinases: the first robin of spring. Cell.

[106] Scaltriti M, Baselga J (2006). The epidermal growth factor receptor pathway: a model for targeted therapy. Clin. Cancer Res..

[107] Sharma SV (2007). Epidermal growth factor receptor mutations in lung cancer. Nat. Rev. Cancer.

[108] Hynes NE, MacDonald G (2009). ErbB receptors and signaling pathways in cancer. Curr. Opin. Cell Biol..

[109] Kris MG (2003). Efficacy of gefitinib, an inhibitor of the epidermal growth factor receptor tyrosine kinase, in symptomatic patients with non-small cell lung cancer: a randomized trial. JAMA.

[110] Fukuoka M (2023). Multi-institutional randomized phase II trial of gefitinib for previously treated patients with advanced non-small-cell lung cancer. J. Clin. Oncol..

[111] Sequist LV (2011). Genotypic and histological evolution of lung cancers acquiring resistance to EGFR inhibitors. Sci. Transl. Med..

[112] Lynch TJ (2004). Activating mutations in the epidermal growth factor receptor underlying responsiveness of non-small-cell lung cancer to gefitinib. N. Engl. J. Med..

[113] Paez JG (2004). EGFR mutations in lung cancer: correlation with clinical response to gefitinib therapy. Science.

[114] Pao W (2004). EGF receptor gene mutations are common in lung cancers from “never smokers” and are associated with sensitivity of tumors to gefitinib and erlotinib. Proc. Natl Acad. Sci. USA.

[115] Mok TS (2009). Gefitinib or carboplatin-paclitaxel in pulmonary adenocarcinoma. N. Engl. J. Med..

[116] Rosell R (2012). Erlotinib versus standard chemotherapy as first-line treatment for European patients with advanced EGFR mutation-positive non-small-cell lung cancer (EURTAC): a multicentre, open-label, randomised phase 3 trial. Lancet Oncol..

[117] Gazdar AF (2009). Activating and resistance mutations of EGFR in non-small-cell lung cancer: role in clinical response to EGFR tyrosine kinase inhibitors. Oncogene.

[118] Cross DA (2014). AZD9291, an irreversible EGFR TKI, overcomes T790M-mediated resistance to EGFR inhibitors in lung cancer. Cancer Discov..

[119] Pao W, Chmielecki J (2010). Rational, biologically based treatment of EGFR-mutant non-small-cell lung cancer. Nat. Rev. Cancer.

[120] Langer CJ (2013). Epidermal growth factor receptor inhibition in mutation-positive non-small-cell lung cancer: is afatinib better or simply newer?. J. Clin. Oncol..

[121] Sequist LV (2013). Phase III study of afatinib or cisplatin plus pemetrexed in patients with metastatic lung adenocarcinoma with EGFR mutations. J. Clin. Oncol..

[122] Wu YL (2014). Afatinib versus cisplatin plus gemcitabine for first-line treatment of Asian patients with advanced non-small-cell lung cancer harbouring EGFR mutations (LUX-Lung 6): an open-label, randomised phase 3 trial. Lancet Oncol..

[123] Yang JC (2015). Afatinib versus cisplatin-based chemotherapy for EGFR mutation-positive lung adenocarcinoma (LUX-Lung 3 and LUX-Lung 6): analysis of overall survival data from two randomised, phase 3 trials. Lancet Oncol..

[124] Robichaux JP (2021). Structure-based classification predicts drug response in EGFR-mutant NSCLC. Nature.

[125] Smaill JB (2016). Tyrosine kinase inhibitors. 20. Optimization of substituted quinazoline and pyrido[3,4-d]pyrimidine derivatives as orally active, irreversible inhibitors of the epidermal growth factor receptor family. J. Med. Chem..

[126] Ou SH, Soo RA (2015). Dacomitinib in lung cancer: a “lost generation” EGFR tyrosine-kinase inhibitor from a bygone era?. Drug Des. Dev. Ther..

[127] Mok TS (2018). Improvement in overall survival in a randomized study that compared dacomitinib with gefitinib in patients with advanced non-small-cell lung cancer and EGFR-activating mutations. J. Clin. Oncol..

[128] Engelman JA (2007). PF00299804, an irreversible pan-ERBB inhibitor, is effective in lung cancer models with EGFR and ERBB2 mutations that are resistant to gefitinib. Cancer Res..

[129] Roskoski R (2016). Classification of small molecule protein kinase inhibitors based upon the structures of their drug-enzyme complexes. Pharmacol. Res..

[130] Wu YL (2017). Dacomitinib versus gefitinib as first-line treatment for patients with EGFR-mutation-positive non-small-cell lung cancer (ARCHER 1050): a randomised, open-label, phase 3 trial. Lancet Oncol..

[131] Ward RA (2013). Structure- and reactivity-based development of covalent inhibitors of the activating and gatekeeper mutant forms of the epidermal growth factor receptor (EGFR). J. Med. Chem..

[132] Jackson PA (2017). Covalent modifiers: a chemical perspective on the reactivity of α,β-unsaturated carbonyls with thiols via hetero-Michael addition reactions. J. Med. Chem..

[133] Remon J, Planchard D (2015). AZD9291 in EGFR-mutant advanced non-small-cell lung cancer patients. Future Oncol..

[134] Ballard P (2016). Preclinical comparison of osimertinib with other EGFR-TKIs in EGFR-mutant NSCLC brain metastases models, and early evidence of clinical brain metastases activity. Clin. Cancer Res..

[135] Yang JCH (2020). Osimertinib in patients with epidermal growth factor receptor mutation-positive non-small-cell lung cancer and leptomeningeal metastases: the BLOOM Study. J. Clin. Oncol..

[136] Wu YL (2018). CNS efficacy of osimertinib in patients with T790M-positive advanced non-small-cell lung cancer: data from a randomized phase III trial (AURA3). J. Clin. Oncol..

[137] Goss G (2018). CNS response to osimertinib in patients with T790M-positive advanced NSCLC: pooled data from two phase II trials. Ann. Oncol..

[138] Finlay MR (2014). Discovery of a potent and selective EGFR inhibitor (AZD9291) of both sensitizing and T790M resistance mutations that spares the wild type form of the receptor. J. Med. Chem..

[139] Jänne PA (2015). AZD9291 in EGFR inhibitor-resistant non-small-cell lung cancer. N. Engl. J. Med..

[140] Mok TS (2017). Osimertinib or platinum-pemetrexed in EGFR T790M-positive lung cancer. N. Engl. J. Med..

[141] Yang JC (2020). Safety, efficacy, and pharmacokinetics of almonertinib (HS-10296) in pretreated patients with EGFR-mutated advanced NSCLC: a multicenter, open-label, phase 1 trial. J. Thorac. Oncol..

[142] Nagasaka M (2021). Beyond osimertinib: the development of third-generation EGFR tyrosine kinase inhibitors for advanced EGFR + NSCLC. J. Thorac. Oncol..

[143] Park S (2020). EGFR C797S as a resistance mechanism of lazertinib in non-small cell lung cancer with EGFR T790M mutation. Cancer Res. Treat..

[144] Cho BC (2022). MARIPOSA: phase 3 study of first-line amivantamab + lazertinib versus osimertinib in EGFR-mutant non-small-cell lung cancer. Future Oncol..

[145] Heppner DE (2022). Structural basis for inhibition of mutant EGFR with lazertinib (YH25448). ACS Med. Chem. Lett..

[146] Meng J (2022). Metabolic disposition of the EGFR covalent inhibitor furmonertinib in humans. Acta Pharmacol. Sin..

[147] Liu X (2020). Characterization of covalent binding of tyrosine kinase inhibitors to plasma proteins. Drug Metab. Pharmacokinet..

[148] Wu Y (2023). Covalent binding mechanism of furmonertinib and osimertinib with human serum albumin. Drug Metab. Dispos..

[149] Jiang, W. et al. Successful salvage therapy with a high dose of furmonertinib in a case of lung adenocarcinoma harboring EGFR exon 20 insertion. *Am. J. Ther.*10.1097/MJT.0000000000001504 (2022). 10.1097/MJT.0000000000001504 35482932

[150] Gonzalvez F (2021). Mobocertinib (TAK-788): a targeted inhibitor of EGFR exon 20 insertion mutants in non-small cell lung cancer. Cancer Discov..

[151] Han H (2021). Targeting HER2 exon 20 insertion-mutant lung adenocarcinoma with a novel tyrosine kinase inhibitor mobocertinib. Cancer Res..

[152] Kim ES (2016). Olmutinib: first global approval. Drugs.

[153] Attwa MW (2020). Detection and characterization of olmutinib reactive metabolites by LC-MS/MS: elucidation of bioactivation pathways. J. Sep. Sci..

[154] Singh H (2018). U.S. Food and Drug Administration approval: neratinib for the extended adjuvant treatment of early-stage HER2-positive breast cancer. Clin. Cancer Res..

[155] Ma CX (2017). Neratinib efficacy and circulating tumor DNA detection of HER2 mutations in HER2 nonamplified metastatic breast cancer. Clin. Cancer Res..

[156] Li X (2017). Discovery and development of pyrotinib: a novel irreversible EGFR/HER2 dual tyrosine kinase inhibitor with favorable safety profiles for the treatment of breast cancer. Eur. J. Pharm. Sci..

[157] Meng J (2019). Metabolism and disposition of pyrotinib in healthy male volunteers: covalent binding with human plasma protein. Acta Pharmacol. Sin..

[158] Xuhong JC (2019). Mechanism, safety and efficacy of three tyrosine kinase inhibitors lapatinib, neratinib and pyrotinib in HER2-positive breast cancer. Am. J. Cancer Res..

[159] Xu X (2016). AC0010, an irreversible EGFR inhibitor selectively targeting mutated EGFR and overcoming T790M-induced resistance in animal models and lung cancer patients. Mol. Cancer Ther..

[160] Yan X (2018). Promising efficacy of novel BTK inhibitor AC0010 in mantle cell lymphoma. J. Cancer Res. Clin. Oncol..

[161] Xu W (2019). Overcoming resistance to AC0010, a third generation of EGFR inhibitor, by targeting c-MET and BCL-2. Neoplasia.

[162] Huang S (2020). Abivertinib synergistically strengthens the anti-leukemia activity of venetoclax in acute myeloid leukemia in a BTK-dependent manner. Mol. Oncol..

[163] Han L (2021). SH-1028, an irreversible third-generation EGFR TKI, overcomes T790M-mediated resistance in non-small cell lung cancer. Front. Pharmacol..

[164] Xiong A (2022). Efficacy and safety of SH-1028 in patients with EGFR T790M-positive NSCLC: a multicenter, single-arm, open-label, phase 2 trial. J. Thorac. Oncol..

[165] Nilsson B (2005). Gastrointestinal stromal tumors: the incidence, prevalence, clinical course, and prognostication in the preimatinib mesylate era-a population-based study in western Sweden. Cancer.

[166] Wang M (2022). Sunvozertinib, a selective EGFR inhibitor for previously treated non-small cell lung cancer with EGFR exon 20 insertion mutations. Cancer Discov..

[167] Yang Y, Wang Y (2023). Targeting exon 20 insertion mutations in lung cancer. Curr. Opin. Oncol..

[168] Kwon CS (2022). Non-small cell lung cancer with EGFR exon 20 insertion mutation: a systematic literature review and meta-analysis of patient outcomes. Curr. Med. Res. Opin..

[169] Shi Y (2022). Safety, efficacy, and pharmacokinetics of rezivertinib (BPI-7711) in patients with advanced NSCLC with EGFR T790M mutation: a phase 1 dose-escalation and dose-expansion study. J. Thorac. Oncol..

[170] Shi Y (2023). Results of the phase IIa study to evaluate the efficacy and safety of rezivertinib (BPI-7711) for the first-line treatment of locally advanced or metastatic/recurrent NSCLC patients with EGFR mutation from a phase I/IIa study. BMC Med..

[171] Shi, Y. et al. Efficacy and safety of rezivertinib (BPI-7711) in patients with locally advanced or metastatic/recurrent EGFR T790M-mutated NSCLC: a phase 2b study. *J. Thorac. Oncol.* **17**, 1306–1317 (2022). 10.1016/j.jtho.2022.08.015 36049654

[172] Qian, X. P. CK-101 (RX518), a mutant-selective inhibitor of EGFR that overcomes T790M-mediated resistance in NSCLC. https://www.abstractsonline.com/pp8/#!/4292/presentation/3916 (2017). 10.1158/2159-8290.CD-13-0314 PMC4048995 24065731

[173] Lelais, G. et al. Discovery of (R,E)-N-(7-Chloro-1-(1-[4-(dimethylamino)but-2-enoyl]azepan-3-yl)-1H-benzo[d]imidazol-2-yl)-2-methylisonicotinamide (EGF816), a novel, potent, and WT sparing covalent inhibitor of oncogenic (L858R, ex19del) and resistant (T790M) EGFR mutants for the treatment of EGFR mutant non-small-cell lung cancers. J. Med. Chem. 59, 6671–6689 (2016). 10.1021/acs.jmedchem.5b01985 27433829

[174] Jia Y (2016). EGF816 exerts anticancer effects in non-small cell lung cancer by irreversibly and selectively targeting primary and acquired activating mutations in the EGF receptor. Cancer Res..

[175] Jassem J, Dziadziuszko R (2020). Nazartinib in EGFR Thr790Met-mutant non-small-cell lung cancer. Lancet Respir. Med..

[176] Lu X (2018). Targeting EGFR(L858R/T790M) and EGFR(L858R/T790M/C797S) resistance mutations in NSCLC: current developments in medicinal chemistry. Med. Res. Rev..

[177] Xie H (2011). AST1306, a novel irreversible inhibitor of the epidermal growth factor receptor 1 and 2, exhibits antitumor activity both in vitro and in vivo. PLoS ONE.

[178] Jiang JF (2016). [Metabolic research of domestically developed small molecule tyrosine kinase inhibitors]. Yao Xue Xue Bao.

[179] Zhang J (2014). A phase I study of AST1306, a novel irreversible EGFR and HER2 kinase inhibitor, in patients with advanced solid tumors. J. Hematol. Oncol..

[180] Lin L (2014). Metabolism and pharmacokinetics of allitinib in cancer patients: the roles of cytochrome P450s and epoxide hydrolase in its biotransformation. Drug Metab. Dispos..

[181] Zhang H (2014). AST1306, a potent EGFR inhibitor, antagonizes ATP-binding cassette subfamily G member 2-mediated multidrug resistance. Cancer Lett..

[182] Zheng J (2020). First-in-human phase 1 study of ES-072, an oral mutant-selective EGFR T790M inhibitor, in non-small-cell lung cancer. Clin. Lung Cancer.

[183] Wu Y (2021). ARIH1 signaling promotes anti-tumor immunity by targeting PD-L1 for proteasomal degradation. Nat. Commun..

[184] Assessing an oral EGFR inhibitor,YK-209A in patients who have advanced non-small cell lung cancer with EGFR. https://clinicaltrials.gov/ct2/show/NCT05767866 (2023).

[185] YK-029A as first-line treatment versus platinum-based chemotherapy for non-small cell lung cancer (NSCLC) with EGFR exon 20 insertion mutations. https://clinicaltrials.gov/ct2/show/NCT05767892 (2023).

[186] Smaill JB (2000). Tyrosine kinase inhibitors. 17. Irreversible inhibitors of the epidermal growth factor receptor: 4-(phenylamino)quinazoline- and 4-(phenylamino)pyrido[3,2-d]pyrimidine-6-acrylamides bearing additional solubilizing functions. J. Med. Chem..

[187] Djerf Severinsson EA (2011). The pan-ErbB receptor tyrosine kinase inhibitor canertinib promotes apoptosis of malignant melanoma in vitro and displays anti-tumor activity in vivo. Biochem. Biophys. Res. Commun..

[188] McAndrews KM, Kalluri R (2019). Mechanisms associated with biogenesis of exosomes in cancer. Mol. Cancer.

[189] Smee DF (2008). Progress in the discovery of compounds inhibiting orthopoxviruses in animal models. Antivir. Chem. Chemother..

[190] Walter AO (2013). Discovery of a mutant-selective covalent inhibitor of EGFR that overcomes T790M-mediated resistance in NSCLC. Cancer Discov..

[191] Sequist LV (2015). Rociletinib in EGFR-mutated non-small-cell lung cancer. N. Engl. J. Med..

[192] Yan XE (2017). Structural basis of mutant-selectivity and drug-resistance related to CO-1686. Oncotarget.

[193] Zeng F (2020). Rociletinib (CO-1686) enhanced the efficacy of chemotherapeutic agents in ABCG2-overexpressing cancer cells in vitro and in vivo. Acta Pharm. Sin. B.

[194] Murakami H (2018). Clinical activity of ASP8273 in Asian patients with non-small-cell lung cancer with EGFR activating and T790M mutations. Cancer Sci..

[195] Tanaka H (2019). Mutant-selective irreversible EGFR inhibitor, naquotinib, inhibits tumor growth in NSCLC models with EGFR-activating mutations, T790M mutation, and AXL overexpression. Mol. Cancer Ther..

[196] Planken S (2017). Discovery of N-((3 R,4 R)-4-fluoro-1-(6-((3-methoxy-1-methyl-1H-pyrazol-4-yl)amino)-9-methyl-9H-purin-2-yl)pyrrolidine-3-yl)acrylamide (PF-06747775) through structure-based drug design: a high affinity irreversible inhibitor targeting oncogenic EGFR mutants with selectivity over wild-type EGFR. J. Med. Chem..

[197] Murtuza A (2019). Novel third-generation EGFR tyrosine kinase inhibitors and strategies to overcome therapeutic resistance in lung cancer. Cancer Res..

[198] Patel H (2017). Recent updates on third generation EGFR inhibitors and emergence of fourth generation EGFR inhibitors to combat C797S resistance. Eur. J. Med. Chem..

[199] Discafani, C. M. et al. Irreversible inhibition of epidermal growth factor receptor tyrosine kinase with in vivo activity by N-[4-[(3-bromophenyl)amino]-6-quinazolinyl]-2-butynamide (CL-387,785). Biochem. Pharmacol. 57, 917–925 (1999). 10.1016/s0006-2952(98)00356-6 10086326

[200] Sweeney WE (2000). Treatment of polycystic kidney disease with a novel tyrosine kinase inhibitor. Kidney Int..

[201] Greulich H (2005). Oncogenic transformation by inhibitor-sensitive and -resistant EGFR mutants. PLoS Med..

[202] Kobayashi S (2005). An alternative inhibitor overcomes resistance caused by a mutation of the epidermal growth factor receptor. Cancer Res..

[203] Zhou W (2009). Novel mutant-selective EGFR kinase inhibitors against EGFR T790M. Nature.

[204] Fry DW (1994). A specific inhibitor of the epidermal growth factor receptor tyrosine kinase. Science.

[205] Vousden KH, Ryan KM (2009). p53 and metabolism. Nat. Rev. Cancer.

[206] Levine, A. J. Targeting therapies for the p53 protein in cancer treatments. *Annu. Rev. Cancer Biol.* **3**, 21–34 (2019).

[207] Levine AJ (2019). The many faces of p53: something for everyone. J. Mol. Cell. Biol..

[208] Hafner A (2019). The multiple mechanisms that regulate p53 activity and cell fate. Nat. Rev. Mol. Cell Biol..

[209] The Cancer Genome Atlas Research Network. Integrated genomic analyses of ovarian carcinoma. *Nature* **474**, 609–615 (2011). 10.1038/nature10166 PMC3163504 21720365

[210] The Cancer Genome Atlas Research Network. Comprehensive genomic characterization of squamous cell lung cancers. *Nature* **489**, 519–525 (2012). 10.1038/nature11404 PMC3466113 22960745

[211] George J (2015). Comprehensive genomic profiles of small cell lung cancer. Nature.

[212] The Cancer Genome Atlas Research Network. Comprehensive molecular portraits of human breast tumours. *Nature* **490**, 61–70 (2012). 10.1038/nature11412 PMC3465532 23000897

[213] Song Y (2014). Identification of genomic alterations in oesophageal squamous cell cancer. Nature.

[214] Guiley KZ, Shokat KM (2023). A small molecule reacts with the p53 somatic mutant Y220C to rescue wild-type thermal stability. Cancer Discov..

[215] Burgess A (2016). Clinical overview of MDM2/X-targeted therapies. Front. Oncol..

[216] Pazgier M (2009). Structural basis for high-affinity peptide inhibition of p53 interactions with MDM2 and MDMX. Proc. Natl Acad. Sci. USA.

[217] Carvajal, L. A. et al. Dual inhibition of MDMX and MDM2 as a therapeutic strategy in leukemia. *Sci. Transl. Med*. **10**, eaao3003 (2018). 10.1126/scitranslmed.aao3003 PMC6130841 29643228

[218] Ishiba H (2017). Investigation of the inhibitory mechanism of apomorphine against MDM2-p53 interaction. Bioorg. Med. Chem. Lett..

[219] Bista M (2012). On the mechanism of action of SJ-172550 in inhibiting the interaction of MDM4 and p53. PLoS ONE.

[220] Tamura T (2018). Rapid labelling and covalent inhibition of intracellular native proteins using ligand-directed N-acyl-N-alkyl sulfonamide. Nat. Commun..

[221] Tamura T, Hamachi I (2019). Chemistry for covalent modification of endogenous/native proteins: from test tubes to complex biological systems. J. Am. Chem. Soc..

[222] Vassilev LT (2004). In vivo activation of the p53 pathway by small-molecule antagonists of MDM2. Science.

[223] Ueda T (2021). Enhanced suppression of a protein-protein interaction in cells using small-molecule covalent inhibitors based on an N-Acyl-N-alkyl sulfonamide warhead. J. Am. Chem. Soc..

[224] Walensky LD, Gavathiotis E (2011). BAX unleashed: the biochemical transformation of an inactive cytosolic monomer into a toxic mitochondrial pore. Trends Biochem. Sci..

[225] Sattler M (1997). Structure of Bcl-xL-Bak peptide complex: recognition between regulators of apoptosis. Science.

[226] Bruncko M (2015). Structure-guided design of a series of MCL-1 inhibitors with high affinity and selectivity. J. Med. Chem..

[227] Leverson JD (2015). Potent and selective small-molecule MCL-1 inhibitors demonstrate on-target cancer cell killing activity as single agents and in combination with ABT-263 (navitoclax). Cell Death Dis..

[228] Lee S (2016). Allosteric inhibition of antiapoptotic MCL-1. Nat. Struct. Mol. Biol..

[229] Wang J (2023). Selective covalent targeting of pyruvate kinase M2 using arsenous warheads. J. Med. Chem..

[230] Fairlamb AH, Horn D (2018). Melarsoprol resistance in African trypanosomiasis. Trends Parasitol..

[231] Golemovic M (2010). MER1, a novel organic arsenic derivative, has potent PML-RARalpha-independent cytotoxic activity against leukemia cells. Invest. N. Drugs.

[232] Frampton JE (2022). Darinaparsin: first approval. Drugs.

[233] Griffin BA (1998). Specific covalent labeling of recombinant protein molecules inside live cells. Science.

[234] Ni D (2022). Computational elucidation of allosteric communication in proteins for allosteric drug design. Drug Discov. Today.

[235] Ni D (2020). Combining allosteric and orthosteric drugs to overcome drug resistance. Trends Pharmacol. Sci..

[236] Chen I (2013). Allostery through DNA. Nat. Struct. Mol. Biol..

[237] Greener JG, Sternberg MJ (2018). Structure-based prediction of protein allostery. Curr. Opin. Struct. Biol..

[238] Lu S (2014). Recent computational advances in the identification of allosteric sites in proteins. Drug Discov. Today.

[239] Motlagh HN (2014). The ensemble nature of allostery. Nature.

[240] Tsai CJ, Nussinov R (2014). A unified view of “how allostery works”. PLoS Comput. Biol..

[241] Luo MLEA (2023). Research progress of indole-fused derivatives as allosteric modulators: opportunities for drug development. Biomed. Pharmacother..

[242] Gao Y (2014). Central cavity of fructose-1,6-bisphosphatase and the evolution of AMP/fructose 2,6-bisphosphate synergism in eukaryotic organisms. J. Biol. Chem..

[243] Lü, W. et al. Cryo-EM structures of the triheteromeric NMDA receptor and its allosteric modulation. *Science* **355**, eaal3729 (2017). 10.1126/science.aal3729 PMC5568803 28232581

[244] Buzko O, Shokat KM (2002). A kinase sequence database: sequence alignments and family assignment. Bioinformatics.

[245] Yang JS (2012). Rational engineering of enzyme allosteric regulation through sequence evolution analysis. PLoS Comput. Biol..

[246] Gentry PR (2015). Novel allosteric modulators of G protein-coupled receptors. J. Biol. Chem..

[247] Pricer R (2017). From fuzzy to function: the new frontier of protein-protein interactions. Acc. Chem. Res..

[248] Engers DW, Lindsley CW (2013). Allosteric modulation of class C GPCRs: a novel approach for the treatment of CNS disorders. Drug Discov. Today Technol..

[249] Wootten D (2013). Emerging paradigms in GPCR allostery: implications for drug discovery. Nat. Rev. Drug Discov..

[250] Gao ZG, Jacobson KA (2013). Allosteric modulation and functional selectivity of G protein-coupled receptors. Drug Discov. Today Technol..

[251] Foda ZH, Seeliger MA (2014). Kinase inhibitors: an allosteric add-on. Nat. Chem. Biol..

[252] Wu P (2015). Allosteric small-molecule kinase inhibitors. Pharmacol. Ther..

[253] Lu J (2017). KRAS G12C drug development: discrimination between Switch II pocket configurations using hydrogen/deuterium-exchange mass spectrometry. Structure.

[254] Wang X (2022). Identification of MRTX1133, a noncovalent, potent, and selective KRAS(G12D) inhibitor. J. Med. Chem..

[255] Issahaku AR (2022). Characterization of the binding of MRTX1133 as an avenue for the discovery of potential KRAS(G12D) inhibitors for cancer therapy. Sci. Rep..

[256] A noncovalent KRASG12D inhibitor shows potent and selective activity. *Cancer Discov*. **12**, OF8. https://pubmed.ncbi.nlm.nih.gov/36269013 (2022).

[257] Drosten M, Barbacid M (2022). KRAS inhibitors: going noncovalent. Mol. Oncol..

[258] Hallin J (2022). Anti-tumor efficacy of a potent and selective non-covalent KRAS(G12D) inhibitor. Nat. Med..

[259] Neel BG (2003). The ‘Shp’ing news: SH2 domain-containing tyrosine phosphatases in cell signaling. Trends Biochem. Sci..

[260] LaRochelle JR (2016). Structural and functional consequences of three cancer-associated mutations of the oncogenic phosphatase SHP2. Biochemistry.

[261] Simanshu DK (2017). RAS proteins and their regulators in human disease. Cell.

[262] Pádua RAP (2018). Mechanism of activating mutations and allosteric drug inhibition of the phosphatase SHP2. Nat. Commun..

[263] Rehman AU (2019). Gain-of-function SHP2 E76Q mutant rescuing autoinhibition mechanism associated with juvenile myelomonocytic leukemia. J. Chem. Inf. Model..

[264] Zhang RY (2020). Mechanistic insights explain the transforming potential of the T507K substitution in the protein-tyrosine phosphatase SHP2. J. Biol. Chem..

[265] Mullard A (2018). Phosphatases start shedding their stigma of undruggability. Nat. Rev. Drug Discov..

[266] Dance M (2008). The molecular functions of Shp2 in the Ras/mitogen-activated protein kinase (ERK1/2) pathway. Cell. Signal..

[267] Li W (1994). A new function for a phosphotyrosine phosphatase: linking GRB2-Sos to a receptor tyrosine kinase. Mol. Cell. Biol..

[268] Bennett AM (1994). Protein-tyrosine-phosphatase SHPTP2 couples platelet-derived growth factor receptor beta to Ras. Proc. Natl Acad. Sci. USA.

[269] Barford D, Neel BG (1998). Revealing mechanisms for SH2 domain mediated regulation of the protein tyrosine phosphatase SHP-2. Structure.

[270] Tsutsumi R (2018). Off-target inhibition by active site-targeting SHP2 inhibitors. FEBS Open Bio.

[271] Kerr DL (2021). Allosteric SHP2 inhibitors in cancer: targeting the intersection of RAS, resistance, and the immune microenvironment. Curr. Opin. Chem. Biol..

[272] LaMarche MJ (2020). Identification of TNO155, an allosteric SHP2 inhibitor for the treatment of cancer. J. Med. Chem..

[273] Liu C (2021). Combinations with allosteric SHP2 inhibitor TNO155 to block receptor tyrosine kinase signaling. Clin. Cancer Res..

[274] Ma, C. B. Novel heterocyclic derivatives useful as SHP2 inhibitors. WO2017211303A1 (2017).

[275] Bridgewater, N. J. Sanofi’s emerging oncology pipeline highlighted at the AACR Virtual Annual Meeting II. https://www.news.sanofi.us/2020-06-15-Sanofis-emerging-oncology-pipeline-highlighted-at-the-AACR-Virtual-Annual-Meeting-II (2020).

[276] Revolution medicines provides research and development update for its portfolio of novel targeted cancer therapies. https://ir.revmed.com/news-releases/news-release-details/revolution-medicines-provides-research-and-development-update (2020).

[277] Jacobio Pharmaceuticals Group Co., LTD. Jacobio Pipeline. https://www.jacobiopharma.com/en/pipeline/shp2 (2021).

[278] Taylor, A. M. SHP2 phosphatase inhibitors and methods of making and using the same. WO2021061706A1 (2021).

[279] Sun Y (2020). Allosteric SHP2 inhibitor, IACS-13909, overcomes EGFR-dependent and EGFR-independent resistance mechanisms toward osimertinib. Cancer Res..

[280] Martin, L. SHP2 and CDK4/6 inhibitors combination therapies for the treatment of cancer. WO2022271966A1 (2022).

[281] David, P. Combination therapy for treatment of LKB1 deficient cancers. US2022062260A1 (2022).

[282] Zheng, Q. G. Crystalline form of SHP2 inhibitor, and composition thereof, preparation method therefor, and use thereof. WO2021254449A1 (2021).

[283] InnoCare Pharma. https://www.innocarepharma.com/media/1450/innocare-pharma-2020-interim-presentation.pdf (2020).

[284] Wiener JR (1994). Overexpression of the tyrosine phosphatase PTP1B is associated with human ovarian carcinomas. Am. J. Obstet. Gynecol..

[285] Large CH (2009). The relationship between sodium channel inhibition and anticonvulsant activity in a model of generalised seizure in the rat. Epilepsy Res..

[286] Sarver P (2019). 6-Amino-3-methylpyrimidinones as potent, selective, and orally efficacious SHP2 inhibitors. J. Med. Chem..

[287] Chen YN (2016). Allosteric inhibition of SHP2 phosphatase inhibits cancers driven by receptor tyrosine kinases. Nature.

[288] Garcia Fortanet J (2016). Allosteric inhibition of SHP2: identification of a potent, selective, and orally efficacious phosphatase inhibitor. J. Med. Chem..

[289] Stanford SM (2011). Discovery of a novel series of inhibitors of lymphoid tyrosine phosphatase with activity in human T cells. J. Med. Chem..

[290] Frank KJ (2022). Extensive preclinical validation of combined RMC-4550 and LY3214996 supports clinical investigation for KRAS mutant pancreatic cancer. Cell Rep. Med.

[291] Wang RR (2020). Probing the acting mode and advantages of RMC-4550 as an Src-homology 2 domain-containing protein tyrosine phosphatase (SHP2) inhibitor at molecular level through molecular docking and molecular dynamics. J. Biomol. Struct. Dyn..

[292] Chen X (2020). PCC0208023, a potent SHP2 allosteric inhibitor, imparts an antitumor effect against KRAS mutant colorectal cancer. Toxicol. Appl. Pharmacol..

[293] Tang K (2022). Structure-based design, synthesis and biological evaluation of aminopyrazines as highly potent, selective, and cellularly active allosteric SHP2 inhibitors. Eur. J. Med. Chem..

[294] Wang M (2022). SHP2 allosteric inhibitor TK-453 alleviates psoriasis-like skin inflammation in mice via inhibition of IL-23/Th17 axis. iScience.

[295] Conn PJ (2009). Allosteric modulators of GPCRs: a novel approach for the treatment of CNS disorders. Nat. Rev. Drug Discov..

[296] Congreve M (2017). Applying structure-based drug design approaches to allosteric modulators of GPCRs. Trends Pharmacol. Sci..

[297] Conn PJ (2009). Subtype-selective allosteric modulators of muscarinic receptors for the treatment of CNS disorders. Trends Pharmacol. Sci..

[298] Zhang, J. & Nussinov, R. *Protein Allostery in Drug Discovery. Vol. 1163 Advances in Experimental Medicine and Biology* (Springer, 2019).

[299] Liu H (2018). Orthosteric and allosteric action of the C5a receptor antagonists. Nat. Struct. Mol. Biol..

[300] Yano S (2010). Bone metabolism after cinacalcet administration in patients with secondary hyperparathyroidism. J. Bone Miner. Metab..

[301] Baumann M (2022). The potency of selatogrel, a reversible antagonist of the P2Y12 receptor, is affected by calcium concentration. Platelets.

[302] Vourvahis M (2019). No clinical impact of CYP3A5 gene polymorphisms on the pharmacokinetics and/or efficacy of maraviroc in healthy volunteers and HIV-1-infected subjects. J. Clin. Pharmacol..

[303] Arkin MR, Whitty A (2009). The road less traveled: modulating signal transduction enzymes by inhibiting their protein-protein interactions. Curr. Opin. Chem. Biol..

[304] Loregian A, Palù G (2005). Disruption of protein-protein interactions: towards new targets for chemotherapy. J. Cell. Physiol..

[305] Nero TL (2014). Oncogenic protein interfaces: small molecules, big challenges. Nat. Rev. Cancer.

[306] White AW (2008). Protein-protein interactions as targets for small-molecule therapeutics in cancer. Expert Rev. Mol. Med..

[307] Blazer LL, Neubig RR (2009). Small molecule protein-protein interaction inhibitors as CNS therapeutic agents: current progress and future hurdles. Neuropsychopharmacology.

[308] Rosell M, Fernández-Recio J (2018). Hot-spot analysis for drug discovery targeting protein-protein interactions. Expert Opin. Drug Discov..

[309] Buchwald P (2010). Small-molecule protein-protein interaction inhibitors: therapeutic potential in light of molecular size, chemical space, and ligand binding efficiency considerations. IUBMB Life.

[310] Arkin MR, Wells JA (2004). Small-molecule inhibitors of protein-protein interactions: progressing towards the dream. Nat. Rev. Drug Discov..

[311] Díaz-Eufracio BI (2018). Protein-protein interaction modulators for epigenetic therapies. Adv. Protein Chem. Struct. Biol..

[312] Bourgeas R (2010). Atomic analysis of protein-protein interfaces with known inhibitors: the 2P2I database. PLoS ONE.

[313] Basse MJ (2013). 2P2Idb: a structural database dedicated to orthosteric modulation of protein-protein interactions. Nucleic Acids Res..

[314] Higueruelo AP (2013). TIMBAL v2: update of a database holding small molecules modulating protein-protein interactions. Database.

[315] Labbé CM (2013). iPPI-DB: a manually curated and interactive database of small non-peptide inhibitors of protein-protein interactions. Drug Discov. Today.

[316] Axerio-Cilies P (2012). Cheminformatics-driven discovery of selective, nanomolar inhibitors for staphylococcal pyruvate kinase. ACS Chem. Biol..

[317] Cheng SS (2020). The design and development of covalent protein-protein interaction inhibitors for cancer treatment. J. Hematol. Oncol..

[318] Shangary S, Wang S (2009). Small-molecule inhibitors of the MDM2-p53 protein-protein interaction to reactivate p53 function: a novel approach for cancer therapy. Annu. Rev. Pharmacol. Toxicol..

[319] Geppert T (2011). Context-based identification of protein-protein interfaces and “hot-spot” residues. Chem. Biol..

[320] Moreira IS (2007). Hot spots-a review of the protein-protein interface determinant amino-acid residues. Proteins.

[321] Janin J (2008). Protein-protein interaction and quaternary structure. Q. Rev. Biophys..

[322] Shen Q (2016). ASD v3.0: unraveling allosteric regulation with structural mechanisms and biological networks. Nucleic Acids Res..

[323] Akasaka K (1996). Differential structural requirements for interaction of Ras protein with its distinct downstream effectors. J. Biol. Chem..

[324] Wittinghofer A (1997). The interaction of Ras with GTPase-activating proteins. FEBS Lett..

[325] Ullah R (2022). RAF-MEK-ERK pathway in cancer evolution and treatment. Semin. Cancer Biol..

[326] Lu S (2016). Drugging Ras GTPase: a comprehensive mechanistic and signaling structural view. Chem. Soc. Rev..

[327] Lu S (2016). Ras conformational ensembles, allostery, and signaling. Chem. Rev..

[328] Lu S (2016). Inhibitors of Ras-SOS interactions. ChemMedChem.

[329] Spencer-Smith R (2017). Inhibition of RAS function through targeting an allosteric regulatory site. Nat. Chem. Biol..

[330] Koide A (1998). The fibronectin type III domain as a scaffold for novel binding proteins. J. Mol. Biol..

[331] Koide S (2012). Target-binding proteins based on the 10th human fibronectin type III domain (^10^Fn3). Methods Enzymol..

[332] Boriack-Sjodin PA (1998). The structural basis of the activation of Ras by Sos. Nature.

[333] Iversen L (2014). Molecular kinetics. Ras activation by SOS: allosteric regulation by altered fluctuation dynamics. Science.

[334] Zhou C (2022). Discovery of the first-in-class agonist-based SOS1 PROTACs effective in human cancer cells harboring various KRAS mutations. J. Med. Chem..

[335] Schmick M (2014). KRas localizes to the plasma membrane by spatial cycles of solubilization, trapping and vesicular transport. Cell.

[336] Cox AD (2015). Targeting RAS membrane association: back to the future for anti-RAS drug discovery?. Clin. Cancer Res..

[337] Chandra A (2011). The GDI-like solubilizing factor PDEδ sustains the spatial organization and signalling of Ras family proteins. Nat. Cell Biol..

[338] Ismail SA (2011). Arl2-GTP and Arl3-GTP regulate a GDI-like transport system for farnesylated cargo. Nat. Chem. Biol..

[339] Karnoub AE, Weinberg RA (2008). Ras oncogenes: split personalities. Nat. Rev. Mol. Cell Biol..

[340] Pylayeva-Gupta Y (2011). RAS oncogenes: weaving a tumorigenic web. Nat. Rev. Cancer.

[341] Zimmermann G (2013). Small molecule inhibition of the KRAS-PDEδ interaction impairs oncogenic KRAS signalling. Nature.

[342] Leung ELH (2018). Inhibition of KRAS-dependent lung cancer cell growth by deltarasin: blockage of autophagy increases its cytotoxicity. Cell Death Dis..

[343] Papke B (2016). Identification of pyrazolopyridazinones as PDEδ inhibitors. Nat. Commun..

[344] Klein CH (2019). PDEδ inhibition impedes the proliferation and survival of human colorectal cancer cell lines harboring oncogenic KRas. Int. J. Cancer.

[345] Martín-Gago P (2017). Structure-based development of PDEδ inhibitors. Biol. Chem..

[346] Chen D (2019). Fragment-based drug discovery of triazole inhibitors to block PDEδ-RAS protein-protein interaction. Eur. J. Med. Chem..

[347] Leung EL (2019). Identification of a new inhibitor of KRAS-PDEδ interaction targeting KRAS mutant nonsmall cell lung cancer. Int. J. Cancer.

[348] Siddiqui FA (2020). PDE6D inhibitors with a new design principle selectively block K-Ras activity. ACS Omega.

[349] Zhuang C (2012). Discovery, synthesis, and biological evaluation of orally active pyrrolidone derivatives as novel inhibitors of p53-MDM2 protein-protein interaction. J. Med. Chem..

[350] Zhuang C (2014). Double-edged swords as cancer therapeutics: novel, orally active, small molecules simultaneously inhibit p53-MDM2 interaction and the NF-κB pathway. J. Med. Chem..

[351] Sheng C, Zhang W (2013). Fragment informatics and computational fragment-based drug design: an overview and update. Med. Res. Rev..

[352] Sheng C (2015). State-of-the-art strategies for targeting protein-protein interactions by small-molecule inhibitors. Chem. Soc. Rev..

[353] Jiang Y (2017). Structural biology-inspired discovery of novel KRAS-PDEδ inhibitors. J. Med. Chem..

[354] Chen L (2018). Discovery of novel KRAS-PDEδ inhibitors by fragment-based drug design. J. Med. Chem..

[355] Chen L (2022). Discovery of novel KRAS‒PDEδ inhibitors with potent activity in patient-derived human pancreatic tumor xenograft models. Acta Pharm. Sin. B.

[356] Adams JM, Cory S (2018). The BCL-2 arbiters of apoptosis and their growing role as cancer targets. Cell Death Differ..

[357] Kale J (2018). BCL-2 family proteins: changing partners in the dance towards death. Cell Death Differ..

[358] Moldoveanu T (2014). Many players in BCL-2 family affairs. Trends Biochem. Sci..

[359] Cory S, Adams JM (2002). The Bcl2 family: regulators of the cellular life-or-death switch. Nat. Rev. Cancer.

[360] Cory S, Adams JM (2005). Killing cancer cells by flipping the Bcl-2/Bax switch. Cancer Cell.

[361] Souers AJ (2013). ABT-199, a potent and selective BCL-2 inhibitor, achieves antitumor activity while sparing platelets. Nat. Med..

[362] Peirs S (2014). ABT-199 mediated inhibition of BCL-2 as a novel therapeutic strategy in T-cell acute lymphoblastic leukemia. Blood.

[363] Bi C (2017). Inhibition of 4EBP phosphorylation mediates the cytotoxic effect of mechanistic target of rapamycin kinase inhibitors in aggressive B-cell lymphomas. Haematologica.

[364] Aplenc R (2020). Venetoclax for paediatric acute myeloid leukaemia: a step forward. Lancet Oncol..

[365] Wang B (2020). Rational drug design, synthesis, and biological evaluation of novel chiral tetrahydronaphthalene-fused spirooxindole as MDM2-CDK4 dual inhibitor against glioblastoma. Acta Pharm. Sin. B.

[366] Zhu H (2022). Targeting p53-MDM2 interaction by small-molecule inhibitors: learning from MDM2 inhibitors in clinical trials. J. Hematol. Oncol..

[367] Chène P (2003). Inhibiting the p53-MDM2 interaction: an important target for cancer therapy. Nat. Rev. Cancer.

[368] Cheok CF (2011). Translating p53 into the clinic. Nat. Rev. Clin. Oncol..

[369] Vu B (2013). Discovery of RG7112: a small-molecule MDM2 inhibitor in clinical development. ACS Med. Chem. Lett..

[370] Tovar C (2013). MDM2 small-molecule antagonist RG7112 activates p53 signaling and regresses human tumors in preclinical cancer models. Cancer Res..

[371] Verreault M (2016). Preclinical efficacy of the MDM2 inhibitor RG7112 in MDM2-amplified and TP53 wild-type glioblastomas. Clin. Cancer Res..

[372] Jones RJ (2013). The novel anticancer agent JNJ-26854165 induces cell death through inhibition of cholesterol transport and degradation of ABCA1. J. Pharmacol. Exp. Ther..

[373] Chargari C (2011). Preclinical assessment of JNJ-26854165 (Serdemetan), a novel tryptamine compound with radiosensitizing activity in vitro and in tumor xenografts. Cancer Lett..

[374] Smith MA (2012). Initial testing of JNJ-26854165 (Serdemetan) by the pediatric preclinical testing program. Pediatr. Blood Cancer.

[375] Tabernero J (2011). A phase I first-in-human pharmacokinetic and pharmacodynamic study of serdemetan in patients with advanced solid tumors. Clin. Cancer Res..

[376] Kojima K (2010). The novel tryptamine derivative JNJ-26854165 induces wild-type p53- and E2F1-mediated apoptosis in acute myeloid and lymphoid leukemias. Mol. Cancer Ther..

[377] Ravandi F (2016). A phase I trial of the human double minute 2 inhibitor (MK-8242) in patients with refractory/recurrent acute myelogenous leukemia (AML). Leuk. Res..

[378] Wagner AJ (2017). Phase I trial of the human double minute 2 inhibitor MK-8242 in patients with advanced solid tumors. J. Clin. Oncol..

[379] Liao G (2018). The development of piperidinones as potent MDM2-P53 protein-protein interaction inhibitors for cancer therapy. Eur. J. Med. Chem..

[380] Kang MH (2016). Initial testing (stage 1) of MK-8242-A novel MDM2 inhibitor-by the Pediatric Preclinical Testing Program. Pediatr. Blood Cancer.

[381] Wang S (2014). SAR405838: an optimized inhibitor of MDM2-p53 interaction that induces complete and durable tumor regression. Cancer Res..

[382] Hoffman-Luca CG (2015). Elucidation of acquired resistance to Bcl-2 and MDM2 inhibitors in acute leukemia in vitro and in vivo. Clin. Cancer Res..

[383] Canon J (2015). The MDM2 inhibitor AMG 232 demonstrates robust antitumor efficacy and potentiates the activity of p53-inducing cytotoxic agents. Mol. Cancer Ther..

[384] Rew Y, Sun D (2014). Discovery of a small molecule MDM2 inhibitor (AMG 232) for treating cancer. J. Med. Chem..

[385] Taylor A (2021). Phase 1 concentration-QTc and cardiac safety analysis of the MDM2 antagonist KRT-232 in patients with advanced solid tumors, multiple myeloma, or acute myeloid leukemia. Clin. Pharm. Drug Dev..

[386] Zhang X (2020). KRT-232 and navitoclax enhance trametinib’s anti-Cancer activity in non-small cell lung cancer patient-derived xenografts with KRAS mutations. Am. J. Cancer Res..

[387] Sun D (2014). Discovery of AMG 232, a potent, selective, and orally bioavailable MDM2-p53 inhibitor in clinical development. J. Med. Chem..

[388] Holzer P (2015). Discovery of a dihydroisoquinolinone derivative (NVP-CGM097): a highly potent and selective MDM2 inhibitor undergoing phase 1 clinical trials in p53wt tumors. J. Med. Chem..

[389] Weisberg E (2015). Inhibition of wild-type p53-expressing AML by the novel small molecule HDM2 inhibitor CGM097. Mol. Cancer Ther..

[390] Jeay, S. et al. A distinct p53 target gene set predicts for response to the selective p53-HDM2 inhibitor NVP-CGM097. *Elife* **4**, e06498 (2015). 10.7554/eLife.06498 PMC4468608 25965177

[391] Gounder MM (2020). Milademetan, an oral MDM2 inhibitor, in well-differentiated/dedifferentiated liposarcoma: results from a phase 1 study in patients with solid tumors or lymphomas. Eur. J. Cancer.

[392] Arnhold V (2018). Reactivating TP53 signaling by the novel MDM2 inhibitor DS-3032b as a therapeutic option for high-risk neuroblastoma. Oncotarget.

[393] Chapeau EA (2017). Resistance mechanisms to TP53-MDM2 inhibition identified by in vivo piggyBac transposon mutagenesis screen in an Arf(-/-) mouse model. Proc. Natl Acad. Sci. USA.

[394] Furet P (2016). Discovery of a novel class of highly potent inhibitors of the p53-MDM2 interaction by structure-based design starting from a conformational argument. Bioorg. Med. Chem. Lett..

[395] Zhou X (2021). Pharmacologic activation of p53 triggers viral mimicry response thereby abolishing tumor immune evasion and promoting antitumor immunity. Cancer Discov..

[396] Saygin C, Carraway HE (2021). Current and emerging strategies for management of myelodysplastic syndromes. Blood Rev..

[397] Pairawan S (2021). First in class dual MDM2/MDMX inhibitor ALRN-6924 enhances antitumor efficacy of chemotherapy in TP53 wild-type hormone receptor-positive breast cancer models. Breast Cancer Res..

[398] Saleh MN (2021). Phase 1 trial of ALRN-6924, a dual inhibitor of MDMX and MDM2, in patients with solid tumors and lymphomas bearing wild-type TP53. Clin. Cancer Res..

[399] Ding Q (2013). Discovery of RG7388, a potent and selective p53-MDM2 inhibitor in clinical development. J. Med. Chem..

[400] Higgins B (2014). Preclinical optimization of MDM2 antagonist scheduling for cancer treatment by using a model-based approach. Clin. Cancer Res..

[401] Natarajan U (2020). Differential mechanisms involved in RG-7388 and Nutlin-3 induced cell death in SJSA-1 osteosarcoma cells. Cell. Signal..

[402] Reis B (2016). Acute myeloid leukemia patients’ clinical response to idasanutlin (RG7388) is associated with pre-treatment MDM2 protein expression in leukemic blasts. Haematologica.

[403] Fang DD (2021). MDM2 inhibitor APG-115 exerts potent antitumor activity and synergizes with standard-of-care agents in preclinical acute myeloid leukemia models. Cell Death Discov..

[404] Tolcher, A. W. et al. A phase Ib/II study of APG-115 in combination with pembrolizumab in patients with unresectable or metastatic melanomas or advanced solid tumors. *Ann. Oncol*. **30**, I2 (2019).

[405] Yi H (2018). A novel small molecule inhibitor of MDM2-p53 (APG-115) enhances radiosensitivity of gastric adenocarcinoma. J. Exp. Clin. Cancer Res..

[406] Chen H (2017). Restoration of p53 using the novel MDM2-p53 antagonist APG115 suppresses dedifferentiated papillary thyroid cancer cells. Oncotarget.

[407] Cornillie J (2020). Anti-tumor activity of the MDM2-TP53 inhibitor BI-907828 in dedifferentiated liposarcoma patient-derived xenograft models harboring MDM2 amplification. Clin. Transl. Oncol..

[408] Hao, X. et al. BI-907828, a novel potent MDM2 inhibitor, inhibits GBM brain tumor stem cells in vitro and prolongs survival in orthotopic xenograft mouse models. *Neuro Oncol*. **25**, 913–926 (2022). 10.1093/neuonc/noac271 PMC10158121 36521007

[409] Luke, B. ASTX295, a novel small molecule MDM2 antagonist, demonstrates potent activity in AML in combination with decitabine. https://library.ehaweb.org/eha/2020/eha25th/294423/luke.bevan.astx295.a.novel.small.molecule.mdm2.antagonist.demonstrates.potent.html?f=menu%3D6%2Abrowseby%3D8%2Asortby%3D2%2Amedia%3D3%2Ace_id%3D1766%2Aot_id%3D23220 (2020).

[410] Dang CV (2012). MYC on the path to cancer. Cell.

[411] Schaub FX (2018). Pan-cancer alterations of the MYC oncogene and its proximal network across the Cancer Genome Atlas. Cell Syst..

[412] Carroll PA (2018). The MYC transcription factor network: balancing metabolism, proliferation and oncogenesis. Front. Med..

[413] Baluapuri A (2020). Target gene-independent functions of MYC oncoproteins. Nat. Rev. Mol. Cell Biol..

[414] Schaafsma E (2021). MYC activity inference captures diverse mechanisms of aberrant MYC pathway activation in human cancers. Mol. Cancer Res..

[415] Conacci-Sorrell M (2014). An overview of MYC and its interactome. Cold Spring Harb. Perspect. Med..

[416] Mathsyaraja H (2019). Max deletion destabilizes MYC protein and abrogates Eµ-Myc lymphomagenesis. Genes Dev..

[417] Dang CV (2006). The c-Myc target gene network. Semin. Cancer Biol..

[418] Beaulieu, M. E. et al. Pharmacokinetic analysis of omomyc shows lasting structural integrity and long terminal half-life in tumor tissue. *Cancers* **15**, 826 (2023). 10.3390/cancers15030826 PMC9913332 36765784

[419] Demma, M. J. et al. Omomyc reveals new mechanisms to inhibit the MYC oncogene. *Mol. Cell. Biol*. **39**, e00248-19 (2019). 10.1128/MCB.00248-19 PMC6817756 31501275

[420] Jung LA (2017). OmoMYC blunts promoter invasion by oncogenic MYC to inhibit gene expression characteristic of MYC-dependent tumors. Oncogene.

[421] Phase 1/2 study to evaluate safety, pk and efficacy of the MYC-inhibitor OMO-103 in solid tumours (MYCure). https://clinicaltrials.gov/ct2/show/NCT04808362 (2021).

[422] Struntz NB (2019). Stabilization of the Max homodimer with a small molecule attenuates Myc-driven transcription. Cell Chem. Biol..

[423] Jiang H (2009). Stabilizers of the Max homodimer identified in virtual ligand screening inhibit Myc function. Mol. Pharmacol..

[424] Gao J (2022). Comparative analysis of compound NSC13728 as Omomyc homodimer stabilizer by molecular dynamics simulation and MM/GBSA free energy calculation. J. Mol. Model..

[425] Chen X (2021). Targeting WD repeat-containing protein 5 (WDR5): a medicinal chemistry perspective. J. Med. Chem..

[426] Wang ZH (2018). Targeting protein-protein interaction between MLL1 and reciprocal proteins for leukemia therapy. Bioorg. Med. Chem..

[427] Thomas LR (2015). Interaction with WDR5 promotes target gene recognition and tumorigenesis by MYC. Mol. Cell.

[428] Macdonald JD (2019). Discovery and optimization of salicylic acid-derived sulfonamide inhibitors of the WD repeat-containing protein 5-MYC protein-protein interaction. J. Med. Chem..

[429] Chacón Simon S (2020). Discovery of WD repeat-containing protein 5 (WDR5)-MYC inhibitors using fragment-based methods and structure-based design. J. Med. Chem..

[430] Feris EJ (2019). Formation of a structurally-stable conformation by the intrinsically disordered MYC:TRRAP complex. PLoS ONE.

[431] Mödlhammer A (2021). The diarylheptanoid curcumin induces MYC inhibition and cross-links this oncoprotein to the coactivator TRRAP. Front. Oncol..

[432] Feris EJ (2021). Luminescence complementation technology for the identification of MYC:TRRAP inhibitors. Oncotarget.

[433] Nepon-Sixt BS (2019). Myc-driven chromatin accessibility regulates Cdc45 assembly into CMG helicases. Commun. Biol..

[434] Xiong J (2020). STK31 regulates the proliferation and cell cycle of lung cancer cells via the Wnt/β‑catenin pathway and feedback regulation by c‑myc. Oncol. Rep..

[435] Gill MK (2018). A feed forward loop enforces YAP/TAZ signaling during tumorigenesis. Nat. Commun..

[436] Chan P (2016). Autopalmitoylation of TEAD proteins regulates transcriptional output of the Hippo pathway. Nat. Chem. Biol..

[437] Liu-Chittenden Y (2012). Genetic and pharmacological disruption of the TEAD-YAP complex suppresses the oncogenic activity of YAP. Genes Dev..

[438] Li Q (2020). Lats1/2 sustain intestinal stem cells and Wnt activation through TEAD-dependent and independent transcription. Cell Stem Cell.

[439] Tschaharganeh DF (2013). Yes-associated protein up-regulates Jagged-1 and activates the Notch pathway in human hepatocellular carcinoma. Gastroenterology.

[440] Brodowska K (2014). The clinically used photosensitizer Verteporfin (VP) inhibits YAP-TEAD and human retinoblastoma cell growth in vitro without light activation. Exp. Eye Res..

[441] Zhan T (2017). Wnt signaling in cancer. Oncogene.

[442] Miyoshi K, Hennighausen L (2003). Beta-catenin: a transforming actor on many stages. Breast Cancer Res..

[443] Chan EF (1999). A common human skin tumour is caused by activating mutations in beta-catenin. Nat. Genet..

[444] Wan X (2012). Activation of β-catenin signaling in androgen receptor-negative prostate cancer cells. Clin. Cancer Res..

[445] Moon RT (2004). WNT and beta-catenin signalling: diseases and therapies. Nat. Rev. Genet..

[446] Tetsu O, McCormick F (1999). Beta-catenin regulates expression of cyclin D1 in colon carcinoma cells. Nature.

[447] Wierstra I, Alves J (2008). The c-myc promoter: still MysterY and challenge. Adv. Cancer Res..

[448] Manegold, P. et al. Differentiation therapy targeting the β-catenin/CBP interaction in pancreatic cancer. *Cancers* **10**, 95 (2018). 10.3390/cancers10040095 PMC5923350 29596326

[449] Dietrich L (2017). Cell permeable stapled peptide inhibitor of Wnt signaling that targets β-catenin protein-protein interactions. Cell Chem. Biol..

[450] Gonsalves FC (2011). An RNAi-based chemical genetic screen identifies three small-molecule inhibitors of the Wnt/wingless signaling pathway. Proc. Natl Acad. Sci. USA.

[451] Li X (2013). Henryin, an ent-kaurane diterpenoid, inhibits Wnt signaling through interference with β-catenin/TCF4 interaction in colorectal cancer cells. PLoS ONE.

[452] Sukhdeo K (2007). Targeting the beta-catenin/TCF transcriptional complex in the treatment of multiple myeloma. Proc. Natl Acad. Sci. USA.

[453] Cummins JM (2004). X-linked inhibitor of apoptosis protein (XIAP) is a nonredundant modulator of tumor necrosis factor-related apoptosis-inducing ligand (TRAIL)-mediated apoptosis in human cancer cells. Cancer Res..

[454] Hunter AM (2007). The inhibitors of apoptosis (IAPs) as cancer targets. Apoptosis.

[455] Rumble JM, Duckett CS (2008). Diverse functions within the IAP family. J. Cell Sci..

[456] Gyrd-Hansen M, Meier P (2010). IAPs: from caspase inhibitors to modulators of NF-kappaB, inflammation and cancer. Nat. Rev. Cancer.

[457] Fulda S, Debatin KM (2006). Extrinsic versus intrinsic apoptosis pathways in anticancer chemotherapy. Oncogene.

[458] Huang Y (2003). Requirement of both the second and third BIR domains for the relief of X-linked inhibitor of apoptosis protein (XIAP)-mediated caspase inhibition by Smac. J. Biol. Chem..

[459] Flygare JA (2012). Discovery of a potent small-molecule antagonist of inhibitor of apoptosis (IAP) proteins and clinical candidate for the treatment of cancer (GDC-0152). J. Med. Chem..

[460] Tolcher AW (2016). A phase I dose-escalation study evaluating the safety tolerability and pharmacokinetics of CUDC-427, a potent, oral, monovalent IAP antagonist, in patients with refractory solid tumors. Clin. Cancer Res..

[461] Bardia, A. et al. Paclitaxel with inhibitor of apoptosis antagonist, LCL161, for localized triple-negative breast cancer, prospectively stratified by gene signature in a biomarker-driven neoadjuvant trial. *J. Clin. Oncol*. 10.1200/JCO.2017.74.8392 (2018). 10.1200/JCO.2017.74.8392 30235087

[462] Cai Q (2011). A potent and orally active antagonist (SM-406/AT-406) of multiple inhibitor of apoptosis proteins (IAPs) in clinical development for cancer treatment. J. Med. Chem..

[463] Allensworth JL (2013). Smac mimetic Birinapant induces apoptosis and enhances TRAIL potency in inflammatory breast cancer cells in an IAP-dependent and TNF-α-independent mechanism. Breast Cancer Res. Treat..

[464] Smyth T (2016). Abstract 1287: The dual IAP antagonist, ASTX660, increases the anti-tumor activity of paclitaxel in preclinical models of triple-negative breast cancer in vivo. Cancer Res..

[465] Leung CH (2013). DNA-binding small molecules as inhibitors of transcription factors. Med. Res. Rev..

[466] Gniazdowski M (2003). Transcription factors as targets for DNA-interacting drugs. Curr. Med. Chem..

[467] Gniazdowski M (2005). Effects of anticancer drugs on transcription factor-DNA interactions. Expert Opin. Ther. Targets.

[468] Dokholyan NV (2016). Controlling allosteric networks in proteins. Chem. Rev..

[469] Chennubhotla C, Bahar I (2007). Signal propagation in proteins and relation to equilibrium fluctuations. PLoS Comput. Biol..

[470] Chennubhotla C (2008). Coupling between global dynamics and signal transduction pathways: a mechanism of allostery for chaperonin GroEL. Mol. Biosyst..

[471] Wang H (2015). Direct inhibition of c-Myc-Max heterodimers by celastrol and celastrol-inspired triterpenoids. Oncotarget.

[472] Carabet, L. A. et al. Therapeutic inhibition of Myc in cancer. Structural bases and computer-aided drug discovery approaches. *Int. J. Mol. Sci*. **20**, 120 (2018). 10.3390/ijms20010120 PMC6337544 30597997

[473] Ton, A. T. et al. Development of VPC-70619, a small-molecule N-Myc inhibitor as a potential therapy for neuroendocrine prostate cancer. *Int. J. Mol. Sci*. **23**, 2588 (2022). 10.3390/ijms23052588 PMC8910697 35269731

[474] Yao R (2022). Novel dual-targeting c-Myc inhibitor D347-2761 represses myeloma growth via blocking c-Myc/Max heterodimerization and disturbing its stability. Cell Commun. Signal.

[475] Xie Y (2019). The effect of selective serotonin reuptake inhibitors on cognitive function in patients with Alzheimer’s disease and vascular dementia: focusing on fluoxetine with long follow-up periods. Signal Transduct. Target. Ther..

[476] He M (2022). PROTACs: great opportunities for academia and industry (an update from 2020 to 2021). Signal Transduct. Target. Ther..

[477] Cao C (2022). Chemistries of bifunctional PROTAC degraders. Chem. Soc. Rev..

[478] Mullard A (2021). Targeted protein degraders crowd into the clinic. Nat. Rev. Drug Discov..

[479] Santos R (2017). A comprehensive map of molecular drug targets. Nat. Rev. Drug Discov..

[480] Gu S (2018). PROTACs: an emerging targeting technique for protein degradation in drug discovery. Bioessays.

[481] Sakamoto KM (2001). Protacs: chimeric molecules that target proteins to the Skp1-Cullin-F box complex for ubiquitination and degradation. Proc. Natl Acad. Sci. USA.

[482] Chirnomas D (2023). Protein degraders enter the clinic - a new approach to cancer therapy. Nat. Rev. Clin. Oncol..

[483] Crews CM (2010). Targeting the undruggable proteome: the small molecules of my dreams. Chem. Biol..

[484] Zhu C (2022). Targeting KRAS mutant cancers: from druggable therapy to drug resistance. Mol. Cancer.

[485] Anthony, W. T. et al. Trial in progress: a phase 1, first-in-human, open-label, multicenter, dose-escalation and dose-expansion study of ASP3082 in patients with previously treated advanced solid tumors and KRAS G12D mutations. *J. Clin. Oncol.* **41**, TPS764 (2023).

[486] Bond MJ (2020). Targeted degradation of oncogenic KRAS(G12C) by VHL-recruiting PROTACs. ACS Cent. Sci..

[487] Li L (2021). Discovery of KRas G12C-IN-3 and Pomalidomide-based PROTACs as degraders of endogenous KRAS G12C with potent anticancer activity. Bioorg. Chem..

[488] Yang F (2022). Efficient targeted oncogenic KRAS(G12C) degradation via first reversible-covalent PROTAC. Eur. J. Med Chem..

[489] Chipuk JE (2010). The BCL-2 family reunion. Mol. Cell.

[490] King LE (2022). Apoptotic priming is defined by the dynamic exchange of Bcl-2 proteins between mitochondria and cytosol. Cell Death Differ..

[491] Flores-Romero, H. & Garcia-Saez, A. J. The incomplete puzzle of the BCL2 proteins. *Cells* **8**, 1176 (2019). 10.3390/cells8101176 PMC6830314 31569576

[492] Khan S (2019). A selective BCL-X(L) PROTAC degrader achieves safe and potent antitumor activity. Nat. Med..

[493] He Y (2020). Using proteolysis-targeting chimera technology to reduce navitoclax platelet toxicity and improve its senolytic activity. Nat. Commun..

[494] Pal P (2021). Discovery of a novel BCL-X(L) PROTAC degrader with enhanced BCL-2 inhibition. J. Med. Chem..

[495] Lv D (2021). Development of a BCL-xL and BCL-2 dual degrader with improved anti-leukemic activity. Nat. Commun..

[496] Asmamaw MD (2022). A comprehensive review of SHP2 and its role in cancer. Cell Oncol..

[497] Wang M (2020). Discovery of SHP2-D26 as a first, potent, and effective PROTAC degrader of SHP2 protein. J. Med Chem..

[498] Zheng M (2021). Novel PROTACs for degradation of SHP2 protein. Bioorg. Chem..

[499] Yang X (2021). Discovery of thalidomide-based PROTAC small molecules as the highly efficient SHP2 degraders. Eur. J. Med. Chem..

[500] Vemulapalli V (2021). Targeted degradation of the oncogenic phosphatase SHP2. Biochemistry.

[501] Vogelstein B (2000). Surfing the p53 network. Nature.

[502] Feki A, Irminger-Finger I (2004). Mutational spectrum of p53 mutations in primary breast and ovarian tumors. Crit. Rev. Oncol. Hematol..

[503] Yang J (2019). Simple structural modifications converting a bona fide MDM2 PROTAC degrader into a molecular glue molecule: a cautionary tale in the design of PROTAC degraders. J. Med. Chem..

[504] Qi Z (2021). Design and linkage optimization of ursane-thalidomide-based PROTACs and identification of their targeted-degradation properties to MDM2 protein. Bioorg. Chem..

[505] He S (2021). Homo-PROTAC mediated suicide of MDM2 to treat non-small cell lung cancer. Acta Pharm. Sin. B.

[506] Li Y (2019). Discovery of MD-224 as a first-in-class, highly potent, and efficacious proteolysis targeting chimera murine double minute 2 degrader capable of achieving complete and durable tumor regression. J. Med. Chem..

[507] Wang B (2019). Development of selective small molecule MDM2 degraders based on nutlin. Eur. J. Med. Chem..

[508] Wang B (2021). Development of MDM2 degraders based on ligands derived from Ugi reactions: lessons and discoveries. Eur. J. Med. Chem..

[509] Pei H (2022). Targeting key proteins involved in transcriptional regulation for cancer therapy: Current strategies and future prospective. Med. Res. Rev..

[510] Heppler LN, Frank DA (2019). Inhibit versus destroy: are PROTAC degraders the solution to targeting STAT3?. Cancer Cell.

[511] Liu PC (2021). A first-in-class STAT3 degrader KT-333 in development for treatment of hematologic cancers. Blood.

[512] Bai L (2019). A potent and selective small-molecule degrader of STAT3 achieves complete tumor regression in vivo. Cancer Cell.

[513] Wang K (2022). Peptide-based PROTAC degrader of FOXM1 suppresses cancer and decreases GLUT1 and PD-L1 expression. J. Exp. Clin. Cancer Res..

[514] Luo G (2021). Targeting of the FOXM1 oncoprotein by E3 ligase-assisted degradation. J. Med. Chem..

[515] Thompson EB (1998). The many roles of c-Myc in apoptosis. Annu. Rev. Physiol..

[516] Aird, F. et al. Replication study: BET bromodomain inhibition as a therapeutic strategy to target c-Myc. *Elife* **6**, e21253 (2017). 10.7554/eLife.21253 PMC5245966 28100400

[517] Fong CY (2015). BET inhibitor resistance emerges from leukaemia stem cells. Nature.

[518] Wu S (2021). BRD4 PROTAC degrader ARV-825 inhibits T-cell acute lymphoblastic leukemia by targeting ‘undruggable’ Myc-pathway genes. Cancer Cell Int..

[519] Raina K (2016). PROTAC-induced BET protein degradation as a therapy for castration-resistant prostate cancer. Proc. Natl Acad. Sci. USA.

[520] DeMars KM (2018). Selective degradation of BET proteins with dBET1, a proteolysis-targeting chimera, potently reduces pro-inflammatory responses in lipopolysaccharide-activated microglia. Biochem. Biophys. Res. Commun..

[521] Imaide S (2021). Trivalent PROTACs enhance protein degradation via combined avidity and cooperativity. Nat. Chem. Biol..

[522] Xiong Y (2022). Bridged proteolysis targeting chimera (PROTAC) enables degradation of undruggable targets. J. Am. Chem. Soc..

[523] den Besten W, Lipford JR (2020). Prospecting for molecular glues. Nat. Chem. Biol..

[524] Schreiber SL (2021). The rise of molecular glues. Cell.

[525] Zhou N (2019). RUNX proteins desensitize multiple myeloma to lenalidomide via protecting IKZFs from degradation. Leukemia.

[526] Gao S (2020). Novel immunomodulatory drugs and neo-substrates. Biomark. Res..

[527] Berdeja J (2021). A phase 1 study of CFT7455, a novel degrader of IKZF1/3, in multiple myeloma and non-Hodgkin lymphoma. Blood.

[528] Hansen JD (2020). Discovery of CRBN E3 ligase modulator CC-92480 for the treatment of relapsed and refractory multiple myeloma. J. Med. Chem..

[529] Yao G (2020). Cyclin K interacts with beta-catenin to induce Cyclin D1 expression and facilitates tumorigenesis and radioresistance in lung cancer. Theranostics.

[530] Słabicki M (2020). The CDK inhibitor CR8 acts as a molecular glue degrader that depletes cyclin K. Nature.

[531] Lv, L. et al. Discovery of a molecular glue promoting CDK12-DDB1 interaction to trigger cyclin K degradation. *Elife* **9**, e59994 (2020). 10.7554/eLife.59994 PMC7462607 32804079

[532] Liao H (2020). A PROTAC peptide induces durable β-catenin degradation and suppresses Wnt-dependent intestinal cancer. Cell Discov..

[533] Au YZ (2020). Peptide-based PROTAC: the predator of pathological proteins. Cell Chem. Biol..

[534] Marei H (2022). Antibody targeting of E3 ubiquitin ligases for receptor degradation. Nature.

[535] Foley KP (2021). Abstract 971: Chaperone-mediated protein degradation (CHAMP): a novel technology for tumor-targeted protein degradation. Cancer Res..

[536] Li X (2023). Application of novel degraders employing autophagy for expediting medicinal research. J. Med. Chem..

[537] Nixon RA (2013). The role of autophagy in neurodegenerative disease. Nat. Med..

[538] Ciechanover A, Kwon YT (2015). Degradation of misfolded proteins in neurodegenerative diseases: therapeutic targets and strategies. Exp. Mol. Med..

[539] Li Z (2019). Allele-selective lowering of mutant HTT protein by HTT-LC3 linker compounds. Nature.

[540] Qin X (2022). Identification of an autoinhibitory, mitophagy-inducing peptide derived from the transmembrane domain of USP30. Autophagy.

[541] Ji CH (2022). The AUTOTAC chemical biology platform for targeted protein degradation via the autophagy-lysosome system. Nat. Commun..

[542] Takahashi D (2019). AUTACs: cargo-specific degraders using selective autophagy. Mol. Cell.

[543] Pei J (2021). Targeting lysosomal degradation pathways: new strategies and techniques for drug discovery. J. Med. Chem..

[544] Banik SM (2020). Lysosome-targeting chimaeras for degradation of extracellular proteins. Nature.

[545] Caianiello DF (2021). Bifunctional small molecules that mediate the degradation of extracellular proteins. Nat. Chem. Biol..

[546] Pance K (2023). Modular cytokine receptor-targeting chimeras for targeted degradation of cell surface and extracellular proteins. Nat. Biotechnol..

[547] Grohmann C (2022). Development of NanoLuc-targeting protein degraders and a universal reporter system to benchmark tag-targeted degradation platforms. Nat. Commun..

[548] Henning NJ (2022). Deubiquitinase-targeting chimeras for targeted protein stabilization. Nat. Chem. Biol..

[549] Liu J (2022). TF-DUBTACs stabilize tumor suppressor transcription factors. J. Am. Chem. Soc..

[550] Xue Y (2020). Noncoding RNA: from dark matter to bright star. Sci. China Life Sci..

[551] Wang F (2019). Advances in CRISPR-Cas systems for RNA targeting, tracking and editing. Biotechnol. Adv..

[552] Adachi, T. & Nakamura, Y. Aptamers: a review of their chemical properties and modifications for therapeutic application. *Molecules* **24**, 4229 (2019). 10.3390/molecules24234229 PMC6930564 31766318

[553] Crooke ST (2018). RNA-targeted therapeutics. Cell Metab..

[554] Balaji KC (1997). Antiproliferative effects of c-myc antisense oligonucleotide in prostate cancer cells: a novel therapy in prostate cancer. Urology.

[555] Iversen PL (2003). Efficacy of antisense morpholino oligomer targeted to c-myc in prostate cancer xenograft murine model and a Phase I safety study in humans. Clin. Cancer Res..

[556] Sekhon HS (2008). c-MYC antisense phosphosphorodiamidate morpholino oligomer inhibits lung metastasis in a murine tumor model. Lung Cancer.

[557] Alachkar H (2012). Determination of cellular uptake and intracellular levels of Cenersen (Aezea(®), EL625), a p53 antisense oligonucleotide in acute myeloid leukemia cells. J. Pharm. Biomed. Anal..

[558] Bishop MR (1996). Phase I trial of an antisense oligonucleotide OL(1)p53 in hematologic malignancies. J. Clin. Oncol..

[559] Lanasa MC (2012). Phase II study of cenersen, an antisense inhibitor of p53, in combination with fludarabine, cyclophosphamide and rituximab for high-risk chronic lymphocytic leukemia. Leuk. Lymphoma.

[560] Cortes J (2012). Phase 2 randomized study of p53 antisense oligonucleotide (cenersen) plus idarubicin with or without cytarabine in refractory and relapsed acute myeloid leukemia. Cancer.

[561] Nakada Y (2001). Antisense oligonucleotides specific to mutated K-ras genes inhibit invasiveness of human pancreatic cancer cell lines. Pancreatology.

[562] Wang YX (2005). [The study of the effect of antisense oligonucleotide specific to K-ras point mutation on human pancreatic carcinoma cell PC-2]. Zhonghua Wai Ke Za Zhi.

[563] Kita K (1999). Growth inhibition of human pancreatic cancer cell lines by anti-sense oligonucleotides specific to mutated K-ras genes. Int. J. Cancer.

[564] Ross, S. J. et al. Targeting KRAS-dependent tumors with AZD4785, a high-affinity therapeutic antisense oligonucleotide inhibitor of KRAS. *Sci. Transl. Med*. **9**, eaal5253 (2017). 10.1126/scitranslmed.aal5253 28615361

[565] Elbashir SM (2001). Duplexes of 21-nucleotide RNAs mediate RNA interference in cultured mammalian cells. Nature.

[566] Kim DH, Rossi JJ (2007). Strategies for silencing human disease using RNA interference. Nat. Rev. Genet..

[567] Fire A (1998). Potent and specific genetic interference by double-stranded RNA in *Caenorhabditis elegans*. Nature.

[568] Caplen NJ (2001). Specific inhibition of gene expression by small double-stranded RNAs in invertebrate and vertebrate systems. Proc. Natl Acad. Sci. USA.

[569] Elbashir SM (2001). RNA interference is mediated by 21- and 22-nucleotide RNAs. Genes Dev..

[570] Thielmann M (2021). Teprasiran, a small interfering RNA, for the prevention of acute kidney injury in high-risk patients undergoing cardiac surgery: a randomized clinical study. Circulation.

[571] Ramot Y (2016). Preclinical safety evaluation in rats of a polymeric matrix containing an siRNA drug used as a local and prolonged delivery system for pancreatic cancer therapy. Toxicol. Pathol..

[572] US Securities and Exchange Commission. Dicerna Pharmaceuticals, Inc. Form 10-K. http://www.sec.gov/Archives/edgar/data/1399529/000119312515089221/d851674d10k.htm (2015).

[573] Martinez LA (2002). Synthetic small inhibiting RNAs: efficient tools to inactivate oncogenic mutations and restore p53 pathways. Proc. Natl Acad. Sci. USA.

[574] Ubby I (2019). Cancer therapeutic targeting using mutant-p53-specific siRNAs. Oncogene.

[575] Kabilova TO (2006). [Silencing of c-myc gene expression by enzymatically and chemically synthesized siRNAs]. Mol. Biol..

[576] Zhang X (2009). The knockdown of c-myc expression by RNAi inhibits cell proliferation in human colon cancer HT-29 cells in vitro and in vivo. Cell. Mol. Biol. Lett..

[577] Napoli S (2009). Promoter-specific transcriptional interference and c-myc gene silencing by siRNAs in human cells. EMBO J..

[578] Ai ZH (2013). Suppression of RNA interference on expression of c-myc of SKOV3 ovarian carcinoma cell line. Eur. Rev. Med. Pharmacol. Sci..

[579] Lee W (2018). Selective targeting of KRAS oncogenic alleles by CRISPR/Cas9 inhibits proliferation of cancer cells. Sci. Rep..

[580] McAndrews, K. M. et al. Exosome-mediated delivery of CRISPR/Cas9 for targeting of oncogenic Kras(G12D) in pancreatic cancer. *Life Sci Alliance* **4**, e202000875 (2021). 10.26508/lsa.202000875 PMC8321670 34282051

[581] Chira S (2018). Restoring the p53 ‘guardian’ phenotype in p53-deficient tumor cells with CRISPR/Cas9. Trends Biotechnol..

[582] Zhan H (2018). Synthesizing a genetic sensor based on CRISPR-Cas9 for specifically killing p53-deficient cancer cells. ACS Synth. Biol..

[583] Seenisamy J (2005). Design and synthesis of an expanded porphyrin that has selectivity for the c-MYC G-quadruplex structure. J. Am. Chem. Soc..

[584] Calabrese DR (2018). Chemical and structural studies provide a mechanistic basis for recognition of the MYC G-quadruplex. Nat. Commun..

[585] Hu MH (2018). Discovery of a new four-leaf clover-like ligand as a potent c-MYC transcription inhibitor specifically targeting the promoter G-quadruplex. J. Med. Chem..

[586] Met O (2011). High immunogenic potential of p53 mRNA-transfected dendritic cells in patients with primary breast cancer. Breast Cancer Res. Treat..

[587] Wei J, Hui AM (2022). The paradigm shift in treatment from Covid-19 to oncology with mRNA vaccines. Cancer Treat. Rev..

[588] Malonis RJ (2020). Peptide-based vaccines: current progress and future challenges. Chem. Rev..

[589] Speetjens FM (2009). Induction of p53-specific immunity by a p53 synthetic long peptide vaccine in patients treated for metastatic colorectal cancer. Clin. Cancer Res..

[590] Leffers N (2012). Long-term clinical and immunological effects of p53-SLP® vaccine in patients with ovarian cancer. Int. J. Cancer.

[591] Hardwick N (2014). Overcoming immunosuppression to enhance a p53MVA vaccine. Oncoimmunology.

[592] Yuan Y (2017). Complete regression of cutaneous metastases with systemic immune response in a patient with triple negative breast cancer receiving p53MVA vaccine with pembrolizumab. Oncoimmunology.

[593] Hardwick NR (2014). p53MVA therapy in patients with refractory gastrointestinal malignancies elevates p53-specific CD8+ T-cell responses. Clin. Cancer Res..

[594] Hardwick NR (2018). p53-Reactive T cells are associated with clinical benefit in patients with platinum-resistant epithelial ovarian cancer after treatment with a p53 vaccine and gemcitabine chemotherapy. Clin. Cancer Res..

[595] Antonia SJ (2006). Combination of p53 cancer vaccine with chemotherapy in patients with extensive stage small cell lung cancer. Clin. Cancer Res..

[596] Chiappori AA (2010). INGN-225: a dendritic cell-based p53 vaccine (Ad.p53-DC) in small cell lung cancer: observed association between immune response and enhanced chemotherapy effect. Expert Opin. Biol. Ther..

[597] Chiappori AA (2019). Randomized-controlled phase II trial of salvage chemotherapy after immunization with a TP53-transfected dendritic cell-based vaccine (Ad.p53-DC) in patients with recurrent small cell lung cancer. Cancer Immunol. Immunother..

[598] Gjertsen MK (1996). Ex vivo ras peptide vaccination in patients with advanced pancreatic cancer: results of a phase I/II study. Int. J. Cancer.

[599] Gjertsen MK (2001). Intradermal ras peptide vaccination with granulocyte-macrophage colony-stimulating factor as adjuvant: clinical and immunological responses in patients with pancreatic adenocarcinoma. Int. J. Cancer.

[600] Palmer DH (2017). A phase I/II trial of TG01/GM-CSF and gemcitabine as adjuvant therapy for treating patients with resected RAS-mutant adenocarcinoma of the pancreas. J. Clin. Oncol..

[601] Goh KC (2012). TG02, a novel oral multi-kinase inhibitor of CDKs, JAK2 and FLT3 with potent anti-leukemic properties. Leukemia.

[602] Robbins PF (1996). A mutated beta-catenin gene encodes a melanoma-specific antigen recognized by tumor infiltrating lymphocytes. J. Exp. Med..

[603] Dudley ME (2002). Cancer regression and autoimmunity in patients after clonal repopulation with antitumor lymphocytes. Science.

[604] Tran E (2016). T-cell transfer therapy targeting mutant KRAS in cancer. N. Engl. J. Med..

[605] Veatch JR (2019). Endogenous CD4(+) T cells recognize neoantigens in lung cancer patients, including recurrent oncogenic KRAS and ERBB2 (Her2) driver mutations. Cancer Immunol. Res..

[606] Wang QJ (2016). Identification of T-cell receptors targeting KRAS-mutated human tumors. Cancer Immunol. Res..

[607] Li D (2017). Development of a T-cell receptor mimic antibody against wild-type p53 for cancer immunotherapy. Cancer Res..

[608] Low L (2019). Targeting mutant p53-expressing tumours with a T cell receptor-like antibody specific for a wild-type antigen. Nat. Commun..

[609] Hsiue, E. H. et al. Targeting a neoantigen derived from a common TP53 mutation. *Science* **371**, eabc8697 (2021). 10.1126/science.abc8697 PMC8208645 33649166

[610] Blagih, J. et al. p53, cancer and the immune response. *J. Cell Sci*. **133**, jcs237453 (2020). 10.1242/jcs.237453 32144194

[611] Levine, A. J. P53 and the immune response: 40 years of exploration-a plan for the future. *Int. J. Mol. Sci*. **21**, 541 (2020). 10.3390/ijms21020541 PMC7013403 31952115

[612] Cortez, M. A. et al. PDL1 regulation by p53 via miR-34. *J. Natl Cancer Inst*. **108**, djv303 (2016). 10.1093/jnci/djv303 PMC4862407 26577528

[613] Textor S (2011). Human NK cells are alerted to induction of p53 in cancer cells by upregulation of the NKG2D ligands ULBP1 and ULBP2. Cancer Res..

[614] Wellenstein MD (2019). Loss of p53 triggers WNT-dependent systemic inflammation to drive breast cancer metastasis. Nature.

[615] Tolcher AW (2021). Preliminary results of a phase II study of alrizomadlin (APG-115), a novel, small-molecule MDM2 inhibitor, in combination with pembrolizumab in patients (pts) with unresectable or metastatic melanoma or advanced solid tumors that have failed immunooncologic (I-O) drugs. J. Clin. Oncol..

[616] Mao M (2013). Resistance to BRAF inhibition in BRAF-mutant colon cancer can be overcome with PI3K inhibition or demethylating agents. Clin. Cancer Res..

[617] Wee S (2009). PI3K pathway activation mediates resistance to MEK inhibitors in KRAS mutant cancers. Cancer Res..

[618] Engelman JA (2008). Effective use of PI3K and MEK inhibitors to treat mutant Kras G12D and PIK3CA H1047R murine lung cancers. Nat. Med..

[619] Hoeflich KP (2012). Intermittent administration of MEK inhibitor GDC-0973 plus PI3K inhibitor GDC-0941 triggers robust apoptosis and tumor growth inhibition. Cancer Res..

[620] Shapiro GI (2020). Phase Ib study of the MEK inhibitor cobimetinib (GDC-0973) in combination with the PI3K inhibitor pictilisib (GDC-0941) in patients with advanced solid tumors. Invest. N. Drugs.

[621] Bedard PL (2015). A phase Ib dose-escalation study of the oral pan-PI3K inhibitor buparlisib (BKM120) in combination with the oral MEK1/2 inhibitor trametinib (GSK1120212) in patients with selected advanced solid tumors. Clin. Cancer Res..

[622] Algazi AP (2019). A dual pathway inhibition strategy using BKM120 combined with vemurafenib is poorly tolerated in BRAF V600(E/K) mutant advanced melanoma. Pigment Cell Melanoma Res..

[623] Richters A (2021). Modulating androgen receptor-driven transcription in prostate cancer with selective CDK9 inhibitors. Cell Chem. Biol..

[624] Faivre EJ (2020). Selective inhibition of the BD2 bromodomain of BET proteins in prostate cancer. Nature.

[625] Cole KA (2011). RNAi screen of the protein kinome identifies checkpoint kinase 1 (CHK1) as a therapeutic target in neuroblastoma. Proc. Natl Acad. Sci. USA.

[626] Murga M (2011). Exploiting oncogene-induced replicative stress for the selective killing of Myc-driven tumors. Nat. Struct. Mol. Biol..

[627] Ferrao PT (2012). Efficacy of CHK inhibitors as single agents in MYC-driven lymphoma cells. Oncogene.

[628] Yuneva M (2007). Deficiency in glutamine but not glucose induces MYC-dependent apoptosis in human cells. J. Cell Biol..

[629] Gao P (2009). c-Myc suppression of miR-23a/b enhances mitochondrial glutaminase expression and glutamine metabolism. Nature.

[630] Mullen AR (2011). Reductive carboxylation supports growth in tumour cells with defective mitochondria. Nature.

[631] Metallo CM (2011). Reductive glutamine metabolism by IDH1 mediates lipogenesis under hypoxia. Nature.

[632] Qing G (2012). ATF4 regulates MYC-mediated neuroblastoma cell death upon glutamine deprivation. Cancer Cell.

[633] Xiao D (2015). Myc promotes glutaminolysis in human neuroblastoma through direct activation of glutaminase 2. Oncotarget.

[634] Jacque N (2015). Targeting glutaminolysis has antileukemic activity in acute myeloid leukemia and synergizes with BCL-2 inhibition. Blood.

[635] Liang Y (2009). Berbamine, a novel nuclear factor kappaB inhibitor, inhibits growth and induces apoptosis in human myeloma cells. Acta Pharmacol. Sin..

[636] Meng Z (2013). Berbamine inhibits the growth of liver cancer cells and cancer-initiating cells by targeting Ca^2+^/calmodulin-dependent protein kinase II. Mol. Cancer Ther..

[637] Filtz TM (2014). Regulation of transcription factor activity by interconnected post-translational modifications. Trends Pharmacol. Sci..

[638] Qian M (2020). Targeting post-translational modification of transcription factors as cancer therapy. Drug Discov. Today.

[639] Svinka J (2014). STAT3 in hepatocellular carcinoma: new perspectives. Hepat. Oncol..

[640] Jamilloux Y (2019). JAK inhibitors for the treatment of autoimmune and inflammatory diseases. Autoimmun. Rev..

[641] Alqahtani, A. et al. Hepatocellular carcinoma: molecular mechanisms and targeted therapies. *Medicina* **55**, 526 (2019). 10.3390/medicina55090526 PMC6780754 31450841

[642] Barrett TD (2011). Pharmacological characterization of 1-(5-chloro-6-(trifluoromethoxy)-1H-benzoimidazol-2-yl)-1H-pyrazole-4-carboxylic acid (JNJ-42041935), a potent and selective hypoxia-inducible factor prolyl hydroxylase inhibitor. Mol. Pharmacol..

[643] DeHart CJ (2014). Extensive post-translational modification of active and inactivated forms of endogenous p53. Mol. Cell. Proteomics.

[644] Blumenthal E (2017). Covalent modifications of RUNX proteins: structure affects function. Adv. Exp. Med. Biol..

[645] Chari RV (2014). Antibody-drug conjugates: an emerging concept in cancer therapy. Angew. Chem. Int. Ed. Engl..

[646] Lambert JM, Morris CQ (2017). Antibody-drug conjugates (ADCs) for personalized treatment of solid tumors: a review. Adv. Ther..

[647] Lamb YN (2017). Glecaprevir/Pibrentasvir: first global approval. Drugs.

[648] Perrino E (2014). Curative properties of noninternalizing antibody-drug conjugates based on maytansinoids. Cancer Res..

[649] Martinko, A. J. et al. Targeting RAS-driven human cancer cells with antibodies to upregulated and essential cell-surface proteins. *Elife* **7**, e31098 (2018). 10.7554/eLife.31098 PMC5796798 29359686

[650] Singh S (2022). Nonclinical efficacy and safety of CX-2029, an anti-CD71 probody-drug conjugate. Mol. Cancer Ther..

[651] Johnson M (2021). Phase I, first-in-human study of the probody therapeutic CX-2029 in adults with advanced solid tumor malignancies. Clin. Cancer Res..

[652] Jiang, H. et al. Ferrous iron-activatable drug conjugate achieves potent MAPK blockade in KRAS-driven tumors. *J. Exp. Med*. **219**, e20210739 (2022). 10.1084/jem.20210739 PMC8916116 35262628

[653] Ascentage Pharma. Proprietary PPI platform delivering potentially first and/or best-in-class drugs. https://www.ascentagepharma.com/wp-content/uploads/2021/01/Ascentage-Pharma-JPM-Deck-20210113-.pdf (2021).

[654] Zuberi M (2020). Inhibition of RAS: proven and potential vulnerabilities. Biochem. Soc. Trans..

[655] Lim, J. E. Form S-1. https://www.sec.gov/Archives/edgar/data/1761918/000119312521200537/d176019ds1.htm (2021).

[656] Meng, J. After the AACR Conference, what new drugs are worth watching? https://baijiahao.baidu.com/s?id=1730615953532632799&wfr=spider&for=pc (2022).

[657] Colloton M (2005). Cinacalcet HCl attenuates parathyroid hyperplasia in a rat model of secondary hyperparathyroidism. Kidney Int..

[658] Dorr P (2005). Maraviroc (UK-427,857), a potent, orally bioavailable, and selective small-molecule inhibitor of chemokine receptor CCR5 with broad-spectrum anti-human immunodeficiency virus type 1 activity. Antimicrob. Agents Chemother..

[659] JJ VANG (2009). Ticagrelor binds to human P2Y(12) independently from ADP but antagonizes ADP-induced receptor signaling and platelet aggregation. J. Thromb. Haemost..

[660] Bekker P (2016). Characterization of pharmacologic and pharmacokinetic properties of CCX168, a potent and selective orally administered complement 5a receptor inhibitor, based on preclinical evaluation and randomized phase 1 clinical study. PLoS ONE.

[661] Walters MJ (2010). Characterization of CCX282-B, an orally bioavailable antagonist of the CCR9 chemokine receptor, for treatment of inflammatory bowel disease. J. Pharmacol. Exp. Ther..

[662] Vranesic I (2014). AFQ056/mavoglurant, a novel clinically effective mGluR5 antagonist: identification, SAR and pharmacological characterization. Bioorg. Med. Chem..

[663] Li X (2003). Allosteric adenosine receptor modulation reduces hypersensitivity following peripheral inflammation by a central mechanism. J. Pharmacol. Exp. Ther..

[664] Justinova Z (2015). The novel metabotropic glutamate receptor 2 positive allosteric modulator, AZD8529, decreases nicotine self-administration and relapse in squirrel monkeys. Biol. Psychiatry.

[665] Zerbib F (2011). Randomised clinical trial: effects of monotherapy with ADX10059, a mGluR5 inhibitor, on symptoms and reflux events in patients with gastro-oesophageal reflux disease. Aliment. Pharmacol. Ther..

[666] Jaeschke, G. et al. Metabotropic glutamate receptor 5 negative allosteric modulators: discovery of 2-chloro-4-[1-(4-fluorophenyl)-2,5-dimethyl-1H-imidazol-4-ylethynyl]pyridine (basimglurant, RO4917523), a promising novel medicine for psychiatric diseases. J. Med. Chem. 58, 1358–1371 (2015). 10.1021/jm501642c 25565255

[667] Cid JM (2014). Discovery of 1-butyl-3-chloro-4-(4-phenyl-1-piperidinyl)-(1H)-pyridone (JNJ-40411813): a novel positive allosteric modulator of the metabotropic glutamate 2 receptor. J. Med. Chem..

[668] Leurquin-Sterk G (2017). What we observe in vivo is not always what we see in vitro: development and validation of 11C-JNJ-42491293, a novel radioligand for mGluR2. J. Nucl. Med..

[669] Uslaner JM (2018). Preclinical to human translational pharmacology of the novel M(1) positive allosteric modulator MK-7622. J. Pharmacol. Exp. Ther..

[670] Sturm S (2018). Results and evaluation of a first-in-human study of RG7342, an mGlu5 positive allosteric modulator, utilizing Bayesian adaptive methods. Br. J. Clin. Pharmacol..

[671] Singhal M (2020). Nanosuspensions of a poorly soluble investigational molecule ODM-106: Impact of milling bead diameter and stabilizer concentration. Int. J. Pharm..

[672] Alonso-De D. S.-A. 6,7-Dihydropyrazolo[1,5-a]pyrazin-4(5H)-one compounds and their use as negative allosteric modulators of mGlur2 receptors. SG11201509938YA (2016).

[673] Tong L (2020). Discovery of [(11)C]MK-6884: a positron emission tomography (PET) imaging agent for the study of M4 muscarinic receptor positive allosteric modulators (PAMs) in neurodegenerative diseases. J. Med. Chem..

[674] Desai A (2021). Phase 1 randomized study on the safety, tolerability, and pharmacodynamic cognitive and electrophysiological effects of a dopamine D(1) receptor positive allosteric modulator in patients with schizophrenia. Neuropsychopharmacology.

[675] Sako Y (2019). TAK-071, a novel M(1) positive allosteric modulator with low cooperativity, improves cognitive function in rodents with few cholinergic side effects. Neuropsychopharmacology.

[676] Charvin D (2017). Discovery, structure-activity relationship, and antiparkinsonian effect of a potent and brain-penetrant chemical series of positive allosteric modulators of metabotropic glutamate receptor 4. J. Med. Chem..

[677] van Till JWO (2022). Muscarinic-3-receptor positive allosteric modulator ASP8302 in patients with underactive bladder. A randomized controlled trial. Neurourol. Urodyn..

[678] Christopher, J. A. et al. Fragment and Structure-Based Drug Discovery for a Class C GPCR: Discovery of the mGlu5 Negative Allosteric Modulator HTL14242 (3-Chloro-5-[6-(5-fluoropyridin-2-yl)pyrimidin-4-yl]benzonitrile. J. Med. Chem. 58, 6653–6664 (2015). 10.1021/acs.jmedchem.5b00892 26225459

[679] Kretz, R. A. Antibodies that bind human cannabinoid 1 (CB1) receptor. WO2015148984A2 (2015).

[680] US Securities and Exchange Commission. Forest Laboratories Form 10-K. http://www.sec.gov/Archives/edgar/data/38074/000003807412000020/forest10k2012.htm (2012).

[681] Rahway, N. J. Merck to hold investor event to highlight oncology portfolio and pipeline. https://www.biospace.com/article/releases/merck-to-hold-investor-event-to-highlight-oncology-portfolio-and-pipeline/ (2022).

[682] Lee, G. J. Use of SOS1 inhibitors with RAS inhibitors to treat cancers. WO2022217053A1 (2022).

[683] Oliver CL (2004). In vitro effects of the BH3 mimetic, (-)-gossypol, on head and neck squamous cell carcinoma cells. Clin. Cancer Res..

[684] Nguyen M (2007). Small molecule obatoclax (GX15-070) antagonizes MCL-1 and overcomes MCL-1-mediated resistance to apoptosis. Proc. Natl Acad. Sci. USA.

[685] Lock R (2008). Initial testing (stage 1) of the BH3 mimetic ABT-263 by the pediatric preclinical testing program. Pediatr. Blood Cancer.

[686] Konopleva M (2006). Mechanisms of apoptosis sensitivity and resistance to the BH3 mimetic ABT-737 in acute myeloid leukemia. Cancer Cell.

[687] Wang J (2017). APG-1252-12A induces mitochondria-dependent apoptosis through inhibiting the antiapoptotic proteins Bcl-2/Bcl-xl in HL-60 cells. Int. J. Oncol..

[688] Lu X (2008). Disruption of the MYC transcriptional function by a small-molecule antagonist of MYC/MAX dimerization. Oncol. Rep..

[689] Berg T (2002). Small-molecule antagonists of Myc/Max dimerization inhibit Myc-induced transformation of chicken embryo fibroblasts. Proc. Natl Acad. Sci. USA.

[690] Yin X (2003). Low molecular weight inhibitors of Myc-Max interaction and function. Oncogene.

[691] Hart JR (2014). Inhibitor of MYC identified in a Kröhnke pyridine library. Proc. Natl Acad. Sci. USA.

[692] Yap JL (2013). Pharmacophore identification of c-Myc inhibitor 10074-G5. Bioorg. Med. Chem. Lett..

[693] Choi SH (2017). Targeted disruption of Myc-Max oncoprotein complex by a small molecule. ACS Chem. Biol..

[694] Castell A (2018). A selective high affinity MYC-binding compound inhibits MYC:MAX interaction and MYC-dependent tumor cell proliferation. Sci. Rep..

[695] Han H (2019). Small-molecule MYC inhibitors suppress tumor growth and enhance immunotherapy. Cancer Cell.

